# Genome-Wide Analysis of the Fasciclin-Like Arabinogalactan Protein Gene Family Reveals Differential Expression Patterns, Localization, and Salt Stress Response in *Populus*

**DOI:** 10.3389/fpls.2015.01140

**Published:** 2015-12-23

**Authors:** Lina Zang, Tangchun Zheng, Yanguang Chu, Changjun Ding, Weixi Zhang, Qinjun Huang, Xiaohua Su

**Affiliations:** ^1^State Key Laboratory of Tree Genetics and Breeding, Northeast Forestry UniversityHarbin, China; ^2^State Key Laboratory of Tree Genetics and Breeding, Research Institute of Forestry, Chinese Academy of ForestryBeijing, China; ^3^Key Laboratory of Tree Breeding and Cultivation, State Forestry AdministrationBeijing, China

**Keywords:** arabinogalactan-proteins, *PtrFLA*, fasciclin domain, expression analysis, subcellular localization, salt response, *Populus trichocarpa*

## Abstract

Fasciclin-like arabinogalactan proteins (FLAs) are a subclass of arabinogalactan proteins (AGPs) involved in plant growth, development and response to abiotic stress. Although many studies have been performed to identify molecular functions of individual family members, little information is available on genome-wide identification and characterization of *FLAs* in the genus *Populus*. Based on genome-wide analysis, we have identified 35 *Populus FLAs* which were distributed on 16 chromosomes and phylogenetically clustered into four major groups. Gene structure and motif composition were relatively conserved in each group. All the members contained N-terminal signal peptide, 23 of which included predicted glycosylphosphatidylinositol (GPI) modification sites and were anchored to plasma membranes. Subcellular localization analysis showed that PtrFLA2/20/26 were localized in cell membrane and cytoplasm of protoplasts from *Populus* stem-differentiating xylem. The *Ka*/*Ks* ratios showed that purifying selection has played a leading role in the long-term evolutionary period which greatly maintained the function of this family. The expression profiles showed that 32 *PtrFLAs* were differentially expressed in four tissues at four seasons based on publicly available microarray data. 18 *FLAs* were further verified with qRT-PCR in different tissues, which indicated that *PtrFLA1/2/3/7/11/12/20/21/22/24/26/30* were significantly expressed in male and female flowers, suggesting close correlations with the reproductive development. In addition, *PtrFLA1/9/10/11/17/21/23/24/26/28* were highly expressed in the stems and differentiating xylem, which may be involved in stem development. To determine salt response of *FLAs*, qRT-PCR was performed to analyze the expression of 18 genes under salinity stress across two time points. Results demonstrated that all the 18 *FLAs* were expressed in root tissues; especially, *PtrFLA2/12/20/21/24/30* were significantly induced at different time points. In summary, this study may lay the foundation for further investigating the biological functions of *FLA* genes in *Populus trichocarpa*.

## Introduction

Arabinogalactan proteins (AGPs) are heavily glycosylated proteoglycans that affect various processes such as plant growth, development, nutrition, reproduction, and adaptation to environmental changes (Showalter, 2001; Tan et al., 2012). AGPs are present in plant cells in large amounts (Majewska-Sawka and Nothnagel, 2000). Based on the protein structure, AGPs can be subdivided into six main categories: classical AGPs, Lys-rich AGPs, AG peptides, fasciclin-like AGPs (FLAs), non-classical AGPs, and chimeric AGPs (Schultz et al., 2002; Seifert and Roberts, 2007; Showalter et al., 2010). Fasciclin-like AGPs (FLAs) not only contain one or two AGP-like glycosylated regions, but also include one or two fasciclin domains. Most FLAs have a signal peptide with about 25 amino acid residues in N-terminal and a glycosylphosphatidylinositol (GPI) membrane anchor in hydrophobic C terminus. AGP-like glycosylated regions are rich in (Ser/Thr/Ala)-Hyp-(Ser/Thr/Ala)-Hyp and (Ser/Thr/Ala)-Hyp-Hyp repeat sequences. Classical AGPs contain more than 50% Pro (P), Ala (A), Ser (S), and Thr (T), but FLAs are composed of less than 35% PAST (Tan et al., 2003; Seifert and Roberts, 2007; Showalter et al., 2010). Fasciclin domains contain about 110–150 amino acid residues with low sequence similarity. However, a common denominator among fasciclin domains is that they all have two highly conserved regions (H1 and H2) and one [Phe/Tyr]-His motif, with approximately 10 amino acids in each conserved region (Kawamoto et al., 1998; Johnson et al., 2003; Huang et al., 2008). Fasciclin domains are first detected in fruit fly and have been discovered in various species, such as algae, lichens, plants, animal and humans, which mediate cell—cell and cell—extracellular matrix adhesion (Huber and Sumper, 1994). AGPs are mostly located in cellular membrane, cell walls and extracellular matrix, which may be involved in cell interactions, cell adhesion and cell wall biosynthesis (Kawamoto et al., 1998; Johnson et al., 2003; Shi et al., 2003).

With bioinformatics methods, the *FLAs* gene family has been identified and predicted in several plant genomes. 21 *FLAs* have been found in *Arabidopsis* (*Arabidopsis thaliana*), 24 in rice (*Oryza sativa*), 34 in wheat (*Triticum aestivum*), 19 in cotton (*Gossypium hirsutum*), 33 in Chinese cabbage (*Brassica rapa*), and 18 in eucalypt (*Eucalyptus grandis*) (Loopstra et al., 2000; Johnson et al., 2003; Lafarguette et al., 2004; Dahiya et al., 2006; Faik et al., 2006; Li and Wu, 2012; MacMillan et al., 2015). Nevertheless, only a few *FLAs* have been functionally characterized. For instance, *fla1* mutant has the ability to reduce shoot regeneration, indicating that *Arabidopsis AtFLA1* may be involved in lateral root initiation or emergence and shoot regeneration from root tissues (Johnson et al., 2011). Moreover, *AtFLA3* influences microspore development of *Arabidopsis*. Antisense suppression results in over 50% abnormal pollen grains, with the phenotypes of shrinking and wrinkling, which eventually lead to greatly decreased fertility. Further analysis demonstrates that plants overexpressing *AtFLA3* show short stamen filaments that cannot reach the stigma, fast growth of primary roots and abnormal root cap cells (Li et al., 2010). Additionally, analysis of salt overly sensitive 5 (*SOS5*) mutant suggests that *AtFLA4* may be related to cell abnormal expansion, cell adhesion, cell wall synthesis, and seed coat pectin mucilage (Shi et al., 2003). Several studies indicate that *AtFLA11* and *AtFLA12* are involved in tensile strength, biomechanics, and modulus of elasticity of stems, which may affect secondary cell wall composition and architecture (MacMillan et al., 2010). The latest findings also suggest that *PtFLA6* is associated with stem flexural strength, stiffness and biomechanics, and altered by cellulose and lignin composition in the xylem (Wang et al., 2015). In eucalypt, *EgrFLA2* and *EgrFLA3* are involved in cellulose microfibril angle and stem flexural strength, respectively (MacMillan et al., 2015). Overexpression of *GhFLA1* in transgenic cotton increases fiber length accompanied by up-regulation of other *FLA* genes associated with primary cell wall biosynthesis, whereas *GhFLA1* RNA interference plants have absolutely different effects (Huang et al., 2013). By *in situ* hybridization, maize *ZeFLA11* is specifically expressed in the differentiating xylem vessels (Dahiya et al., 2006). In addition to the above functions, FLAs may be involved in vascular formation and development, microfibril deposition orientation, cell wall thinness, cellulose deposition, cell wall matrix integrity, pollination, and embryogenesis (Majewska-Sawka and Nothnagel, 2000; Dahiya et al., 2006; MacMillan et al., 2010; Harpaz-Saad et al., 2011). Moreover, researchers are finding that *FLA* can respond to various biotic and abiotic stresses, such as salt stress, cold stress, drought stress, heat stress, and exogenous hormone ABA, pyrabactin and fluridone (Shi et al., 2003; Faik et al., 2006; Huang et al., 2013; Seifert et al., 2014). Soil salinity, which is a major abiotic stress that reduces plant productivity, affects large areas around the world (Toshio and Eduardo, 2005). In China, the total area of saline-alkali soil is approximately 8.11 × 10^7^ ha, accounting for 8–9% of total land area (Xu, 2004). These potential resistance genes are excellent candidates for genetic engineering to improve salt tolerance in agricultural and forestry plants.

In the whole life cycle of woody plants, there are distinct differences between primary and secondary growth stages, which may require a special molecular control system, but it remains unclear what role FLAs have played in woody plants. Therefore, this study may have a broad development prospect. *Populus*, which produces more valuable products such as wood pulp paper, furniture, and building materials, is one of the most important afforestation tree species in the whole world. Scientists have announced completion of the *P. trichocarpa* genome sequence that was rendered in 2006 (Tuskan et al., 2006), giving a chance to analyze and further understand *PtrFLAs*. In the present study, we identified 35 *FLAs* from the *P. trichocarpa* genome and conducted detailed analysis of the phylogeny, gene structure, conservation domain, gene expression patterns in different tissues, subcellular localization, as well as the response to salt stress. This research serves as a base for future studies and provides a fundamental clue for exploration into the functions of this gene family. In addition, identified genes presented here can be cloned in agricultural applications.

## Materials and methods

### Database searching and identification

In order to investigate the *FLA* genes family in *Populus*, all *Populus* proteins were downloaded from the *P. trichocarpa* genome database (http://www.phytozome.net). The Hidden Markov Model (HMM) profile of the fasciclin domain (PF02469) was downloaded from Pfam database (http://pfam.sanger.ac.uk/) for identification of the FLAs from the downloaded database of *Populus* proteins using HMMER3.0 software (Eddy, 1998). All potential FLA proteins were further screened to confirm the presence of the fasciclin domain (SM00554) by SMART (http://smart.embl-heidelberg.de/) (Letunic et al., 2012). The AGP-like glycosylated region referred to a protein sequence with two or more continuous (A/S/T) P or (A/S/T) PP motifs (excluding the fasciclin domains, N- and C-terminal signals). N-terminal signals peptide was predicted using SignalP 4.1 (Petersen et al., 2011). C-terminal GPI anchor addition signal was predicted using the big-PI Plant Predictor (http://mendel.imp.ac.at/sat/gpi/gpi_server.html) (Eisenhaber et al., 2003). Finally, a sequence harboring fasciclin domain, AGP-like glycosylated region, and N-terminal signal was considered as a *Populus FLA* gene. Protein subcellular localization was examined using Plant-mPLoc (http://www.csbio.sjtu.edu.cn/bioinf/plant-multi/). The ExPASy (http://web.expasy.org/compute_pi) was used to predict the *pI* and molecular weight.

### Phylogenetic and structural analyses

*Arabidopsis* AtFLA protein and wheat TaFLA protein sequences were obtained from NCBI protein database (http://www.ncbi.nlm.nih.gov/protein/). An neighbor-joining (NJ) phylogenetic tree of full-length sequences was constructed with 1000 bootstrap replicates implemented in the MEGA software (Tamura et al., 2007). Multiple sequence alignments corresponding to conserved motifs regions, characteristic of the PtrFLA protein members were determined by Clustal X with a gap open and the gap extension penalties of 10 and 0.1, respectively (Thompson et al., 1997).

### Gene structure and conserved motif analyses

Genomic sequences and open reading frames (ORFs) of *PtrFLAs* were obtained from Phytozome 10.2 (http://phytozome.jgi.doe.gov/pz/portal.html), and the exon/intron structure was identified with Gene Structure Display Server 2.0 (GSDS, http://gsds.cbi.pku.edu.cn/) (Hu et al., 2015). Conserved motifs of the genes were analyzed using the Multiple Em for Motif Elucidation (MEME) program (http://meme.nbcr.net/meme/) (Bailey and Elkan, 1994). MEME tool with the following parameters: the number of repetitions was set to zero or one; optimum motif width was set to 30–70; the maximum number of motifs was set to identify 20 motifs.

### Chromosome localization

All information about the chromosomal position of the genes was obtained from the *Populus* Genome Browser (http://www.phytozome.net/poplar). A schematic diagram of their locations on the chromosomes was drawn using MapInspect software (Zhao et al., 2011).

### Estimation of *K*a/*K*s ratios and duplication analyses

Protein sequences of the gene pairs were multiply aligned using Clustal X program. Then, pair-wise multiple alignment of proteins and the corresponding mRNA was converted into codon alignment using PAL2NAL (http://www.bork.embl.de/pal2nal/). Non-synonymous substitution (*Ka*) and synonymous substitution (*Ks*) values were automatically calculated on PAL2NAL website by using the codeml program in PAML (Suyama et al., 2006). According to molecular clock hypothesis, a species of duplicate genes of synonymous substitution rate is constant. The divergence times (*T*) can be evaluated according to the following formula: *T* = *Ks*/2λ. Where *T* was measured in years; λ was variable among different species. For *Populus*, λ = 9.1 × 10^−9^ (Lynch and Conery, 2000).

### Preparation of plant materials

*Populus* (*P. simonii* × *P. nigra* cross, 18-year-old) was obtained from Northeast Forestry University in Heilongjiang Province, China. One-year-old *P. trichocarpa* was planted in the greenhouse under controlled conditions with a relative humidity of 60–75% at (22 ± 2)°C under a photosynthetic photon flux density of 200 μmol m^−2^ s^−1^ using cool white fluorescent lights. For detecting the expression level in different tissues, male and female inflorescences of *Populus* (*P. simonii* × *P. nigra* cross) were sampled in April 2014. Leaf buds, apex shoots, young stems, differentiating xylem, young leaves, and roots were gained from stems of *Populus* (*P. trichocarpa*) hydroponic cuttings in April 2014. For determination of salt response, twigs of *P. trichocarpa* were planted in pots under normal water conditions in the greenhouse. One-month-old seedlings (30–40 cm in height) were treated with 150 mM NaCl for 0, 12, and 24 h, respectively. The first time point (0 h) served as a control. After NaCl treatment, young roots were harvested from five seedlings. All the samples were immediately frozen in liquid nitrogen, and stored at −80°C before total RNA isolation.

### RNA extraction and qRT-PCR

Total RNA was isolated with RNA extraction kit (TaKaRa, Dalian, China) from *Populus* samples (Zheng et al., 2014). The purity and quality of RNA was analyzed by NanoDrop 2000c (Thermo-Scientific, USA). A 1 μg aliquot of total RNA was treated with DNase I (Invitrogen, Carlsbad, CA, USA) and reverse-transcribed using the PrimeScript RT reagent kit (TaKaRa). Two Microliter of cDNA template (equivalent to 100 ng of total RNA) was used in qRT-PCR with SYBR Premix EX *Taq* II (TaKaRa) and the MJ Opticon 2 System (Bio-Rad, USA) according to the manufacturer's instructions. The gene-specific primers were used to quantify the transcripts of *FLAs* with *Ptractin* genes as internal references. All the primers used in the present study for qRT-PCR were listed in Supplemental Table 6. Each reaction was conducted in triplicate to ensure reproducibility of results. Expression levels were calculated from the cycle threshold according to the delta-delta CT method (Livak and Schmittgen, 2001). The statistical significance between mean values across different tissues was determined by one-way analysis of variance. Tukey's *post-hoc* test was performed to identify significant differences in different tissues if the analysis of variance was significant.

### Microarray data analyses

Tissue-specific expression data were retrieved from the Gene Expression Omnibus (GEO, http://www.ncbi.nlm.nih.gov/geo/) (GEO accession number: GSE56023). Probe sets corresponding to *PtrFLA* genes were searched using an online Probe Match tool available at NetAffx Analysis Center (http://www.affymetrix.com/). The same probe set matched to several genes indicates the same level of transcript abundance. The heat maps were drawn using the HemI (Heat map illustrator) with the default value (Deng et al., 2014).

### Subcellular localization

For subcellular localization analysis, the full-length *PtrFLA2/20/26* coding regions (without the stop codon) were amplified from cDNA of *P. trichocarpa* differentiating xylem by PCR. The primers used to amplify *PtrFLA2/20/26* were listed in Supplemental Table 6. The PCR products were digested with *Sal* I and *Nco* I, and directionally ligated into vector pTH2 to construct the *PtrFLA2/20/26*-*GFP* fusion gene driven by a *CaMV 35S* promoter (Niwa, 2003). The differentiating xylem (wood-forming tissue) were collected in a large quantity from the stem of a 6-month-old *P. trichocarpa* plant after debarking. To isolate the stem-differentiating xylem (SDX) cells, the debarked stem was soaked in cell wall digestion solution. The isolation and transfection of protoplasts from SDX was performed as described in Nature Protocols (Lin et al., 2014). The transient expression of the PtrFLA2/20/26-GFP fusion proteins was observed using Zeiss Confocal Microscopy (model LSM410, Zeiss, Jena, Germany).

## Results

### Identification of putative *PtrFLAs*

*FLA*, containing fasciclin domains and AGP-like glycosylated regions, has been previously comprehensively analyzed in *Arabidopsis*, rice and wheat. To isolate putative *PtrFLA* genes in the *Populus* genome, we searched the latest version (3.0) of databases from Phytozome 10.2 (http://www.phytozome.net). We applied the Hidden Markov Model (HMM) profile of the fasciclin domain (PF02469) with HMMER3.0 software to search the protein sequences of *P. trichocarpa* and then validated the complete fasciclin domains (SM00554) using SMART software with an *E* < 1.0. *PtrFLA* candidates containing both the full-length coding sequence (CDS) and N-terminal signal peptide (predicted by SignalP 4.1) were further screened. Subsequently, AGP-like glycosylated regions were manually scanned in the remaining sequences. The general principle is that there are at least two non-contiguous Hyp residues such as (Ser/Thr/Ala)-Hyp-(Ser/Thr/Ala)-Hyp and (Ser/Thr/Ala)-Hyp-Hyp (Schultz et al., 2002; Faik et al., 2006). Ultimately, 35 *PtrFLA* non-redundant genes were identified in the *Populus* genome and named as *PtrFLA1*-*PtrFLA35* based on their chromosomal positions from top to bottom successively (Supplemental Tables 1, 2). The proportion of Pro, Ala, Ser, and Thr (PAST) residues was 26.57–42.59% (Supplemental Table 3) on the basis of statistical method (Schultz et al., 2004). 23 C-terminal GPI anchor proteins were predicted using big-PI Predictor software, accounting for about 65.71%. The length of PtrFLAs ranged from 227 to 466 amino acids (aa) with an average of 306 aa and the isoelectric points ranged between 4.38 and 10. 8 (Table 1).

**Table 1:** *****FLA*** genes in ***Populus trichocarpa*****.

**Gene symbol**	**Gene locus**	**Genomic position**	**Gene length (bp)**	**ORF (bp)**	**Protein length (aa)**	**Mol.Wt (Da)**	**pI**	**Localization**
*PtrFLA1*	Potri.001G320800	Chr01: 32566530–32567917	1388	732	243	26072.9	7.99	Membrane
*PtrFLA2*	Potri.001G367900	Chr01: 38081667–38084340	2674	1221	406	43115.3	5.62	Membrane
*PtrFLA3*	Potri.002G223300	Chr02: 21076590–21078318	1729	792	263	27722.3	4.65	Membrane
*PtrFLA4*	Potri.004G210600	Chr04: 21827522–21828762	1241	807	268	28250.5	8.89	Membrane
*PtrFLA5*	Potri.005G079500	Chr05:5852241-5853569	1329	1329	442	47582.5	5.95	Membrane
*PtrFLA6*	Potri.006G129200	Chr06: 10546378–10547472	1095	720	239	25127.5	5.27	Membrane
*PtrFLA7*	Potri.006G200300	Chr06: 21552110–21555071	2962	1401	466	50976	6.16	Mem, chlo
*PtrFLA8*	Potri.008G012400	Chr08: 685882–690857	4976	1392	463	51037	6.06	Membrane
*PtrFLA9*	Potri.009G012100	Chr09: 2101448–2102467	1020	792	263	28752.2	8.77	Membrane
*PtrFLA10*	Potri.009G012200	Chr09: 2104068–2105349	1282	810	269	28351.4	7.74	Membrane
*PtrFLA11*	Potri.010G244900	Chr10: 21994487–21999392	4906	1380	459	50838.8	5.95	Membrane
*PtrFLA12*	Potri.011G093500	Chr11: 11364228–11367418	3191	1227	408	43721	5.58	Membrane
*PtrFLA13*	Potri.012G015000	Chr12: 1466939–1471579	4641	810	269	28147.6	7.80	Membrane
*PtrFLA14*	Potri.012G127900	Chr12: 14557761–14560026	2266	723	240	25352.7	5.33	Membrane
*PtrFLA15*	Potri.013G014200	Chr13: 912614–913708	1095	801	266	28178	7.02	Membrane
*PtrFLA16*	Potri.013G120600	Chr13: 13393705–13394592	888	717	238	24798.2	6.55	Membrane
*PtrFLA17*	Potri.013G151300	Chr13: 15545458–15546340	883	810	269	28400.3	6.18	Membrane
*PtrFLA18*	Potri.013G151400	Chr13: 15548757–15553715	4959	810	269	28340.2	7.06	Membrane
*PtrFLA19*	Potri.013G151500	Chr13: 15559778–15560942	1165	795	264	27569.4	7.91	Membrane
*PtrFLA20*	Potri.014G071700	Chr14: 5793739–5795660	1922	1266	421	43250.3	5.37	Mem, Chlo
*PtrFLA21*	Potri.014G162900	Chr14: 12850627–12852961	2335	789	262	27635.3	5.63	Membrane
*PtrFLA22*	Potri.014G168100	Chr14: 13433009–13434622	1614	1194	397	42646.6	5.64	Membrane
*PtrFLA23*	Potri.015G013300	Chr15: 869556–870642	1087	804	267	28234.8	7.80	Membrane
*PtrFLA24*	Potri.015G129400	Chr15: 14100586–14102248	1663	723	240	25389.9	6.72	Membrane
*PtrFLA25*	Potri.016G066500	Chr16: 4713550–4716897	3348	1401	466	51132.1	6.06	Mem, chlo
*PtrFLA26*	Potri.016G088700	Chr16: 7097575–7099033	1459	720	239	25188.5	5.73	Membrane
*PtrFLA27*	Potri.017G111600	Chr17: 12724860–12725918	1059	1059	352	38260.7	4.38	Membrane
*PtrFLA28*	Potri.019G002300	Chr19: 281955–282806	852	852	283	30637.6	10.8	Membrane
*PtrFLA29*	Potri.019G049600	Chr19:7413805-7414563	759	759	252	27188.4	6.28	Membrane
*PtrFLA30*	Potri.019G093300	Chr19: 12352008–12352993	986	738	245	25481.9	6.72	Membrane
*PtrFLA31*	Potri.019G120900	Chr19: 14875495–14876429	935	684	227	23832.3	6.96	Membrane
*PtrFLA32*	Potri.019G121100	Chr19: 14892331–14893290	960	789	262	27526.4	6.50	Membrane
*PtrFLA33*	Potri.019G121200	Chr19: 14900852–14901643	792	792	263	27479.3	6.28	Membrane
*PtrFLA34*	Potri.019G122800	Chr19: 15107258–15108050	793	759	252	26545.3	6.64	Membrane
*PtrFLA35*	Potri.019G123200	Chr19: 15135778–15137005	1228	792	263	27583.5	6.97	Membrane

### Phylogenetic analysis of PtrFLAs

**Figure 1:**
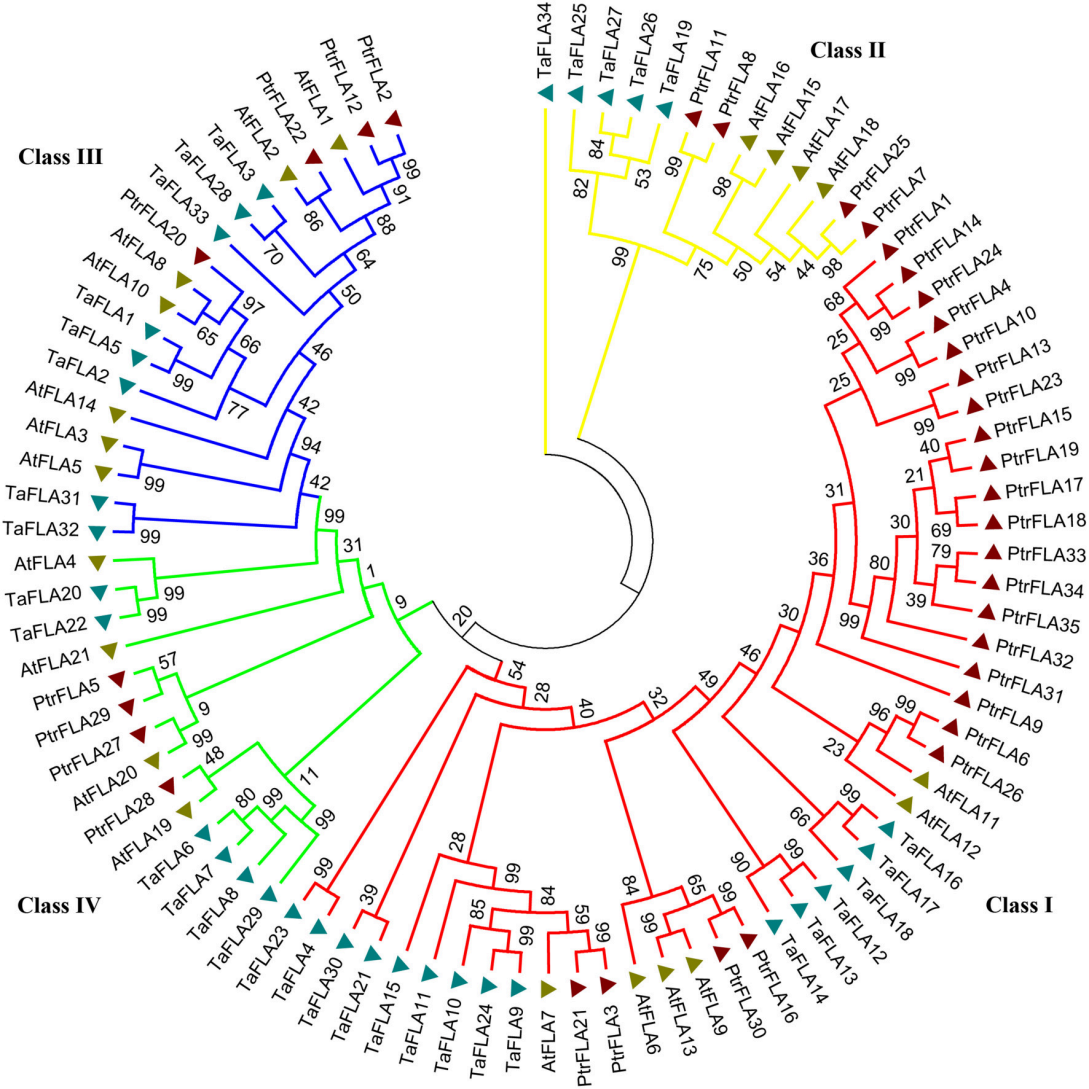
**The phylogenetic tree of FLA proteins from ***Populus***, wheat and ***Arabidopsis*****. Totally 35 FLA proteins in *Populus*, 34 in wheat and 21 in *Arabidopsis* were analyzed using Clustal X 2.0 and the Neighbor-Joining tree was constructed using MEGA 5.0.5. The Bootstrap value was 1000 replicates.

To investigate the evolutionary relationships between and among *Populus, Arabidopsis*, and *Triticum*, we constructed an neighbor-joining (NJ) phylogenetic tree of 35 *Populus* FLA proteins, 21 *Arabidopsis* FLA proteins, and 34 *Triticum* FLA proteins, with 1000 bootstrap replicates (Figure 1). The phylogenetic distribution revealed that FLA proteins were divided into four major classes: class I, II, III, and IV. Class I contained most of the members, which was about the sum of members in the remaining three classes. AtFLA11/12/PtrFLA6/26, AtF LA6/9/13/PtrFLA16/30, and AtFLA20/PtrFLA5/27/29 were clustered into three individual clades without wheat FLA proteins, suggesting that these clades were dicotyledon-specific. In addition, AtFLA4/TaFLA20/22 represented a novel clade and might have independent evolutionary trajectory compared with others clades. Moreover, several genes from the same species were distinguished from other FLA proteins as they formed clades, which appeared to be specific to these three species, such as AtFLA3/5, TaFLA31/32, PtrFLA1/4/9/10/13/14/15/17/18/19/23/24/31/32/33/ 34/35. Finally, three pair of orthologous genes from *Arabidopsis* and *Populus* were identified, including AtFLA2/PtrFLA22, AtFLA19/PtrFLA28, and AtFLA20/Ptr FLA27.

**Figure 2:**
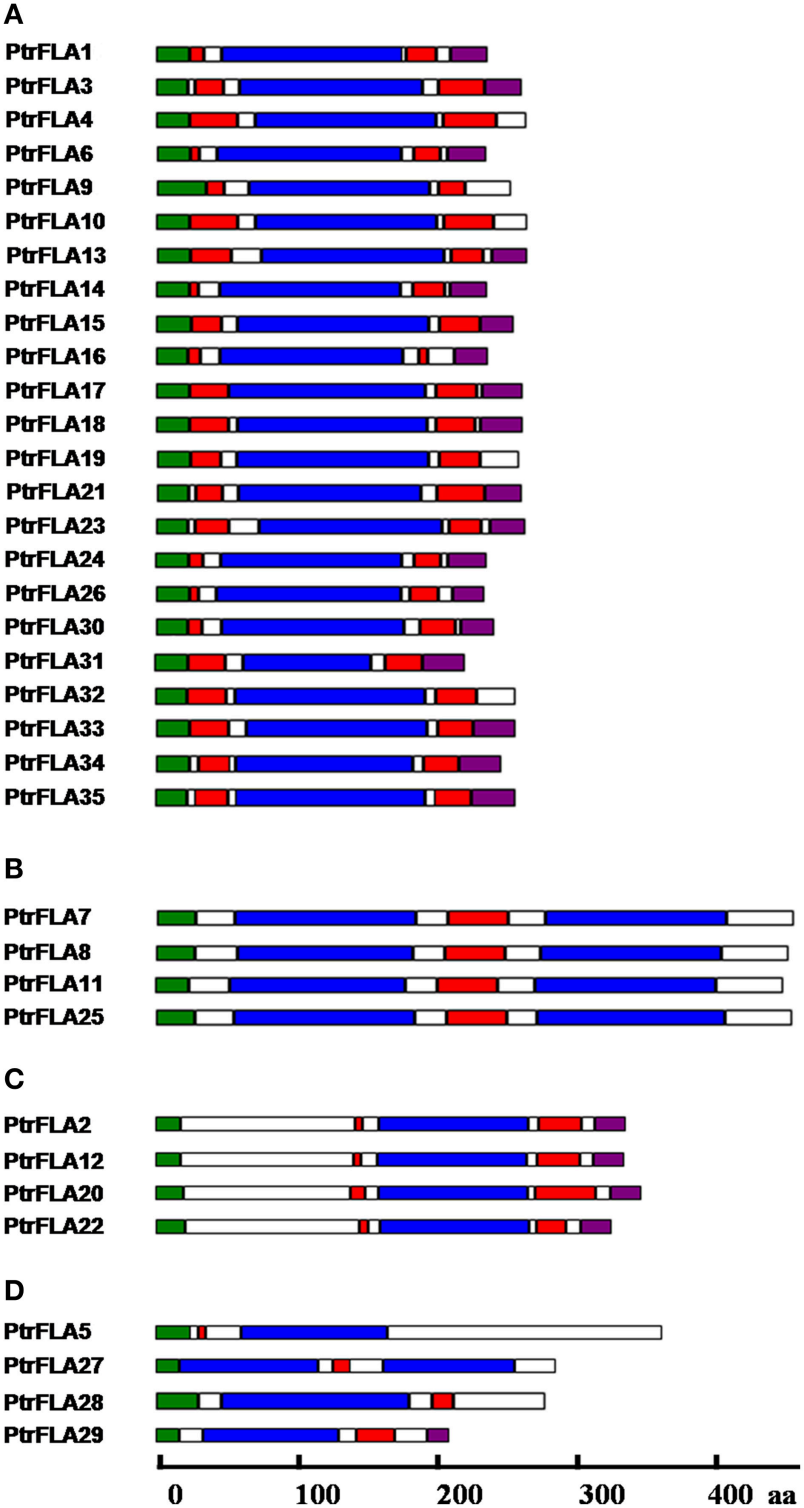
**Schematic representation of PtrFLAs based on the numbers of AGP-like glycosylated regions and fasciclin domains**. **(A–D)** Represent four different groups of PtrFLAs. The colored boxes indicate the N-terminal peptide signal (green), fasciclin domain (blue), AGP glycosylated region (red), and C-GPI anchor signal (purple), respectively.

The *Populus* FLA family was classified into groups A-D based on Johnson et al.'s group classification (Johnson et al., 2003; Figure 2). Group A was the largest group with 23 members, containing a single fasciclin domain that was flanked by two AGP-like glycosylated regions, meanwhile 5 of the 23 members lacked the site of C-terminal GPI anchor. Group B included 4 members (PtrFLA7/8/11/25) that contained two fasciclin domains flanking one AGP-like glycosylated region, but none contained the site of C-terminal GPI anchor. Group C also comprised of 4 members (PtrFLA2/12/20/22) that contained a C-terminal GPI anchor site and two AGP-like glycosylated regions flanking one fasciclin domain. Groups A and C had similar structure, but they were not clustered into the same group because there was a big disparity between the location of domain and sequence length. The final group D also included 4 members (PtrFLA5/27/28/29) that had no obvious correlation with each other or with other FLAs. Except for PtrFLA27, the remaining three had one fasciclin domain and one AGP-like glycosylated region, among which only *PtrFLA29* contained C-terminal GPI anchor site. *PtrFLA27* had a similar structure with group B and contained two fasciclin domains flanking one AGP-like glycosylated region, but it was not clustered into group B because the location of domains and the length of protein sequence were considerably different.

### Multiple sequence alignment of fasciclin domains of PtrFLAs

**Figure 3:**
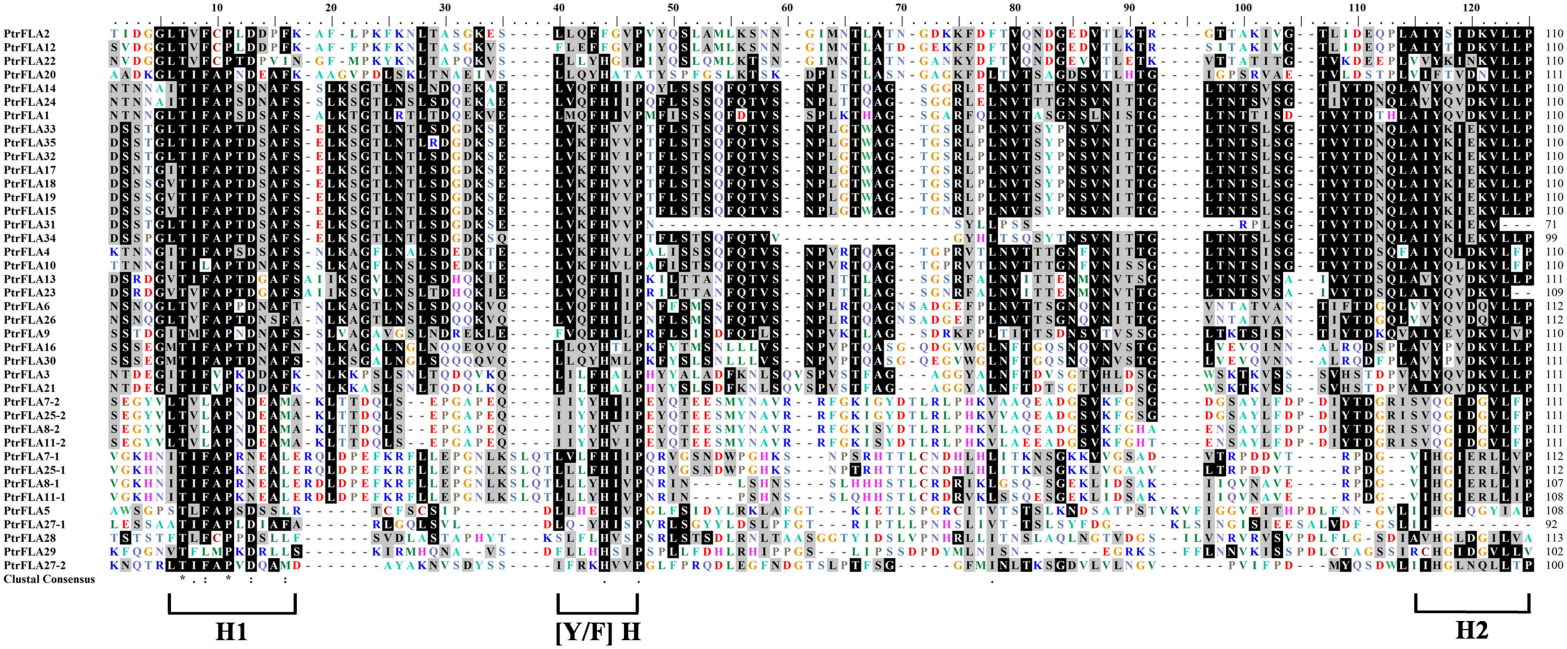
**Multiple sequence alignment of fasciclin domains in ***Populus trichocarpa*****. The alignment was generated using Clustal W program and manual modification. If a protein sequence contains two fasciclin domains, numbers 1 and 2 were used to distinguish them based on the distance to N-terminus. The conserved regions (H1, H2, and [Y/F]H) of fasciclin domains are indicated in the bottom. Identical and conserved residues are shaded in black and dark gray, respectively.

Multiple sequence alignment of fasciclin domains from *Populus* was conducted using Clustal X software, which contained highly conserved regions, H1 and H2 (with the length of about 10 amino acids for each). [Tyr/Phe]-His ([Y/F] H) motif was located between them according to Pfam database (Figure 3). The Thr residue in the H1 region was absolutely conserved in the fasciclin domain from *Populus*, just as that from *Arabidopsis*, wheat, rice, and cotton (Johnson et al., 2003; Faik et al., 2006; Huang et al., 2008). Furthermore, a majority of Asp and a small quantity of Asn residues generally occupied the sixth position after the Thr residue. In addition, the remainder of H1 region was basically composed of Ala, Ile, Phe, Pro, and Ser. Similarly, most of PtrFLAs also included other conserved residues, such as Leu, Lys, Ser, and Gly, which were near the H1 region and might play a role in maintaining the structure of fasciclin domains and cell adhesion (Johnson et al., 2003). C-terminal fragments of H2 regions were abundant in small hydrophobic amino acids, such as Val, Leu, and Ile in all known fasciclin domains (Shi et al., 2003; Lafarguette et al., 2004). The other parts of H2 regions had a relatively conservative characteristic. In the [Y/F] H motif, His residue was substituted by only two Phe residues in PtrFLA2 and PtrFLA12, Glu, Lys, Leu and His instead of Tyr or Phe residues such as PtrFLA5, PtrFLA27-2, PtrFLA28, and PtrFLA29. Interestingly, compared with any other species, Pro residue was absolutely or extremely conserved in these three regions.

### Sequencing and structural analysis of *PtrFLAs*

**Figure 4:**
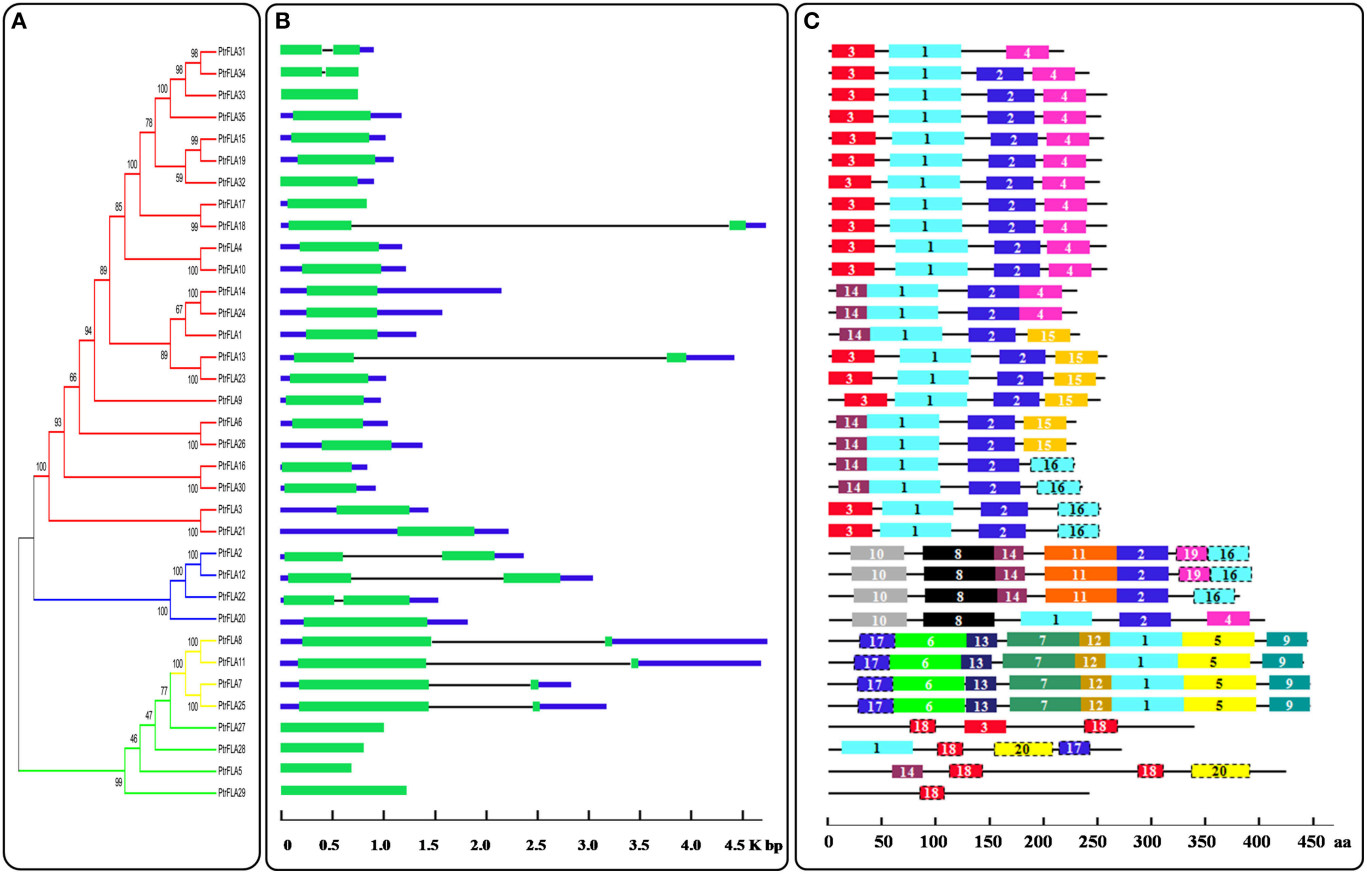
**Phylogenetic relationships, gene structure and motif compositions of PtrFLAs. (A)** The full-length PtrFLA protein sequences were aligned by Clustal X 2.0 and the un-rooted tree was generated with the MEGA5.0.5 by Neighbor-Joining (NJ) method with 1000 bootstrap replicates. The branches of different groups were painted with different colors. **(B)** Exon/intron structures of the *PtrFLAs*. Green boxes represent exons and black lines represent introns. **(C)** Conserved motif analysis of PtrFLAs by MEME. Different motifs are represented by different colored boxes with number 1–20.

To thoroughly reveal the structural diversity of **PtrFLA** genes, we constructed the intron/exon arrangements and conserved motifs based on the phylogenetic tree (Figure 4A). First of all, the coding sequence of each *PtrFLA* gene was compared with own genomic sequence, respectively (Figure 4B). Overall, some closest genes showed similar structures, which only differed in the length of intron and exon, but a small proportion of gene pairs exhibited different intron/exon arrangements. For instance, *PtrFLA17*/*PtrFLA23* contained only one exon, whereas their nearby paralogous genes *PtrFLA18/PtrFLA13* had two exons and one intron, even though their evolutionary relationships reached 99 and 100% bootstrap value respectively. Finally, all the members of the groups B and D were consistent with the number of exons, respectively. In brief, *PtrFLAs* have two structures: (1) harboring only one exon; (2) harboring one intron and two exons.

To further reveal the specific regions of PtrFLA proteins, the conserved motifs were predicted by MEME and 20 individual motifs were isolated (Figure 4C, Supplemental Table 4). The length of motifs ranged from 30 to 70 amino acids and the number of motifs varied between 1 and 8 in each PtrFLA protein. The fundamental principle was that the motif composition of peer group was characterized by the same or similar structure; especially, members of group B exhibited absolutely identical motifs, which suggested functional similarities among the PtrFLA proteins within the same group. Different groups contained distinct conserved motifs. For instance, motifs 1 and 2 appeared in nearly all members of group A. Motif 5, 6, 7, 9, 12, 13 were conserved in group B. Motif 8, 10, 11, 19 were specific to group C. Motif 18 and 20 existed only in group D. Motif 1, 2, 3 were commonly shared by most of the PtrFLA family members, which were part of the fasciclin-like domains. The results indicated that these conserved motifs may play critical roles in specific functions or show similar functionality. Although the functions of some motifs were not yet clear, the presence of these conserved motifs certainly reflected functional similarities among PtrFLAs.

### Chromosomal localization and gene duplication

**Figure 5:**
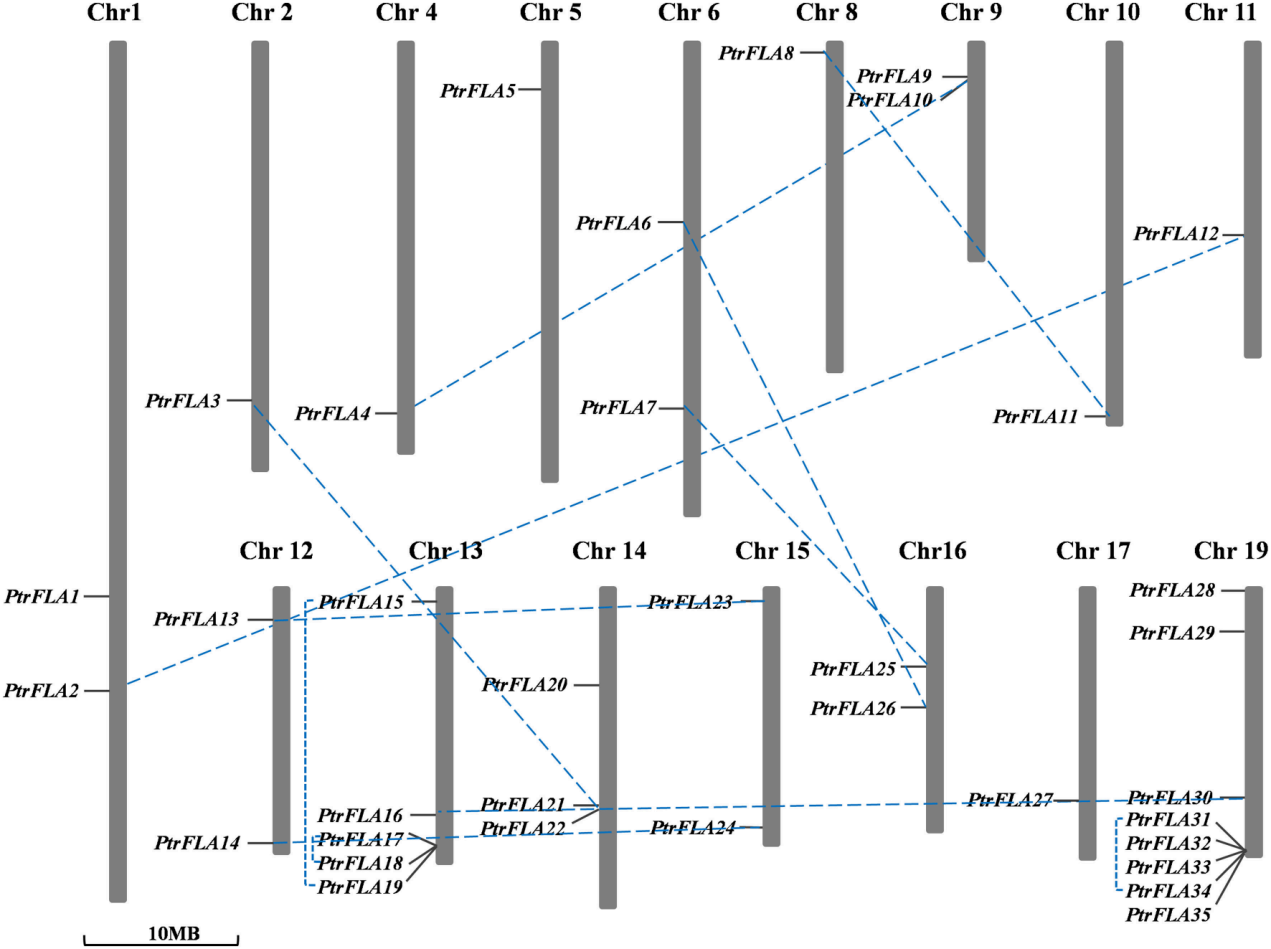
**Chromosomal locations and duplicated gene pairs of ***PtrFLAs*****. The 35 *PtrFLA* genes were mapped to the 16 chromosomes. Three chromosomes (chr3, 7, and 18) have no *PtrFLA* gene. The chromosome number is indicated at the top. The duplicated gene pairs are connected with dotted blue line.

The genomic localization of each *PtrFLA* gene was marked on the chromosomal maps (Figure 5). The 35 *PtrFLAs* were randomly and unevenly distributed on 16 chromosomes but absent on chromosome 3, 7, and 18. There were eight *PtrFLA* genes on chromosome 19 which was the maximum number of genes, followed by chromosome 13 (5 genes), whereas chromosome 14 had three genes, and only one *PtrFLA* gene and two genes were distributed on chromosome 2/4/5/8/10/11/17 and chromosome 1/6/9/12/15/16, respectively.

Gene duplication events can be divided into three categories: interspersed repeat, tandem repeat, segmental duplication, resulting in gene functional diversity, family evolution and plant adaptations (Kong et al., 2007). We calculated the *Ka/Ks* ratio to explore the trend of gene divergence after duplication. Generally, *Ka/Ks* = 1 means neutral selection; *Ka/Ks* < 1 means purifying selection; *Ka/Ks* > 1 means accelerated evolution with positive selection (Hurst, 2002). We calculated 12 gene pairs based on phylogenetic analysis and chromosomal distribution analysis of *FLAs* (Table 2). Specifically, 9 gene pairs were randomly distributed on different chromosomes, and the remainder of 3 gene pairs was located on the same chromosome. Therefore, in *FLA* genes, segmental duplication played a leading role in gene duplication and the function of genes did not obviously change during the long process of evolution. Nevertheless, any forms mentioned above were beneficial to the expansion and the richness of *PtrFLA* genes. The *Ka/Ks* ratios of all the gene pairs were less than 0.5, suggesting that functional divergence might occur before duplication events. The results indicated that purifying selection was far superior to positive selection in the evolution of *PtrFLA* genes.

**Table 2:** **Estimated divergence period of ***PtrFLA*** gene pairs in ***Populus trichocarpa*****.

**Gene pairs**	**Ka**	**Ks**	**Ka/Ks**	**MYA**
PtrFLA2 vs. PtrFLA12	0.0600	0.2364	0.2539	13.0
PtrFLA3 vs. PtrFLA21	0.0685	0.2571	0.2665	14.1
PtrFLA4 vs. PtrFLA10	0.0845	0.3179	0.2658	17.5
PtrFLA6 vs. PtrFLA26	0.0357	0.2945	0.1211	16.2
PtrFLA7 vs. PtrFLA25	0.0225	0.3196	0.0705	17.5
PtrFLA8 vs. PtrFLA11	0.0263	0.2573	0.1022	14.1
PtrFLA13 vs. PtrFLA23	0.0733	0.3306	0.2216	18.2
PtrFLA14 vs. PtrFLA24	0.0387	0.2409	0.1605	13.2
PtrFLA15 vs. PtrFLA19	0.0276	0.0909	0.3036	5.0
PtrFLA16 vs. PtrFLA30	0.0722	0.3050	0.2369	16.8
PtrFLA17 vs. PtrFLA18	0.0396	0.1197	0.3311	6.6
PtrFLA31 vs. PtrFLA34	0.0428	0.0858	0.4991	4.7

We also calculated the time of the duplication events based on synonymous substitution rate *Ks*. The results showed that segmental duplications of *PtrFLA* genes originated from 13.0 MYA (*Ks* = 0.2364) to 18.2 MYA (*Ks* = 0.3306); tandem repeats were from 4.7 MYA (*Ks* = 0.0858) to 6.6 MYA (*Ks* = 0.1197). The newly duplicated genes were more likely to adapt to extreme environments and maintain existing functions.

### Organ-specific expression of *PtrFLA* genes

**Figure 6:**
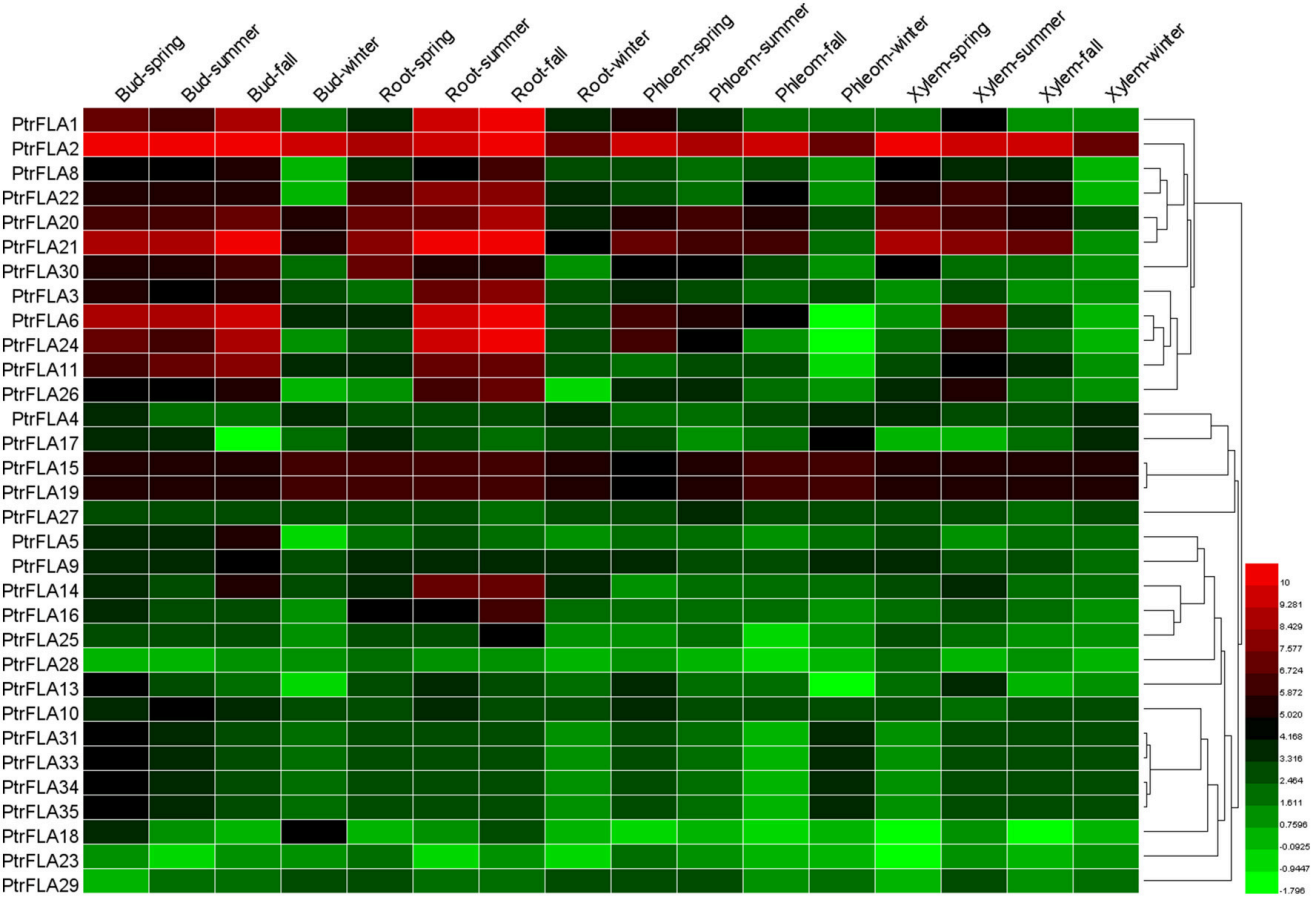
**Hierarchical clustering of expression profiles of 32 ***PtrFLA***s in different tissues at four seasons**. The data was obtained from GEO microarry database (GSE56023). Details of the data are shown in Supplemental Table 7. Color scale represents log2 rate expression values.

The expression patterns of genes can provide useful clues for further investigation of the functions of these genes. To verify the expression profiles of *PtrFLAs* genes, the microarray data were obtained from GEO (GSE56023) of four *Populus* vegetative tissues (buds, roots, xylem and phloem) at four seasons. 32 of 35 genes were found in the microarray data (Supplemental Table 5). 32 *PtrFLAs* genes were ubiquitously expressed in almost all detected tissues (Figure 6, Supplemental Table 7). The expression levels of *PtrFLA2/15/19* were highly similar in all detected tissues, with slight changes among different seasons. The expression levels of *PtrFLA1/6/8/11/14/20/21/22/24/25/26/30* firstly increased as the seasons changed and then decreased in winter. In addition, *PtrFLA2/6/8/11/15/19/20/21/24* were highly expressed in apical meristems (buds and roots) and vascular bundles (phloem and xylem). Furthermore, the expression levels of other *PtrFLAs* varied with an unclear variation curve.

**Figure 7:**
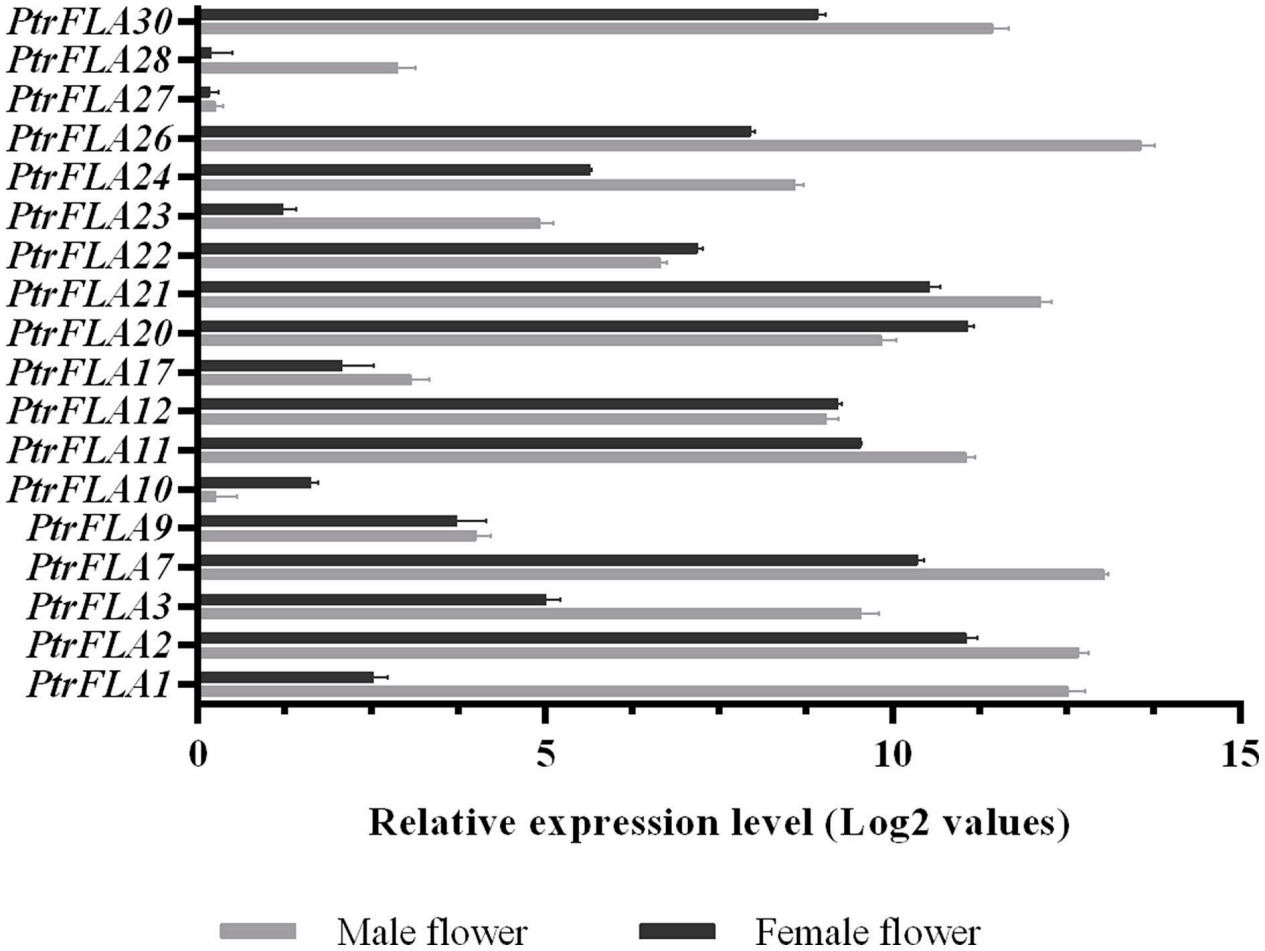
**Expression analysis of 18 selected ***PtrFLA*** genes in male inflorescences and female inflorescences by qRT- PCR**. The expression level of each gene was calculated relatively to the transcript lowest expressed in male or female inflorescences. Error bars represent standard error for three replicates.

**Figure 8:**
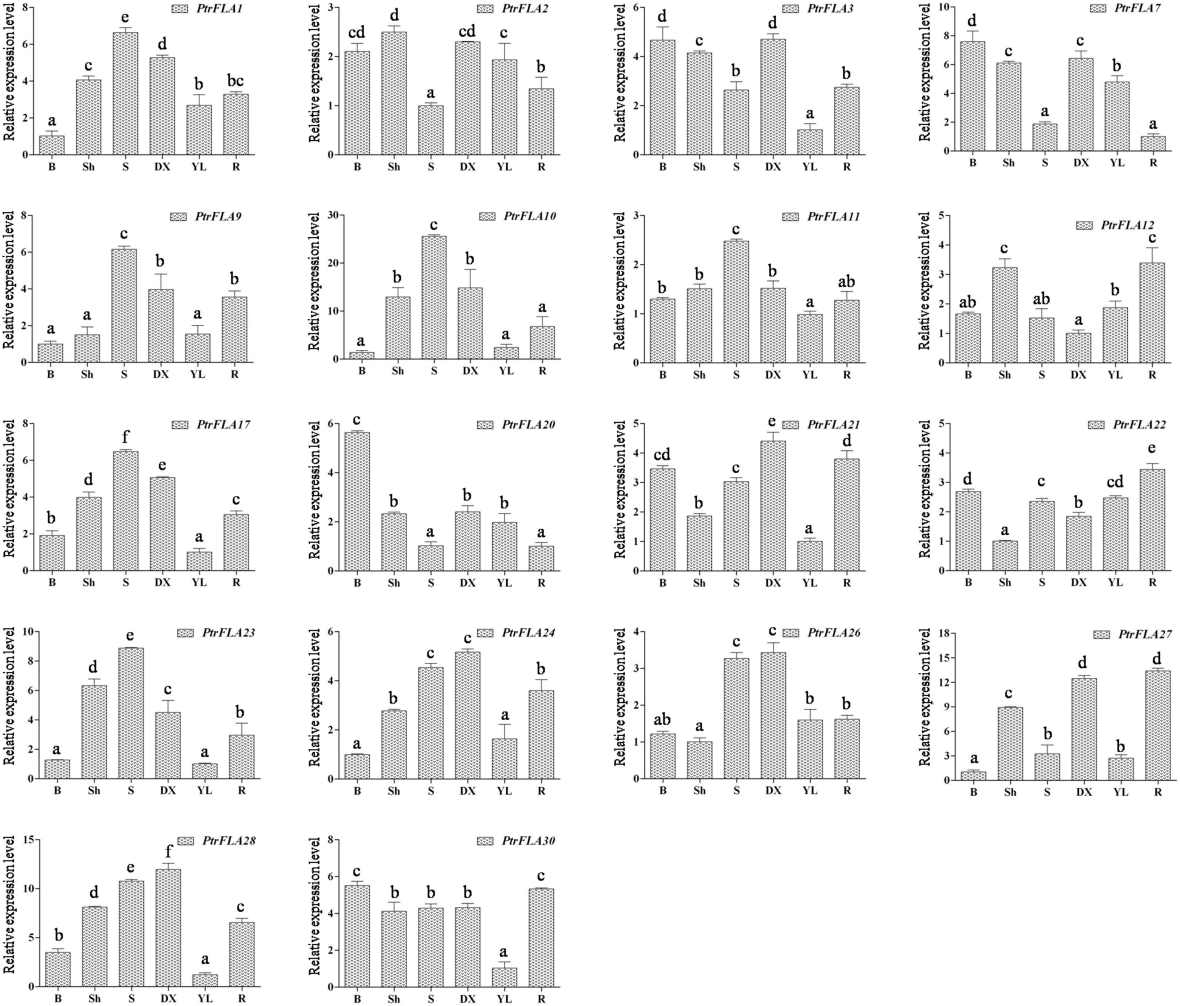
**Expression analysis of 18 selected ***PtrFLA*** genes in different vegetative tissues by qRT- PCR**. B, leaf buds; Sh, apex shoots; S, stems; DX, differentiating xylem; YL, young leaves; R, roots. The expression level of each gene was calculated relatively to the average biological replicate of sample which was expressed at lowest level. Different letters indicate statistically significant differences when analyzed by One-way ANOVA and a multiple comparison using Tukey' s test at *P* ≤ 0.05. Data represent nine individual *Ct* values of each sample. Error bars represent standard error for three replicates.

In order to verify and enrich the expression profiles of *PtrFLAs* genes obtained by microarray, qRT-PCR analysis of 18 selected *PtrFLA* genes in eight different tissues (flower buds, leaf buds, apex shoots, stems, differentiating xylem, leaves and roots) was performed to analyze the expression levels of *PtrFLAs* genes. The gene expression pattern detected by qRT-PCR was generally consistent with the microarray results. In the reproductive organs, all the selected *PtrFLAs* genes were expressed in male and female inflorescences (Figure 7). In detail, only the expression levels of *PtrFLA10/22* in male inflorescences were lower than that in female inflorescences; the expression levels of *PtrFLA9/12/27* showed no significant difference between male inflorescences and female inflorescences; the expression levels of other *PtrFLA1/2/3/7/11/17/20/21/23/24/26/28/30* in the male inflorescences were higher than that in female inflorescences; especially, the expression levels of *PtrFLA1/3/26* in different inflorescences differed by more than 20-fold. In other vegetative tissues, *PtrFLA1/9/10/11/17/21/24/26/28* were highly expressed in the stems and differentiating xylem (Figure 8). Interestingly, *PtrFLA2/7/20/27* were highly expressed either in the stems or differentiating xylem. In other meristems, *PtrFLA12/21/22/24/27/28/30* were highly expressed in the roots, *PtrFLA2/3/7/12* were highly expressed in the apex shoots, while *PtrFLA2/3/7/20/21/22/30* were highly expressed in the leaf buds. In the young leaves, only *PtrFLA2/7* exhibited a high expression level. Moreover, except the young leaves, the expression levels of *PtrFLA11/30* showed slight expression differences among the selected samples.

### Subcellular localization of PtrFLAs proteins

**Figure 9:**
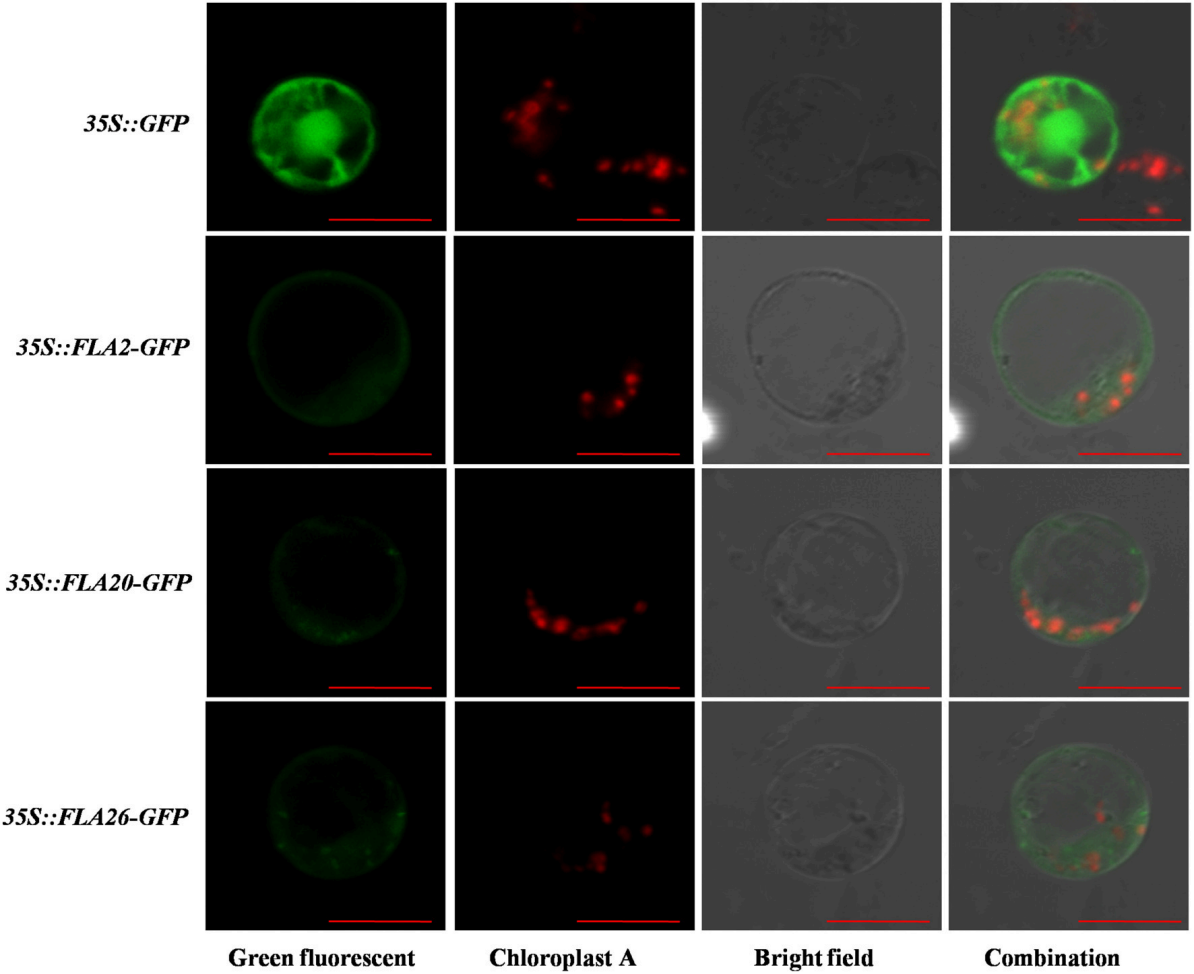
**Subcellular localization of PtrFLA2/20/26**. The photographs were taken under dark-field illumination for green fluorescence localization (Green fluorescent), under the red fluorescence illumination to examine chloroplast auto-fluorescence localization (Chloroplast A), under bright-field illumination to examine cell morphology (Bright field), and under combination-field illumination (Combination). The bar was 10 μm.

*In silico* analyses using the protein subcellular localization prediction software Plant-mPLoc enabled us to predict the possible localization of each of the different candidate PtrFLAs in *Populus*. Almost all the PtrFLAs were predicted to be localized in the membrane with high reliability, while PtrFLA7/25 were predicted to be localized in membrane and chloroplasts (Table 1). Furthermore, some genes may be also localized in other cytoplasmic matrix and organelles, such as mitochondria, endoplasmic reticulum, nucleus, and vacuole, but the reliability is very low. To confirm the predicted localizations, some of these proteins were transiently expressed in protoplasts from *Populus* stem-differentiating xylem (SDX) as fusions with the N-terminus of GFP. Finally, three PtrFLA proteins were successfully expressed as fluorescent protein fusions (35S::FLA2-GFP, 35S::FLA20-GFP, and 35S::FLA26-GFP). As shown in Figure 9, the fluorescent signal of 35S::GFP control was detected both in the nucleus and cytoplasm. The 35S::FLA2-GFP fluorescence in transformed protoplasts was observed in cell membrane and some cytoplasm. The 35S::FLA20/26-GFP fluorescence in transformed protoplasts was also observed in cell membrane and cytoplasm, but there were some brightened dots around the cells (Figure 9).

### Expression analysis of *PtrFLA* genes under salt stress

**Figure 10:**
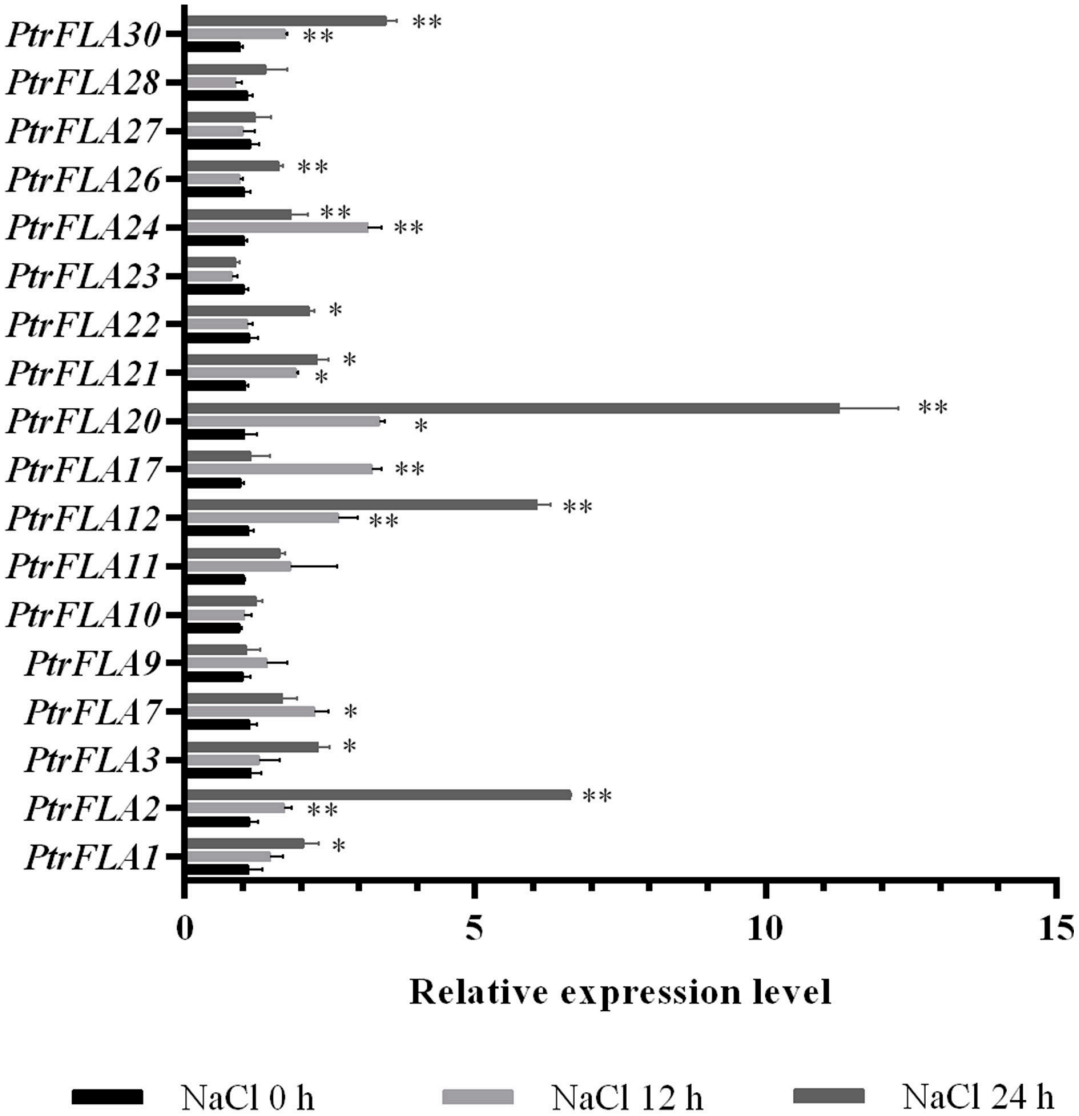
**Expression analysis of 18 selected ***PtrFLA*** genes under salt stress by qRT- PCR**. The relative quantification method (2^−ΔΔCT^) was used to evaluate quantitative variation under different treatment time points. Error bars represent standard error for three replicates. Stars represent significant levels of Student's *t*-test results of overall gene expression at each time point after the treatment, compared to that at hour 0. ^*^P ≤ 0.05 for *t*-test; ^**^P ≤ 0.01 for *t*-test.

Poplar is an important fast-growing tree species that is widely distributed around the world. Salt stress is one of the most severe abiotic stress factors which often affects the growth and development of plants, resulting in slow growth, yield decrease or even death. Many studies have shown that *FLA* genes are widely involved in the abiotic responses (Huang et al., 2008; Zang et al., 2015). In order to reveal the potential roles of *PtrFLA* genes under abiotic stress, roots of *Populus* were treated by 150 mM NaCl. The expression pattern of 18 genes was analyzed with two treatments at 12 and 24 h (0 h as control) by qRT-PCR (Figure 10). The results showed that four genes (*PtrFLA2/12/20/30*) were up-regulated after 12 h of NaCl stress, and the expression pattern exhibited obviously an upward trend after 24 h of NaCl stress. In addition, five genes (*PtrFLA1/3/21/22/26*) were slightly up-regulated with the extension of stress time. Moreover, three genes (*PtrFLA7/17/24*) were significantly up-regulated after 12 h of NaCl stress but were slightly suppressed at 24 h. Other genes did not show significant changes. There was a common trend with two paralogous gene pairs (*PtrFLA2/12* and *PtrFLA3/21*) under salt stress.

## Discussion

Increasing evidence has demonstrated that FLA proteins have a significant impact on growth and physiological processes of plants, particularly on secondary cell wall growth and ability to respond to stresses. However, most researchers focused on the function of *FLA* genes in annual herbaceous plants, especially *Arabidopsis*, while neglected this family in perennial woody plant species. In the present study, a comprehensive analysis of the *FLA* gene family in *Populus* was conducted, and identified a total of 35 full-length genes that was 1.7 times than that of *Arabidopsis* (Johnson et al., 2003). One of the rational reasons for the larger number of *FLA* genes encoded in the *Populus* genome could be that *Populus* genome was four times bigger than *Arabidopsis*. However, during the process of the evolution of plant genome, a larger scale duplication event may have occurred in the ancestor of *Populus*, which generated a lot of redundant sequences and incomplete coding sequences. Therefore, we found that there were far fewer genes than we had expected. Although the *PtrFLA* gene family has 12 more members in *Populus* than in *Arabidopsis*, the number of the members in class III of poplar was three less than that in *Arabidopsis* (Figure 1), suggesting that FLA proteins have rich diversity in higher plants. The 35 PtrFLAs were considered to divide into four distinct groups based on the similarity of protein structure and sequence. Group B members showed a remarkably high similarity (95.06–73.43%) but group D had an extremely low similarity (23.01–11.11%). The similarities between groups A and C were 93.56–24.33% and 41.31–89.16%, respectively. Peer groups had similar protein structure domain and the proportion was usually greater than 40%, in which the similarity of PtrFLA18 and PtrFLA19 reached 100%. Currently, the significant functional feature of plant *FLA* genes was obtained based on studies of *Arabidopsis*. Thus, detailed phylogenetic and expression analyses of these genes may provide valuable information for forecasting the functions of FLAs in *Populus*. PtrFLA2/12 and AtFLA1 were clustered into a clade and shared a high similarity with each other (about 60%), implying that PtrFLA2/12 may be related to the dedifferentiation of cells and the induction of callus formation as described in AtFLA1 (Johnson et al., 2011). PtrFLA22 and AtFLA2 were assigned to a small clade (60.7% similarity), suggesting that PtrFLA22 may participate in tissue regeneration system induced by hormone that is similar to AtFLA2 (Johnson et al., 2003). PtrFLA6/26 were grouped into the same clade (about 50% similarity) with two *Arabidopsis* proteins, AtFLA11/12, which are possibly responsible for decreasing stem tensile strength and stiffness and altering the cell wall matrix (MacMillan et al., 2010).

The characteristics of FLAs have been widely described by researchers, but the only controversy is whether there should be a signal peptide sequence at the N-terminal of FLAs. All FLA proteins in some plants contain signal peptide, such as *Arabidopsis*, cabbage, and cotton (Johnson et al., 2003; Huang et al., 2008; Li and Wu, 2012), but special members lack signal peptides in wheat, rice, and eucalypt (Faik et al., 2006; MacMillan et al., 2015). There are two possible reasons for this phenomenon. First, those special members contain the basic features (including fasciclin domain, AGP glycosylated region and C-GPI anchor signal) of FLA protein family. Second, although there is no signal peptide, special members have highly homologous genes in *Arabidopsis* or other species. AGP-like regions are highly glycosylated with core protein backbones, which exhibit highly variable length and contain multiple glycosylation sites (Showalter, 2001). The so-called *O*-glycosylation sites refer to two or more non-contiguous [A/S/T]-Pro residues or at least one [A/S/T]-Pro_(2−4)_ (Tan et al., 2003). [A/S/T]-Pro-X(1-10)-[A/S/T]-Pro can also be considered as an *O*-glycosylation site, in which X can not have more than 11 amino acid residues (Schultz et al., 2002; Faik et al., 2006). The shortest glycosylated region in PtrFLAs (PtrFLA2/12) consisted of only four amino acids, with two potential *O*-glycosylation sites. PtrFLA20 had the longest AGP-like glycosylated region, up to 54 amino acids with 14 potential *O*-glycosylation sites. The blocks of more than three [A/S/T]-Pro residues have been found in almost all sequences except PtrFLA5/10/22/28/29; some PtrFLAs had [A/S/T]-Pro-Pro motifs, and only PtrFLA13/19/35 contained [A/S/T]-Pro-Pro-Pro motifs. Based on the schematic diagram of protein structure, we have found two major distinctions between *Populus* and *Arabidopsis* FLAs. The first involves the number of two fasciclin-like domains: only 5 *Populus* members are predicted in comparison to *Arabidopsis* (10 AtFLAs) (Johnson et al., 2003). For another, all AtFLAs have outstanding characteristics of *O*-glycosylation sites (Johnson et al., 2003). However, PtrFLA28 may potentially lack well-identified *O*-glycosylation sites, and the same phenomenon also occurs in wheat, rice and cabbage (Faik et al., 2006; Li and Wu, 2012).

An N-terminal signal peptide and a complete C-terminal signal for the GPI anchor have been predicted in PtrFLA2/20/26 (Figure 2). GPI anchor is synthesized in endoplasmic reticulum, which is added onto the C-terminus of the mature AGP protein while AGP proteins are directed into the reaction site (endoplasmic reticulum) by signal peptides. The matured AGPs are then transferred *via* vesicles to the Golgi apparatus and finally to the extracellular space where they remain attached to the exterior leaflet of the cell membrane (Ellis et al., 2010). Cotton GhFLA1, a protein contains signal peptide and GPI-anchored signal, has been analyzed which confirmed that GFP fluorescence is localized in the cytoplasm when GFP is fused to the C-terminus of GhFLA1 with a truncated GPI anchor (Huang et al., 2013). Moreover, YFP fused to the C-terminus of *Populus* PtFLA6 (the same as PtrFLA17, which contains signal peptide and GPI-anchored signal) leads to a ubiquitous distribution of PtFLA6-YFP fusion protein, when CFP protein is fused to the N-terminus of PtFLA6, and similar results are also observed (Wang et al., 2015). In this study, GFP fused to the C-terminus of PtrFLA2/20/26 could have destroyed the GPI-anchor signal sequence. PtrFLA2/20/26-GFP fluorescence in transformed protoplasts were observed in membrane and cytoplasm (Figure 9). Interestingly, the FLA20/26-GFP fluorescence in transformed protoplasts were observed not only in cytoplasm, but also in some plastids with lightspots (Figure 9). Owing to the fact that the chloroplasts had been labeled with red fluorescence (Chloroplast A), there might be other plastids wrapped with membrane structure (Stiti et al., 2011). Therefore, the signal peptide and GPI-anchoring signal are important to the proper localization of AGP proteins. One possible reason is that the insert of exogenous fragment sequences is affected and blocks the transfer of FLAs to the endoplasmic reticulum for post-translational modifications, influencing their native localization. Another possibility is that the native localization of PtrFLAs may depend on the specific developmental stage and specific tissue. The protoplasts used for subcellular location are lack of cell wall. In brief, the subcellular location of PtrFLA2/20/26 in our study is similar to PtFLA6, a cell wall protein identified by immunogold labeling analysis but showing a ubiquitous distribution of PtFLA6-YFP fusion protein (Wang et al., 2015).

Microarray data provide a good clue to detecting changes in gene expression. The temporal and spatial expression patterns of *PtrFLA* genes can provide a theoretical basis for the analysis of gene functions. Public microarray data showed the expression profiles of the 32 *PtrFLA* genes in this study: most genes were found to exhibit preferential or specific expression in different tissues (Figure 6). PtrFLA1/6/21/24 were preferentially or specifically expressed in roots and buds. *PtrFLA15/19* were preferentially expressed in roots and phloem. It was worth mentioning that these six genes (*PtrFLA1/6/15/19/21/24)* were clustered into the same clade that belonged to group A. *PtrFLA3/14/16/20/22/24/26/30* were mainly expressed in roots. Most of these genes remained active in spring and summer, according to plant growth and survival rule. Microarray data may imply that *PtrFLA* genes are required for proper development of the apical meristem and the composition of vascular bundles. The similar expression patterns of *FLA* genes were also observed in other plants. For example, *AtFLA11/12* were strongly expressed in all parts of the inflorescence stem (MacMillan et al., 2010). In *Z. elegans, ZeFLA11* was selectively expressed in differentiating xylem cells of the stem bundles (Dahiya et al., 2006). Previous studies of *popFLAs* revealed that *Pop1-10* were specific to tension wood xylem tissues, while *Pop11-15* were present both in opposite and tension wood zones (Lafarguette et al., 2004). In pine (*P. taeda*), a FLA-like gene, *Ptx14A9*, was preferentially expressed in xylem, especially during xylogenesis (Yang et al., 2005). Similarly, several *EgrFLAs* were specifically and highly expressed in stems (MacMillan et al., 2010, 2015). *PtFLA6* was preferentially expressed in the xylem tissues of middle and basal stems (Wang et al., 2015). Ptx14A9, popFLAs and ZeFLA11 were orthologous to AtFLA11/12 and the specific expression patterns of these genes in different parts of stem might infer to play an important role in cell wall differentiation. According to qRT-PCR results, some *PtrFLAs* (*PtrFLA1/9/10/11/17/23/24/26/28*) also displayed high transcript levels in differentiating xylem and stem (Figure 8), suggesting that these *PtrFLAs* might have a significant impact on primary/secondary cell wall development. In other meristems, some *PtrFLAs* (*PtrFLA12/21/22/24/27/28/30*) were highly expressed in roots, two *PtrFLAs* (*PtrFLA2/12*) were remarkably expressed in apex shoots, while several *PtrFLAs* (*PtrFLA2/3/7/20/21/22/30*) were highly expressed in leaf buds, indicating that they may participate in the regulation of the development of root apical meristem (RAM) or shoot apical meristem (SAM). Moreover, the expression levels of two *PtrFLAs* (*PtrFLA11/30*) showed slight differences among the selected vegetative samples except the young leaves, suggesting that they may play an important role in cell differentiation or main structural components of cells. In reproductive organs, qRT-PCR results showed that some *PtrFLA* genes (*PtrFLA1/2/3/7/11/12/20/21/22/24/26/30*) were highly expressed in male and female flowers, indicating that these genes may play more important roles in the development of inflorescences, which is similar to *AtFLA3* that presents comparatively higher transcript abundances in floral buds and maintains the structure of pollen integrity (Li et al., 2010).

In term of functional prediction, it is difficult to make clear conclusions because of the lack of experimental data on most of clustered FLA proteins. Several studies have proved that the *FLA* genes play certain roles in the regulation of gene expression to correspond to the adverse external circumstances positively. For example, *fla4* (*sos5*) was identified as a salt-hypersensitive mutant in *Arabidopsis*, which affects root growth and morphology during salt treatment (Shi et al., 2003; Seifert et al., 2014). Meanwhile, the expression levels of five *AtFLC* (*AtFLC2/8/9/13/20*) genes were down-regulated under salt stress (Ma and Zhao, 2010). In crops, two rice *OsFLA* (*OsFLA10/18*) and two wheat *TaFLA3/4* genes under salt stress were significantly down-regulated with qRT-PCR detection methods (Faik et al., 2006; Ma and Zhao, 2010). Our result of qRT-PCR also confirmed that some *PtrFLA* genes respond to salt stress. Interestingly, the expression level of some *PtrFLA* (*PtrFLA2/12/20/21/24/30*) genes was up-regulated under salt stress, which is similar to *OsFLA23* and *TaFLA12* (Faik et al., 2006; Ma and Zhao, 2010). Although the physiological significance of these differences is still unknown, PtrFLAs share many other common features with *Arabidopsis*, wheat or rice FLA proteins. For example, PtrFLA2/12/20 proteins were clustered with AtFLA4 and TaFLA3/12 (Class-III) suggesting they may share similar physiological functions under salt stress. It would be interesting to confirm this prediction with poplar RNA interference plants. Moreover, *TaFLA3* was significantly down regulated by dehydration, heat and ABA treatment meanwhile *AtFLA3* was highly up-regulated by cold treatment (Faik et al., 2006; Ma and Zhao, 2010) Taking these data together, we are tempted to conclude that Class III may function in signaling pathway during abiotic stresses such salt, dehydration, heat, or cold. To sum up, the characterization of *Populus FLAs* and the expression analysis described above will provide the foundation for further detailed studies, which could help design experiments to select specific genes for solving problems related to agriculture or ecology.

## Conclusion

This study showcases the full range of *PtrFLA* gene family, including phylogeny, gene structure, chromosomal location, subcellular localization, gene duplication, tissues-specific expression and response to salt stress. These data are helpful for better comprehending the functional and biochemical properties of *PtrFLA* genes and provide theoretical foundation for subsequent work.

## Author contributions

LZ and TZ conceived and performed all the experiments and drafted the manuscript. XS, YC, CD, and WZ were involved in designing and directing the experiments, and revising the manuscript. XS and QH contributed to the conception of the study, gave advice on experiments, and finalized the manuscript.

### Conflict of interest statement

The authors declare that the research was conducted in the absence of any commercial or financial relationships that could be construed as a potential conflict of interest.

## References

[B1] Bailey T. L. Elkan C. (1994). Fitting a mixture model by expectation maximization to discover motifs in biopolymers. Proc. Int. Conf. Intell. Syst. Mol. Biol. 2, 28–36. 7584402

[B2] Dahiya P. Findlay K. Roberts K. McCann M. C. (2006). A fasciclin-domain containing gene, *ZeFLA11*, is expressed exclusively in xylem elements that have reticulate wall thickenings in the stem vascular system of *Zinnia elegans* cv Envy. Planta 223, 1281–1291. 10.1007/s00425-005-0177-9 16328545

[B3] Deng W. Wang Y. Liu Z. Cheng H. Xue Y. (2014). HemI: a toolkit for illustrating heatmaps. PLoS ONE 9:e111988. 10.1371/journal.pone.0111988 25372567 PMC4221433

[B4] Eddy S. R. (1998). Profile hidden Markov models. Bioinformatics 14, 755–763. 10.1093/bioinformatics/14.9.755 9918945

[B5] Eisenhaber B. Wildpaner M. Schultz C. J. Borner G. H. Dupree P. Eisenhaber F. (2003). Glycosylphosphatidylinositol lipid anchoring of plant proteins. Sensitive prediction from sequence- and genome-wide studies for *Arabidopsis* and rice. Plant Physiol. 133, 1691–1701. 10.1104/pp.103.023580 14681532 PMC300724

[B6] Ellis M. Egelund J. Schultz C. J. Bacic A. (2010). Arabinogalactan-proteins: key regulators at the cell surface? Plant Physiol. 153, 403–419. 10.1104/pp.110.156000 20388666 PMC2879789

[B7] Faik A. Abouzouhair J. Sarhan F. (2006). Putative fasciclin-like arabinogalactan-proteins (FLA) in wheat (*Triticum aestivum*) and rice (*Oryza sativa*): identification and bioinformatic analyses. Mol. Genet. Genomics 276, 478–494. 10.1007/s00438-006-0159-z 16944204

[B8] Harpaz-Saad S. McFarlane H. E. Xu S. Divi U. K. Forward B. Western T. L.. (2011). Cellulose synthesis via the FEI2 RLK/SOS5 pathway and cellulose synthase 5 is required for the structure of seed coat mucilage in *Arabidopsis*. Plant J. 68, 941–953. 10.1111/j.1365-313X.2011.04760.x 21883548

[B9] Hu B. Jin J. Guo A. Y. Zhang H. Luo J. Gao G. (2015). GSDS 2.0: an upgraded gene feature visualization server. Bioinformatics 31, 1296–1297. 10.1093/bioinformatics/btu817 25504850 PMC4393523

[B10] Huang G. Q. Gong S. Y. Xu W. L. Li W. Li P. Zhang C. J.. (2013). A fasciclin-like arabinogalactan protein, *GhFLA1*, is involved in fiber initiation and elongation of cotton. Plant Physiol. 161, 1278–1290. 10.1104/pp.112.203760 23349362 PMC3585596

[B11] Huang G. Q. Xu W. L. Gong S. Y. Li B. Wang X. L. Xu D.. (2008). Characterization of 19 novel cotton *FLA* genes and their expression profiling in fiber development and in response to phytohormones and salt stress. Physiol. Plant. 134, 348–359. 10.1111/j.1399-3054.2008.01139.x 18507812

[B12] Huber O. Sumper M. (1994). Algal-CAMs: isoforms of a cell adhesion molecule in embryos of the alga *Volvox* with homology to *Drosophila* fasciclin I. EMBO J. 13, 4212–4222. 7925267 10.1002/j.1460-2075.1994.tb06741.x PMC395348

[B13] Hurst L. D. (2002). The Ka/Ks ratio: diagnosing the form of sequence evolution. Trends Genet. 18, 486. 10.1016/S0168-9525(02)02722-1 12175810

[B14] Johnson K. L. Jones B. J. Bacic A. Schultz C. J. (2003). The fasciclin-like arabinogalactan proteins of *Arabidopsis*. A multigene family of putative cell adhesion molecules. Plant Physiol. 133, 1911–1925. 10.1104/pp.103.031237 14645732 PMC300743

[B15] Johnson K. L. Kibble N. A. Bacic A. Schultz C. J. (2011). A fasciclin-like arabinogalactan-protein (FLA) mutant of *Arabidopsis thaliana, fla1*, shows defects in shoot regeneration. PLoS ONE 6:e25154. 10.1371/journal.pone.0025154 21966441 PMC3178619

[B16] Kawamoto T. Noshiro M. Shen M. Nakamasu K. Hashimoto K. Kawashima-Ohya Y.. (1998). Structural and phylogenetic analyses of RGD-CAP/beta ig-h3, a fasciclin-like adhesion protein expressed in chick chondrocytes. Biochim. Biophys Acta. 1395, 288–292. 10.1016/S0167-4781(97)00172-3 9512662

[B17] Kong H. Landherr L. L. Frohlich M. W. Leebens-Mack J. Ma H. de Pamphilis C. W. (2007). Patterns of gene duplication in the plant *SKP1* gene family in angiosperms: evidence for multiple mechanisms of rapid gene birth. Plant J. 50, 873–885. 10.1111/j.1365-313X.2007.03097.x 17470057

[B18] Lafarguette F. Leple J. C. Dejardin A. Laurans F. Costa G. Lesage-Descauses M. C. (2004). Poplar genes encoding fasciclin-like arabinogalactan proteins are highly expressed in tension wood. New Phytol. 164, 107–121. 10.1111/j.1469-8137.2004.01175.x 33873473

[B19] Letunic I. Doerks T. Bork P. (2012). SMART 7: recent updates to the protein domain annotation resource. Nucleic Acids Res. 40, 302–305. 10.1093/nar/gkr931 22053084 PMC3245027

[B20] Li J. Wu X. (2012). Genome-wide identification, classification and expression analysis of genes encoding putative fasciclin-like arabinogalactan proteins in Chinese cabbage (*Brassica rapa* L.). Mol. Biol. Rep. 39, 10541–10555. 10.1007/s11033-012-1940-1 23053954

[B21] Li J. Yu M. Geng L. L. Zhao J. (2010). The fasciclin-like arabinogalactan protein gene, *FLA3*, is involved in microspore development of *Arabidopsis*. Plant J. 64, 482–497. 10.1111/j.1365-313X.2010.04344.x 20807209

[B22] Lin Y. C. Li W. Chen H. Li Q. Sun Y. H. Shi R.. (2014). A simple improved-throughput xylem protoplast system for studying wood formation. Nat. Protoc. 9, 2194–2205. 10.1038/nprot.2014.147 25144270

[B23] Livak K. J. Schmittgen T. D. (2001). Analysis of relative gene expression data using real-time quantitative PCR and the 2^−ΔΔCT^ Method. Methods 25, 402–408. 10.1006/meth.2001.1262 11846609

[B24] Loopstra C. A. Puryear J. D. No E. G. (2000). Purification and cloning of an arabinogalactan-protein from xylem of loblolly pine. Planta 210, 686–689. 10.1007/s004250050061 10787065

[B25] Lynch M. Conery J. S. (2000). The evolutionary fate and consequences of duplicate genes. Science 290, 1151–11155. 10.1126/science.290.5494.1151 11073452

[B26] Ma H. Zhao J. (2010). Genome-wide identification, classification, and expression analysis of the arabinogalactan protein gene family in rice (*Oryza sativa* L.). J Exp Bot. 61, 2647–2668. 10.1093/jxb/erq104 20423940 PMC2882264

[B27] MacMillan C. P. Mansfield S. D. Stachurski Z. H. Evans R. Southerton S. G. (2010). Fasciclin-like arabinogalactan proteins: specialization for stem biomechanics and cell wall architecture in *Arabidopsis* and *Eucalyptus*. Plant J. 62, 689–703. 10.1111/j.1365-313X.2010.04181.x 20202165

[B28] MacMillan C. P. Taylor L. Bi Y. Southerton S. G. Evans R. Spokevicius A. (2015). The fasciclin-like arabinogalactan protein family of *Eucalyptus grandis* contains members that impact wood biology and biomechanics. New Phytol. 206, 1314–1127. 10.1111/nph.13320 25676073

[B29] Majewska-Sawka A. Nothnagel E. A. (2000). The multiple roles of arabinogalactan proteins in plant development. Plant Physiol. 122, 3–10. 10.1104/pp.122.1.3 10631243 PMC1539237

[B30] Niwa Y. (2003). A synthetic green fluorescent protein gene for plant biotechnology. Plant Biotechnol. 20, 1–11. 10.5511/plantbiotechnology.20.1

[B31] Petersen T. N. Brunak S. von Heijne G. Nielsen H. (2011). SignalP 4.0: discriminating signal peptides from transmembrane regions. Nat. Methods 8, 785–786. 10.1038/nmeth.1701 21959131

[B32] Schultz C. J. Rumsewicz M. P. Johnson K. L. Jones B. J. Gaspar Y. M. Bacic A. (2002). Using genomic resources to guide research directions. The arabinogalactan protein gene family as a test case. Plant Physiol. 129, 1448–1163. 10.1104/pp.003459 12177459 PMC166734

[B33] Schultz C. J. Ferguson K. L. Lahnstein J. Bacic A. (2004). Post-translational modifications of arabinogalactan-peptides of *Arabidopsis thaliana*. Endoplasmic reticulum and glycosylphosphatidylinositol-anchor signal cleavage sites and hydroxylation of proline. J. Biol. Chem. 279, 45503–45511. 10.1074/jbc.M407594200 15322080

[B34] Seifert G. J. Roberts K. (2007). The biology of arabinogalactan proteins. Annu. Rev. Plant Biol. 58, 137–161. 10.1146/annurev.arplant.58.032806.103801 17201686

[B35] Seifert G. J. Xue H. Acet T. (2014). The *Arabidopsis thaliana* Fasciclin like arabinogalactan protein 4 gene acts synergistically with abscisic acid signalling to control root growth. Ann. Bot. 114, 1125–1133. 10.1093/aob/mcu010 24603604 PMC4195540

[B36] Shi H. Kim Y. Guo Y. Stevenson B. Zhu J. K. (2003). The *Arabidopsis SOS5* locus encodes a putative cell surface adhesion protein and is required for normal cell expansion. Plant Cell 15, 19–32. 10.1105/tpc.007872 12509519 PMC143448

[B37] Showalter A. M. (2001). Arabinogalactan-proteins: structure, expression and function. Cell. Mol. Life Sci. 58, 1399–1417. 10.1007/PL00000784 11693522 PMC11337269

[B38] Showalter A. M. Keppler B. Lichtenberg J. Gu D. Welch L. R. (2010). A bioinformatics approach to the identification, classification, and analysis of hydroxyproline-rich glycoproteins. Plant Physiol. 153, 485–513. 10.1104/pp.110.156554 20395450 PMC2879790

[B39] Stiti N. Missihoun T. D. Kotchoni S. O. Kirch H. H. Bartels D. (2011). Aldehyde dehydrogenases in *Arabidopsis thaliana*: biochemical requirements, metabolic pathways, and functional analysis. Front. Plant Sci. 2:65. 10.3389/fpls.2011.00065 22639603 PMC3355590

[B40] Suyama M. Torrents D. Bork P. (2006). PAL2NAL: robust conversion of protein sequence alignments into the corresponding codon alignments. Nucleic Acids Res. 34, 609–612. 10.1093/nar/gkl315 16845082 PMC1538804

[B41] Tamura K. Dudley J. Nei M. Kumar S. (2007). MEGA4: molecular evolutionary genetics analysis (MEGA) software version 4.0. Mol. Biol. Evol. 24, 1596–1599. 10.1093/molbev/msm092 17488738

[B42] Tan L. Leykam J. F. Kieliszewski M. J. (2003). Glycosylation motifs that direct arabinogalactan addition to arabinogalactan-proteins. Plant Physiol. 132, 1362–1369. 10.1104/pp.103.021766 12857818 PMC167076

[B43] Tan L. Showalter A. M. Egelund J. Hernandez-Sanchez A. Doblin M. S. Bacic A. (2012). Arabinogalactan-proteins and the research challenges for these enigmatic plant cell surface proteoglycans. Front. Plant Sci. 3:140. 10.3389/fpls.2012.00140 22754559 PMC3384089

[B44] Thompson J. D. Gibson T. J. Plewniak F. Jeanmougin F. Higgins D. G. (1997). The CLUSTAL_X windows interface: flexible strategies for multiple sequence alignment aided by quality analysis tools. Nucleic Acids Res. 25, 4876–4882. 10.1093/nar/25.24.4876 9396791 PMC147148

[B45] Toshio Y. Eduardo B. (2005). Developing salt-tolerant crop plants: challenges and opportunities. Trends Plant Sci. 10, 615–620. 10.1016/j.tplants.2005.10.002 16280254

[B46] Tuskan G. A. Difazio S. Jansson S. Bohlmann J. Grigoriev I. Hellsten U.. (2006). The genome of black cottonwood, *Populus trichocarpa* (Torr. & Gray). Science 313, 1596–1604. 10.1126/science.1128691 16973872

[B47] Wang H. Jiang C. Wang C. Yang Y. Yang L. Gao X.. (2015). Antisense expression of the fasciclin-like arabinogalactan protein *FLA6* gene in *Populus* inhibits expression of its homologous genes and alters stem biomechanics and cell wall composition in transgenic trees. J. Exp. Bot. 66, 1291–1302. 10.1093/jxb/eru479 25428999 PMC4339592

[B48] Xu H. G. (2004). The Halophyte and Salinization Ecological Governance. Beijing: China Agricultural Science and Technology Press.

[B49] Yang S. H. Wang H. Sathyan P. Stasolla C. Loopstra C. A. (2005). Real-time RT-PCR analysis of loblolly pine (*Pinus taeda*) arabinogalactan-protein and arabinogalactan-protein-like genes. Physiol. Plantarum 124, 91–106. 10.1111/j.1399-3054.2005.00479.x

[B50] Zang L. Zheng T. Su X. (2015). Advances in research of fasciclin-like arabinogalactan proteins (FLAs) in plants. Plant Omics 8, 190–194. Available online at: http://www.pomics.com/zheng_8_2_2015_190_194.pdf

[B51] Zhao Y. Zhou Y. Jiang H. Li X. Gan D. Peng X.. (2011). Systematic analysis of sequences and expression patterns of drought-responsive members of the *HD-Zip* gene family in maize. PLoS ONE 6:e28488. 10.1371/journal.pone.0028488 22164299 PMC3229603

[B52] Zheng T. Li S. Zang L. Dai L. Yang C. Qu G. Z. (2014). Overexpression of two *PsnAP1* genes from *Populus simonii* × *P. nigra* causes early flowering in transgenic tobacco and *Arabidopsis*. PLoS ONE 9:e111725. 10.1371/journal.pone.0111725 25360739 PMC4216142

