# Supplementary material for: Genome-Wide Analysis of the Fasciclin-Like Arabinogalactan Protein Gene Family Reveals Differential Expression Patterns, Localization, and Salt Stress Response in Populus
Source: Front Plant Sci. 2015 Dec 23;6:1140. doi: 10.3389/fpls.2015.01140 (PMC4688393; doi:10.3389/fpls.2015.01140)
Supplement: Supplementary file 1 [file Table1.DOC]

**Supplemental table 1.** The coding sequences of *PtrFLA* genes in *Populus trichocarpa*

| Name | Coding Sequence |
| --- | --- |
| *PtrFLA1* | ATGGAACGATTACAACACCTCTTGATCTCATTATATCTGCTTATTCTCTCCATCAATTTAACCACAACCACAGCTCAATCACCTGCGCCCGCTCCTGCACCACCCGGCCCCACTAATGTCATCAAAATACTCAAAAAGGCTGGCCACTTCAAGACCTTCATCCGCCTTTTGAAATCCACTCAATTGGATAGCAATCTAAACTCCCAGCTTGGCAACACAAACAATGGCTTGACCATCTTTGCTCCAAGTGATAGTGCATTTTCAGCCCTTAAAACAGGCACTCTCCGCACCTTAACTGATCAAGAAAAGGTCGAGTTGATGCAGTTTCACATTGTCCCTATGTTCATCTCATCTTCCCAATTTGATACCGTGAGTAGCCCTTTAAAAACACACGCTGGATCTGGTGCTAGGTTTCAGCTTAACGTTACCGCCAGTGGGAACTCCTTGAACATATCTACAGGACTTACCAACACCACCATTTCCGACACTGTATACACGGACACCCATCTTGCTATTTATCAGGTTGACAAGGTGCTCCTTCCTTTGGATATATTTACCCCTAAACCTCCACCACCGGCACCTGCACCGGCACCAAAGCTTAAAGCAGAGTCAGAGAGCCCTGATGATGCTGTTTCTAAGAAGGATATTTCTAGCGCTGTAAGTTTTGTCATGCATCATGATACGGTGTTCTTTACAGTTGGTACTGTTGTTGCAATATCGTTTTCTTTGTGA |
| *PtrFLA2* | ATGCAGCTTACTGTACTCCTTTCTCTACTTTTTCTCCTCTCCACCGCCACCACCATCACTTATGGCCACAACATTACTTCCATACTAGGCCAGCACCCATCTCTCTCCACTTTCAATCACTACCTCACCCTAACCCACCTCGCCGGAGAAATCAATCGGCGTACAACCATCACCGTCTGTGCCGTCGACAACGCAGCCATGTCTGAAATCCTTTCAAAAAAGCCATCAATCTCCACCATCAAAAACATCCTTTCCCTCCACGTCCTCTTGGATTACTTCGGTACCAAGAAGCTCCACCAGATAAGAGACGGCACCGCGTTGGCAGCCACCATGTTCCAGGCCACCGGGTCAGCTCCTGGGTCTACCGGGTTCGTTAACATAACGGATGTTAAAGGAGGGAAAGTGGCTTTTGGACCGAAAGATAATGGAGGGAACCTTGATGTGTTTTATGTCAAGTCTGTAGAGGAGATACCCTACAACATTTCAGTTATACAGATCAGTAAGTTGTTGCCATCGGATGTGGCGGCGGCGCCCACACCAGAGCCAAGTGCTATGAATATAACGGATATAATGTCAGCTCATGGGTGTAAGGTTTTTGCTGATACTTTGATTGCAAACCCTGATGCTTCAAAGACATATCAGGACACTATTGATGGAGGATTAACAGTGTTTTGCCCTCTTGACGATCCATTTAAGGCCTTTTTACCAAAATTCAAGAATTTAACAGCTTCTGGCAAAGAGTCACTCCTTCAATTCTTCGGTGTTCCTGTTTATCAGTCATTAGCAATGTTAAAATCCAACAATGGAATCATGAACACCTTAGCTACAAATGGGGATAAGAAGTTTGATTTCACAGTGCAAAATGATGGCGAGGATGTGACCCTCAAGACAAGGGGTACCACTGCAAAAATAGTTGGGACTTTGATCGATGAGCAGCCACTTGCTATATACTCCATTGATAAGGTCTTGTTGCCCAAGGAATTGTTTAAAGCAGCACTGACCCCAGCTCCAGCACCTGCCCCGGAGGAGGCGGCAGATGCACCAAAATCTAGCAAGCATAAGAAGCCCTCCGCCGATGATGCGCCATCAGACTCACCAGCTGATTCACCTGATGGCGACGCAGCAGACCAAACAGCTGATAACAATGCTAGTGTGAGACTTGATGGTGGAAGACTTGTTGCCATGGTATTGAGTTTGTGCTTAGGGCTTTTGATGCTGTAG |
| *PtrFLA3* | ATGGAGTTTTCCATGATTATTATGTTTAGTAGCACGCTGTTGTTTTTGTGCACTCCAGTAGCATATGCTCAAACGGCAGCTAGTCCTCCAGCACCAACCCCAACTCCGTCCTCATCACCAGCACCAGCACCAACACCTCCTTACGTTAGCCTCACTGATTTGCTCTCTGTTGCTGGCCCATTCCACACCTTCCTTAGCTACCTTGAATCCACTAAGGTCGTTGACACCTTCCAAAACCAAGCCAACAACACTGATGAAGGCATTACCATCTTCGTACCAAAAGATGATGCCTTCAAAAATCTTAAGAAGCCTTCTTTGTCAAACCTAACTCAAGACCAGGTGAAGCAACTCATTCTTTTCCATGCCTTGCCGCACTATTACGCCTTGGCTGATTTCAAGAACCTTAGCCAAGTAAGCCCTGTTAGCACATTTGCTGGTGCAGGCGGATATGCTTTGAATTTCACAGATGTATCTGGGACCGTGCACCTTGATTCAGGATGGTCAAAAACCAAAGTTAGCAGTAGTGTGCATTCAACTGATCCCGTTGCAGTTTATCAAGTTGACAAAGTCCTCCTTCCTGAGGCGATATTTGGTGCTGATATACCTCCAGCCCCAGCCCCAGCCCCAGCCCCTGAAACCAGCCTTGCTGCAGATTCTCCATCATCTGACAGTACTGGAGATGGGAGCGCTCCAGGAACTTCCCCTCCAAATTCTTCTTATAGGATCTTTGGTGTAGATATTTGGAGTCAATTGGTTTTGGCACTCATAGGTGTGCTGGTCCTGTTCTTGTAG |
| *PtrFLA4* | ATGAGGCAACAATCTTCAAGCTTATTATTCTCTCTTATCCTTTTCTTTCTCCATTGTACAAAAACGTCAGGTCAGTCACCGGCGGCAGCCCCGGTAATGCCACCACCTACGACCCCAGTGAAGGCACCGCCTACGGCCCCTTCACAGGCACCATCTGCACAGGTAGCAACATCACCTGGCCCCGTTGACGTCATTAAAATCCTGCAAAAGGCTGGCCACTTCACTGTCTTCGTCCGCCTAATGCAAGCTACAACAGAAGACACTGAGTTAAACAAGGAGCTGAACAAGACAAACAATGGAATAACAATCTTTGCACCAAGTGACAGCGCATTTTCGAACCTCAAAGCGGGCTTTCTAAACGCTCTAAGCGATGAAGACAAGACTGAGTTGGTGAAGTTTCATGTACTACCTGCACTTATATCATCTTCCCAATTCCAGACTGTCAGTAATCCTGTAAGGACACAGGCGGGAACGGGTCCCAGGGTAACACTGAATGTTACCACCACAGGGAATTTCGTGAACATAACTACAGGGCTGACAAATACAAGCATATCCGGCACCGTATACACTGATAGCCAGTTTGCTATTTATCAAATTGATAAGGTGCTATTTCCTTTGGACATTTTTACTCCTAAGCCTCCTGCTCCTGCTCCTGCACCCGAACTGGGAAAGCCAAGGAAGGCAGCTCCCGGTGTAGAAAGTCCTACCGCTCCTAAGGATATTTCTGGTGCTCTAACACCACTTATTCTGCATAATAATGCCTTGCTCCTTGCAGTCAGCTGTATGGTTGCTGCAATCTTTTCATGA |
| *PtrFLA5* | ATGGAGTCTTCTCCGAAACTCTCTATTCTCTTAATTCTCTCGCTCTACATAATCATCTCCTCCACTTCCATTGACGGCGTAGAAACCACCACTTTCAGCTCAAATCTCTCTCCGCAATCACCACAACCACAAATCTCCACCTCCGATCACTTCCACGACCATTCGTTCTCCTCACACACAAATCTCCTCGCGCCAATCCTCTCTCACCTAGGATTCACTCAACTCGCCATGGCGGTTCCATCTCTCCCCGCTGATTCCACCACCACGGCCTGGTCAGGTCCTTCCACTCTTTTCGCTCCTTCCGATTCCTCTCTCCGTACCTGCTTCTCTTGCTCTATCCCTGACCTCCTCCACGAACACATCGTACCCGGTCTCTTCTCCATCGATTACCTCCGTAAACTCGCTTTCGGTACCAAAATCGAAACGCTAAGTCCTGGCCGTTGTATAACCGTCACTTCCACTTCACTAAAAAACGACTCCGCCACTCCTTCCACCGTCAAGGTCTTCATAGGAGGCGTAGAGATCACGCATCCTGATCTCTTCAACAACGGTGTCTTAATCATTCATGGCATCCAAGGCTATATTGCACCACTCTCTCCATTCTCTTGCGATTTTGAGAGATTGAGCTCTCTTTCTTTTCCGTTTCAAGAGGGCGTGACTCCTCACGTGACCTCAACTACTCATCAACAGGGTATCGGTACTCTGGTGCAGCCTGCTATAATGCGTCTGATGTTACGTGATGCAATGCTTAGGTTGCGTAGTAATGGATTCACGATTCTCTCTCTTGCAATGAGAGTGAAGTACCCTGAGCTAACGAATCTGGTTAATATGACTGTGTTTGCTCTTGATGATGTGTCGATCTTCTCTGGATCGCATGGGTATATTAGCAGCGTGAGGTTCCATATTGTGCCTAATCATTACTTGAGCACTGCTGATTTAGAGAGGCTTCCTGTGGGAGCTACTTTGCCTACGCTAGAGAGAGGTCAGGCTTTGGTGGTTACTTCTGCTGGTGGATTGACTGGATTCAATACCGCGGTGCCGATGAGGATCAACTATGTGAGAGTTAAGGTGCCTGATGTGATGAGGAACTTGAAGATAGTTGTTCATGCTGTTTACTTGCCTTTCCCAAGGATTCATCCCACTTCTGCTGCTGCTTTTGATGAGATGATGGGCATTGGTGGTGAAGGACAGAATATAGTGGCAGCGGAAGATGGTGCTTGTTCTGCGGTTTTTGAGGAGGACGGTAGCTGTGGGACGGTGCCTCCTATGCCAGCTCAGGTCAAGCCATCTGTGGTGGTGCGGAGTGATGAAGATCACCATGGCCTGTAG |
| *PtrFLA6* | ATGAGAAAGCAACTCCTCTCCCCATTCGTTCCTTTCTTGATGTTCTTCCTCTACGGCTCCACGACTGTTGCTCAAACCCCATCACCAGCACCTTCAGGTC  CAACCAACATCACCGCAATCCTCGAGAAGGCTGGTCAGTTCACAACCTTGATTCGGTTAATGAAAAGCACCCAAGAGGCTGACCAAATCAACACACAACTAAACAACTCAAACCAAGGCCTAACAGTATTTGCACCACCTGACAACGCCTTTACTAACCTCAAAGCAGGCACGCTGAATTCACTCAGCGATCAACAAAAGGTCCAACTGGTGCAATTCCACATCATTCCAAATTTCTTTTCCATGTCAAGCTTCCAAACTGTGAGTAATCCCTTGCGTACTCAGGCCGGTAACAGTGCCGACGGCGAGTTCCCGCTAAATGTGACAACATCAGGGAATCAAGTGAACATAACAACAGGGGTTAATACTGCAACAGTGGCTAACACTATATTCACTGATGGCCAGTTAGTTGTGTATCAGGTGGATCAGGTCCTTCTGCCATTAGATCTCTTTGGTACAGCGGCAGCACCAGCACCTGCACCTTCAAAGCCTGATAAAGATGTTCCAGCCAAAGCTCCTGCAGGGTCAAAGGAAGATGCCTCTGTTGATGCTTCAGGTGCAACCATTGCAACTGTATCTGTCAGTGTCGTGCTGATCGCAGCAATTTCATTGAAGCTATGA |
| *PtrFLA7* | ATGGATTCTCACATCTATGGTGTCTCTAAGAAAACCCTTCTTCTCTTTACTCTTCTCTGTCTTTCCGTTTCCTCCATTTCTGCATTACCCCATCAGAATAAAACTGGCAATAGTACGGGTACTGGTCAAATGATAAACTCCAACTCGGTGCTTGTTGCGCTTCTTGACTCGCATTACACCGAGTTAGCTGAGCTCGTTGAAAAGGCTCTCCTTCTACAAACCCTTGAAGAAGCTGTTGGCAAACACAACATCACCATCTTTGCGCCAAGAAATGAAGCTTTAGAGCGTCAACTTGACCCCGAATTCAAACGGTTTTTACTTGAACCCGGTAATCTCAAATCTCTCCAAACCCTTGTATTGTTCCACATTATCCCCCAACGGGTCGGATCCAATGACTGGCCAGGTCACAAATCAAACCCCAGCAGGCACACCACTCTCTGCAACGATCATCTGCACTTGATCACCAAGAATTCAGGCAAAAAGGTTGTCGGATCCGCCGACGTGACCCGACCCGACGACGTGACCCGACCGGACGGTGTTATTCATGGCATTGAGCGGCTCCTAGTCCCACAGTCAGTCCAGGAAGACTTCAACAGGAGAAGAAATTTGAGATCCATATCAGCTGTATTGCCTGAAGGAGCCCCGGAAGTTGACCCAAGAACCCACAGATTGAAAAAACCGGAACCACCAGTTCGGGCCGGTTCACCGCCGGTTTTGCCCATTTATGATGCCATGGCTCCTGGCCCATCATTGGCTCCCGCCCCAGCTCCTGGACCCGGTGGACCTCACCACCACTTCGACGGAGAAAGCCAAGTTAAAGACTTTATACAGACACTACTACTCTACGGTGGCTACAATGAAATGGCTGATATTTTAGTGAACTTAACTTCATTAGCCACTGAAATGGGCAGGTTGGTATCTGAAGGCTATGTGCTTACAGTTTTGGCACCAAATGATGAAGCCATGGCTAAGCTAACAACAGACCAGCTGAGTGAACCAGGGGCACCAGAGCAGATTATTTACTACCATATAATACCTGAGTACCAAACTGAAGAGAGTATGTATAATGCTGTTAGGAGGTTTGGGAAAATAGGGTATGATACATTGAGGTTGCCACATAAAGTCGCGGCGCAAGAAGCTGATGGGTCGGTTAAGTTCGGGTCAGGTGATGGGTCGGCCTATTTGTTTGACCCGGATATCTATACAGATGGGAGGATTTCAGTTCAAGGGATTGATGGGGTTTTGTTTCCTGAAGTTGAGAAAGAGAGTACTTCTGTTAAGAAATCCGTCAGCTCTGTTAAGGTTGCCACTACCAAGCCAAGAAGAGGGAAATTAATGGAAGTAGCCTGTATAATGCTTGGAACTCTTGGACAGGACTCGCGTTTCACTACATGTCAGTGA |
| *PtrFLA8* | ATGGCCATGGCGCCTTCACCTCCTTGCATCCACATTTTCTTCGCTTCTATTCTTCTTCTCTCAAATTTTCATCTGGGGTTCTCTTCTTCCTCCTCTACATTGCAAGAGAACCATAGCAATGGGTCTTATTCAGGCCAAATCAACTCAAACTCAGTTCTTGTAGCTCTTCTTGACTCACATTATACAGAACTGGCTGAACTTGTTGAAAAGGCACTTCTTTTACAAACCCTTGAAGATGCTGTTGGTAAACACAACATCACCATCTTTGCTCCTAGAAATGAAGCTCTCGAACGTGATCTTGATCCCGAGTTCAAGCGGTTCTTGCTTGAACCTGGCAATCTCAAGTCTCTCCAGACTCTCCTACTTTATCACATTGTCCCCAACAGAATCAATCTCAGCCATAACTCTTCTCTTCACCATCACAGCACCTTGTGCCGCGATAGAATCAAGCTCGGCAGTCAATCCGGCGAGAAGTTAATAGACTCGGCGAAAATCATTCAAGTGAATGCAGTGGAGAGGCCAGATGGCGTAATTCATGGGATTGAAAGGTTGCTAATCCCACGATCTGTTCAACAAGATTTCAACAATCGCAGGAGTTTGCAATCAATTTCAGCTGTGAAACCAGAAGGAGCACCAGAGGTTGATCCAAGAACACAAAGGTTGAAGAAACCAGCACCACCAGCAAAACCCGGTTCAGCCCCGGTCCTACCAATCTACGATGCAATGGCACCCGGCCCATCTCTTGCCCCGGCTCCAGCTCCTGGTCCAGGTGGACCTCATCACCATTTCAATGGAGAAAGACAAGTTAAAGATTTCATTGAAACCCTTCTTTTGTATGGAGGATACAATGAAATGGCTGATATTCTTGTGAATTTAACATCATTAGCAACAGAAATGGGAAGATTAGTATCTGAAGGTTATGTGCTAACAGTTTTAGCCCCAAATGATGAAGCAATGGCTAAGCTCACAACAGACCAATTGAGTGAGCCAGGAGCACCGGAGCAGATAATCTATTACCATGTGATACCCGAGTATCAAACTGAAGAAAGCATGTACAATGCTGTTAGAAGATTTGGGAAGATTTCTTATGATACATTAAGATTGCCACACAAAGTTTTGGCACAAGAAGCTGATGGGTCAGTGAAATTTGGACATGCTGAGAATTCTGCTTATTTGTTTGATCCTGATATTTACACAGATGGAAGAATCTCTGTTCAAGGGATTGATGGGGTTCTGTTTCCATTAGAGGAGAAGGAGAAATCGGATACCAAGACGGAAATGAAGAGTGTTAAGGTTGCTGCTAAGCCGCAAAGGAGAGGGAAGTTGCTTGAAGTGGCTTGCAGGATGCTCGGGACATTTGGGCAAGATTCACATTTCACCACATGCCAGTAA |
| *PtrFLA9* | ATGTATTTTTTTTATTCTGTACAATACAGGCCACGCCTCTGCCCAAGAATGCAACCATTTATCTTGTTATTCTGGCTCCTCTTCCTCCATGCTTGCTCCCAAACATTTTGCCAATCACCAGCCCAGTCACCAGCAGCGACTCAGACAAAGGCACCAGTGCCACCGCCACCCCCAGCTGGCCCTACAGACACCATCCAAATCCTCTTAAAAGCTGGCCGCTTCTTATCCTTTGTCCGCCTGATGAAAGCTACCCATGTGGATACCCAGTTATTTTCCCAGCTTAACAGCTCAACCGATGGCATAACCATGTTTGCACCAAATGATAACGCATTTTCAAGCCTAGTTGCTGGGGCCGTAGGCTCCCTAAACGATAGAGAAAAACTCGAGTTTGTGCAATTTCACATACTACCGAGATTTCTTTCAATTTCCGATTTCCAGACTCTAAGTAACCCTGTAAAAACCCTGGCAGGATCAGACCGGAAGTTCCCGCTTACTATAACCACCAGTGATAACTCAGTGACAGTAAGTTCAGGGCTTACGAAAACAAGCATATCAAACACTATATACACAGACAAGCAGGTTGCTATTTATGAGGTCGACAAGGTGCTAGTTCCTAAAGATCTTTTCCCACCGGCGCCTCCAGCTCCAGCACCGGCAAGGCCTCTTGCAGAGCCTGATCCTGTTGCTCCCAGGGATGCTTCTAGTGCACTAGTTATTGCTTGGCAACATCGTGTTAACGTTGTGCTCTTCGGGGCTGGCCTCTACATAGCTGCTCTAGTAATGGATCCATAA |
| *PtrFLA10* | ATGAGGCCACAATCTTTCATCTTAGCATTGTCTCTTATCTTTTTCTTCCTCCATTGTACAAAAACGTTGTGCCAGTCACCTGCCGCGGCCCCGGCTATGGCGCCCCCTAAGACACCAGTGAAGGCACCGCCTGCAGACTCCTCACAGGCACCATCTGCACAGGTTGCTACATCACCTGGCCCCGTTGACGTCAATAAAATCTTGCAGAAGGCTGGCCACTTCACAGTCTTCGCCCGCCTGATGCAAGCTACAACAGAAGACACCGAGTTAAACAAGGAGCTGAACACCACAAACAATGGAATAACAATCCTGGCACCAACTGACAACGCATTTTCGAGCCTCAAAGCAGGCTTTCTCAACTCTCTAAGCGATGAAGACAAGACTGAGCTGGTGAAGTTTCACGTGCTGCCTGCATTTATATCAACATCCCAATTTCAGACTGTGAGTAACCCTGTAAGGACACAGGCGGGGACAGGTCCCAGGGTAACACTAAATGTCACCACCACCGGGAATTTCGTGAATATAAGTTCAGGGCTGACAAATACAAGCATATCAGGCACTGTATACACTGACAGCCAGCTTGCTATTTATCAACTTGATAAGGTACTATTTCCTTTGGACATATTTACTCCTAAGCCTCCAGCCCCTGCTCCCGAGCCAGCACTGGGAAAGCCTAGGAAAGCAGCACCCGATGCAGAGAGCCCTACTGCTCCTAAGGATATTTCTGGTGCTCCAGCTCTGCTTTTTCTGCATAACAACGCCTTGCTTCTTGCAGTCAGCTGTGCGTTTGGTGCAATAATACATTCATGA |
| *PtrFLA11* | ATGGCCATCGCACTATCTTCTTTCAACATTTTCTTCACATTTCTTCTTGTCTCTACTTTCCATCTGGGGTTTTCTTTTTCTGCATTGCAAGAAAACCATAGTAATGGCACTTATTCTGGCCAAATCAACTCAAACTCCGTTCTTGTAGCCCTTCTCGACTCCCATTATACAGAACTAGCTGAACTCGTTGAAAAAGCACTTCTTTTACAAACCCTTGAAGATGCTGTTGGTAAACACAATATCACCATCTTTGCTCCTAAGAACGAAGCTCTTGAACGCGATCTTGATCCTGAATTCAAGAGGTTCTTGCTTGAACCTGGCAATCTCAAGTCTCTCCAGACTCTTTTACTTTACCACATTGTACCCAACAGAATCAATCCTAGCCATAACTCTTCTCTTCAACATCACAGCACTTTGTGCCGCGATAGAGTCAAACTCAGCAGTCAGGAATCCGGCGAGAAGTTAATAGATTCGGCGAAAATCATTCAGGTGAATGCAGTGGAGAGGCCAGATGGAGTGATTCATGGGATAGAAAGGTTGCTAATCCCACGATCTGTTCAGCAGGATTTCAACAATCGCAGGAGTTTGCAATCAATCTCAGCTGTGAAACCAGAAGGAGCACCAGAGGTTGATCCAAGAACACACAGGTTGAAGAAACCAGCACCACCAGCAAAACCCGGTTCAGCCCCGGTGCTACCAATCTATGATGCAATGGCGCCCGGCCCATCTCTTGCCCCAGCTCCAGCTCCTGGTCCGGGTGGACCTCATCACCATTTCAATGGAGAAAAACAAGTTAAAGATTTCATTGAAACCCTTCTTTTATATGGAGGATACAATGAAATGGCTGATATTCTTGTGAATCTAACATCATTAGCAACTGAAATGGGAAGATTGGTCTCTGAAGGGTATGTGCTAACAGTTTTAGCCCCAAATGATGAAGCAATGGCTAAGCTTACAACGGACCAGTTGAGTGAGCCGGGAGCACCAGAGCAGATAATCTATTACCATGTGATACCTGAGTATCAAACTGAAGAAAGCATGTACAATGCTGTTAGAAGATTTGGGAAAATTTCTTATGATACATTAAGATTGCCGCACAAGGTTTTGGCAGAAGAAGCTGACGGGTCTGTGAAATTTGGACACACTGAAAATTCTGCTTATTTGTTTGATCCTGATATTTACACAGATGGGAGAATTTCGGTTCAAGGGATTGATGGGGTTTTGTTTCCATTAGAGGAGAAGGAGAAATCGGAGACCAAAAAGGAAATTAAGAGCGTTAAGGTTGCTGTTAAGCCACAAAGGAGAGGGAGGTTGCTTGAAGTGGCTTGCCGGATGCTTGGGACATTTGGACAAGATTCACATTTCACCACATGCCAGTAA |
| *PtrFLA12* | ATGCAGCGGCTTACTATACTCCTTTCTCTACTTTTCCTCCTCTCCACTTCCACCACCTTCACTCGTGGCCACAATATTACTCATATACTTGGCAAGCACCCATCTTTCTCCACTTTCAACCACTACCTCACCCTAACCCACCTCGCCGGAGAAATCAACAGCCGAAACACCATCACCGTCTGCGCCGTCGACAACGCAGCGATGTCTGAACTCCTTTCAAAACACCCATCAATCGCCACCATCAAAAACATCCTTTCCCTCCATGTCCTCTTGGATTACTTCGGCACCAAGAAACTCCACCAGATACGAGAGGGCACGGCCTTGGCAGCCACCATGTTCCAGGCCACCGGGTCGGCTCCTGGGTCGACCGGATTCGTTAACATAACGGATGTTAAGGGAGGGAAAGTGGCTTTTGGACCTGAGGATAATGAAGGGAATCTTGATGTCTTTTATGTCAAGTCTTTGGAGGAGATACCTTACAACATCTCCGTTATACAGATCAGTAAGGTGTTGCCGTCGGACGTGGCAGCGGCGCCTACACCAGAGCCAAGTGCTATGAATATAACAGATATAATGTCAGCTCATGGGTGTAAGGTTTTTGCTGACACTTTGATTGCAAATCCTGAAGCTTCAAAGACATATCAGGACAGTGTTGATGGAGGATTAACAGTGTTTTGCCCTCTCGACGATCCATTCAAGGCCTTTTTCCCTAAATTCAAGAATTTAACAGCTTCTGGCAAAGTGTCATTTCTTGAATTCTTCGGTGTCCCTATTTATCAGTCCTTAGCTATGTTAAAATCCAACAATGGAATCATGAACACTTTAGCTACAGATGGCGAAAAGAAGTTTGATTTCACAGTTCAAAATGATGGTGAGGATGTGACACTCAAGACCAGGAGTATCACTGCAAAAATAGTTGGGACTTTGATCGACGAACAGCCACTAGCTATATATACCATTGATAAGGTCTTGCTGCCGAAGGAATTGTTTAAAGCAGCACCAACACCAGCTCCAGCGCCCGCCCCGGAGAAGGAGGTGGCTGATGCACCAAAATCTAGCAAACATAAGAAGCCATCCTCCGATGTTGTTCCATCAGACTCACCGGCCGATTCACCTGATGGCGACCTAGCAGATCAAACAGCTGATGACAATGCTAGTGTGACATTATATGGTGGAAGACTTGTTGCCATGTTATTGAGTTTGTGCTCAGGGCTTTTGCTGCTGTGA |
| *PtrFLA13* | ATGAAGCACCATTTTTCAGTCTTCTTATTTCCAGCTATCCTTCTCTTGCTCCACTGCACCCAAACACTATCCCAGACACCTACAGCAGCACCAGCAAAAGCACCTGCTGCAGCCTCTGCACCTCCGCCAGCTGCGACCTCGTCTGCACAGGCCTCCCCGCCTGTTATGGTTCCTGTACAAGTTTCAAAAGGTCCTGTCAATGTCATTAAAATCCTTCAGAAGGCTGGCGGCTTTGCGGTCTTTATCCGTCTTATAAAATCCACTCAAGAGGACATCCAAGTATTCTCCCAGCTCAATGACTCGAGAGATGGTGTCACCATCTTTGCCCCTACTGATGGGGCCTTTTCAGCCATTATCAAATCAGGCGTTCTTAACTCCCTAAGTGATCATCAGAAGATTGAGTTGGTGCAGTTTCATATTATACCAAAAATTCTTACAACTGCTAATTTTCAGACTGTCAGTAACCCTATAACGACACTGGCAGGATCTGGTAGTCGTTTTGCTCTTAATGTGATAACCACAGAAAATATGGTGAACGTAACTTCAGGACTTACCAATACAAGCGTATCGGCAATTGTATACACTGATAGCCAGCTTGCTGTCTATCAGGTCGACAAGGTGCTACTTCCTTTGGATATTTTTGCCCCTAAACCTCTTGCTCCTGCTCCAGCACCACCAAAGCCTAAAAAGGATGATGGCGCAGAGAGTCCTCTGGTTCCTGAGGATACTTCCAGTGCTGTAAGCTGCATCCCGCTTAACTCCCTGATCATCTTTGGAGCTGGTATGGTTGCTGCAGTTTTTACTTTGTGA |
| *PtrFLA14* | ATGGTGCCACAATTTCTGTTATCATTTTCACTTATTCTATCCTTTCTTCTTCATTGCCCCCCAACCCTAGCTCAGTCACCAGCTGCAGCCCCAGGACCACCTGGTCCAACCAATGTCACCAAAATACTAGAAAAAGGTGGTCAGTTCAGTGTGTTTATCCGGCTTTTGAAAGCCACTCAAGAGGATGTCACATTGAATGGCCAGCTCAACAACACAAACAATGCTATAACCATCTTTGCACCTAGTGACAACGCCTTTTCGAGCCTGAAATCAGGCACCCTCAACTCCCTAAACGATCAAGAAAAGGCTGAGTTAGTGCAGTTTCACATTATCCCACAATATCTATCAAGTTCCCAATTCCAGACAGTGAGCAACCCTTTGACCACACAGGCAGGATCAGGTGGCAGGTTAGAGCTTAATGTAACCACCACAGGTAACTCAGTGAATATAACCACAGGACTTACCAATACAAGCGTATCTGGCACTATATACACTGATAACCAGCTAGCTGTTTATCAGGTTGACAAGGTGCTCCTTCCTGTTGATATTTTTACACCTAAACCTCCTACTCCAGCACCAGCACCTGAAAAACCAAAGAAAAGGTCTAAAGCTGCAGAGAGTCCAGATGCTCCTGAGGATAATTCTGGAGCAGTAAGCTTAACTGTTCTTAATGATGTTGTGTTCTTTGGAGTGGGCATTGTTGCAGCAATATTTTCTTTGTGA |
| *PtrFLA15* | ATGAAGCAACAGTACTACTCACTCTTCTCCTTTTCATTTTTCCTTCTTTTCCTCCATTGTACCACTACCTTTGCTCAGACGTCACCAGCTGCAACCCCAGCACAGGCACCAGCTGTAGTTGTAGCACAACCTCCAGCTGCAACCCCGACACAGGCAGCTCAACCACACGGCATCACAAACGTCACCAAAATCCTTGAGAAGGCTGGCCATTTCACGATCTTTATTCGCCTTTTGAGATCCACCCAAGAGGAAAACCACTTATTCTCCGCACTTAATGATTCAAGCTCTGGTGTAACCATCTTTGCACCAACTGATAGCGCATTTTCGGAACTCAAATCAGGAACTCTCAACACTCTAAGTGATGGTGACAAGTCTGAGTTGGTGAAGTTCCATGTAGTTCCTACTTTCTTATCGACCTCCCAGTTCCAGACCGTAAGTAACCCTCTCGGAACATGGGCTGGAACAGGTAATAGGTTACCACTTAATGTCACAAGTTATCCAAACTCAGTGAACATAACCACAGGACTTACCAATACCAGTTTATCCGGCACGGTATACACGGACAACCAGCTAGCCATTTACAAGATTGAGAAGGTGCTACTTCCTAAGGACATTTTTGCTTCAAAGGCTCCAGCTCCAGCACCGGTGGCACCTGCACCAGAAAAGCCTACAAAGGCAGTTCCTGCAGCAACCGTAGAGAGTCCCGTGGCCCCTGTGGATACATCTAGTGCACTTATGTTCACACAAAATCATGTGGTGGGATCAGTTGCCATATTTGCTGCTGCAATGTTTGCTCTGTAA |
| *PtrFLA16* | ATGGCTACCACTCCCCTGTCTTTCTTTCTCCTTTCACTCCTCTCCCTCTCCCTCAATGCTCAAGCTCAAACCCCGACAGCACCCGCCCCGACGCCCTCCGGCCCGGTTAACTTCACCGCAGTTCTTGTAAAGGGTGGACAGTTCGCCACACTCATTCGCCTTCTGAACAACACTCAAACTTTGAACCAAATTGAGAATCAGCTTAACAGCTCTTCTGAGGGCATGACTATCTTCGCCCCAACCGATAATGCCTTCAACAATCTGAAAGCTGGTGCTTTAAATGGCCTCAACCAACAAGAACAAGTTCAGCTTCTTCAATACCACACGTTGCCTAAATTCTATACCATGAGCAATCTTTTGCTGGTTAGTAACCCTGTTCCCACTCAGGCCTCAGGCCAAGATGGTGTCTGGGGTCTTAACTTCACTGGCCAAAGCAACCAAGTCAATGTATCAACAGGGCTTGTTGAGGTTCAAATTAACAACGCTTTAAGACAAGATTCTCCTTTGGCAGTTTACCCAGTCGACAAAGTCTTGTTGCCCGAGGCATTGTTTGGTGTTAAGCCACCGACTGCATCACCTCCGGCACCATCAAGCAAGTCCAATTCAACAGTGGCGGCTGCAGAACCATCAACAGGTAAGAACTCAGCTGGTGGAAGGAATGTAGCATTGGGGTTGGTTGTTGGGCTCGGTTTGGTTTGCATGGGAATTCTTTCTTGA |
| *PtrFLA17* | ATGAAGCAACAGTACTCAATCTTCTCATTTTCAATGCTTCTTCTTTCCCTGTGTTATATCAATACCTTTGCCCAGTCACCAACAGCAGCCCCAGCACAGGCACCAGCAGTAGTCGTAGCACAACCTCCAGTCGCAACCCCAACACAGGCTGCTGCACCACACGGCATCACCAACGTCACCAAAATCCTTGAGAAGGCTGGCCATTTCACGATCTTTATCCGCCTTTTGAGATCCACCCAAGAGGAAAACCACTTGTTCTCCGCGCTCAATGATTCCAACACTGGTATAACCATCTTTGCACCCACAGATAGTGCATTTTCGGAACTCAAATCAGGAACTCTCAATACTCTAAGTGATGGAGACAAGTCTGAGTTGGTGAAGTTCCACGTAGTTCCAACATTTCTATCGACTTCCCAGTTCCAGACCGTAAGTAATCCTCTTGGAACATGGGCTGGAACAGGTAGTAGGTTACCACTTAATGTCACAAGTTATCCAAACTCGGTGAACATAACCACAGGACTTACCAATACAAGTTTATCTGGCACGGTTTACACGGACAACCAGCTAGCCATTTATAAGATCGAGAAGGTGCTACTTCCTAAGGACATTTTTGGATCCAATGCTCCAGCTCCAGCACCAGTACAGGCACCTGCAGAAAAACCTACTAAGGCGGTTCCTTCTGCTAATGTAGAGAGTCCTGTTGCTCCTGTGGATATATCTAGCGCAGTTACGTTTATGCATAATAATGTGGTTGGATCACTCGTTATAGTTGCTGCTGCAATGTTTGCTTGTAATGTAGAAGGGTTTTGA |
| *PtrFLA18* | ATGAAGCAACAGTACTCAATCTCCTCCATTTCAGTTTTCCTTCTTTTCCTCCACTATACCAATACCTTTGCCCAGTCACCAGCAGCAGCCCCAGCGCAGGCACCAGCAGTAGTCGTAGCACAACCCCCAGCCGCAACTCCAACACAGGCAGCTGCTCCACACGGCATCACCAACGTCACCAAAATCCTTGAGAAGGCTGGCCACTTCACGATCTTTATCCGCCTTTTGAGATCCACCCAAGAGGAAAACCACTTGTTCTCCGCGCTTAATGATTCAAGCTCTGGTGTAACCATCTTTGCACCCACAGATAGTGCATTTTCGGAACTCAAATCAGGAACTCTCAATACTCTAAGTGATGGTGACAAGTCTGAGTTGGTGAAGTTCCACGTAGTTCCAACATTCCTATCCACTTCCCAGTTTCAGACTGTAAGTAATCCTCTTGGAACATGGGCTGGAACAGGTAGTAGGTTACCACTTAACGTAACAAGTTATCCAAACTCGGTGAACATAACCACAGGACTTACCAATACAAGTTTATCTGGCACGGTATACACGGACAACCAGCTAGCCATTTATAAGATTGAGAAGGTGCTACTTCCTAAGGACATTTTTGCTTCTAAGGCCCCAGCTCCAGCACCAGCACCTGCACGAGAAAAACCTACTAAGGCGGTTCCTGCTGCTAATGTAGAGAGTCCTGTTGCTCCTGTGGATATATCTAGCGCAGTTACGTTTATGCATAATAATGTGGTTGTTGGATCACTCGTTATAGTTGCTGCTGCAATGTTTGCTTGTCATGTAGAAGGGTTTTGA |
| *PtrFLA19* | ATGAAGCAACAGCACTCACTCTCCTCTTTTTCATTTTTTCTTCTTTTACTCCATTGTGCTAATACCTTTGCTCAGTCACCAGCTGCAACCCCAGCACAGGCACCAGCAGCAGTTGTAGCACAACCTCCAGCTGCAACCCCAACACAGGCAGCTCAGCCACACGGCATCACAAACGTCACCAAAATCCTTGAGAAGGCTGGACATTTCACGATCTTTATCCGCCTTTTGAGATCCACCCAAGAGGAAAACCACTTATTCTCCGCGCTTAATGATTCAAGCTCTGGTGTAACCATCTTTGCACCAACTGATAGCGCATTCTCGGAACTCAAATCAGGAACTCTCAACACTCTAAGTGATGGAGACAAGTCTGAGTTAGTGAAGTTTCATGTAGTTCCTACTTTCTTATCGACCTCCCAGTTCCAGACCGTAAGTAACCCTCTCGGAACATGGGCTGGAACAGGTAGTAGGTTACCACTTAATGTCACAAGTTATCCAAACTCAGTGAACATAACCACAGGACTTACCAATACAAGTTTATCTGGCACAGTATACACGGACAACCAGCTAGCCATTTATAAGATTGAGAAGGTGCTACTTCCTAAGGACATTTTTGCTTCAAAGGCTCCAGCTCCAGCACCGGTAGCACCTGCACCAGCAAAGCCTACAAAGGCAGTTCCTGCAGCAACCGTAGAGAGTCCCGTGGCTCCTGTGGATATATCTAGTGCACTTATGTTCGCACATAATAATGTGGTGGGATCAGTTGGCATAGTTGCTGCTGCAATGTTTGCTCTGTAA |
| *PtrFLA20* | ATGGCTACGTTACAGTACTCTCTCCTTCTTTCCTTCACACTATCAGCACTCGTCTCCACCATCTTAGCCCACAACATAACCGACATTCTCTCAGGCTTCCCGGAATACTCCGAGTTCAACAAGTACCTTACCCAAACAAAACTAGCCGATGAAATCAACACTCGCCAGACAATAACTGTCTTGGCTCTCAACAATGGGGCTATGACTGCTCTTGCAGCCAAACATCCACTTTCAGTCATCAAGAATGCCCTTAGTCTTCTTGTTGTCCTTGATTACTATGACCCAACTAAACTCCATCAGATCTCCAAAGGCACCACGTTATCCACCACTCTCTACCAAACTACTGGAAATGCCCCTGGTAACCTCGGATTCGTCAACATTACAGATCTCCAAGGTGGTAAAGTCGGCTTCGGCTCCGCTGCTCCCGGCTCGAAACTTGATTCCTCTTACACTAAATCTGTGAAGCAAGTCCCTTATAATATTTCAATTCTTGAAATCAGCCAGCCGATTATAGCTCCAGGGATTTTAACAGCTCCGGCCCCAACACCGTCAAGTGTGAACATTACGGCGTTACTCGAAAAGGCTGGATGCAAAACATTTGCGAGTTTGTTACAAACTAGCGGTGTCATTAAAACGTATCAGTCAGCGGCGGATAAAGGTTTAACTATTTTTGCTCCAAACGACGAGGCGTTCAAGGCTGCTGGAGTACCTGATCTGAGCAAGCTCACCAATGCTGAAATAGTCTCGCTCTTACAGTACCATGCCACGGCTACTTACAGTCCGTTTGGGAGTTTGAAAACTAGCAAAGACCCGATCTCTACGTTGGCTAGTAACGGTGCAGGTAAGTTCGATCTGACGGTTACAAGTGCGGGTGACTCGGTGACTCTACACACTGGAATCGGTCCGTCGCGAGTTGCTGAAACGGTCCTTGACTCAACACCGCTAGTGATTTTCACTGTTGATAATGTGCTGCTACCGGTTGAGTTGTTCGGGAAAGCTCCGTCTCCAGCCCCGGCAGGGGAACCGGTGAGTGCACCGAGTCCGAGTCCGGTTGCATCGAGTCCAGCTCCGGCTAGTGTTGAGGCTCCATCTCCTCTCGCTGCTTCACCCCCTGCTCCGCCTGTGGAGACGCCTGGTGGTGCCCCAGCTGAGACGCCGTTTGGATCCGAGAATAGCACGGCGGATGGTAGTGCTGCTGTTCACGTGAGTGTACCTGTTCAGGTCACTGTCTTTGCCACTGTAATTTGTTCGATTCTCATGTCCTGA |
| *PtrFLA21* | ATGAAGTTTTCTATGATTATTGTGCTTAGCAGCACACTGCTGTTTTCGTGCACTCCACTAGCATATGCTCAAAAAGTAGCTAGTCCTCCAGCACCAACCC  CAACTCCATCCCCAGCACCAGCACCATCACCTCCTTACGTTAACCTCACTGATTTACTCTCTGTTGCTGGCCCATTCCACAACTTCCTTAACTACCTTGA  GTCCACTAAAGTCATTGACACCTTTCAAAACCAAGCCAACAACACTGATGAAGGCATTACCATCTTCGTACCAAAAGATGATGCCTTCAAAAATCTCAAGAAGGCTTCTTTGTCAAACCTAACTCAAGACCAGCTCAAGCAACTCATTCTTTTTCATGCCTTGCCACATTATTACTCGTTGTCTGATTTCAAGAACCTTAGCCAAGTGAGCCCTGTCAGCACATTTGCTGGTGCAGGAGGATATGCTTTGAATTTCACCGATACATCTGGGACCGTGCACCTTGATTCAGGATGGTCTAAAACTAAAGTTAGTAGTAGTGTGCATTCAACTGATCCTGTTGCAATCTATCAAGTTGACAAAGTCCTCCTTCCTGAGGCAATCTTTGGTACTAATATACCTCCAACCCCAGCTCCAGCACCAGCTCCTGACACTAGCCCTACTGCAGATTCCCCAACATCTGACGACTCAGCAGGAGCAGGGAGCGCCCCAGGAAAGTCCCCCCCAAATTCTTCTTATAGGATCAATGGTGTAGGTATTTGGAGTCAATTGGTTCTAGCTATTGCAGGTGTGCTGGTCCTGTTTTTGTAA |
| *PtrFLA22* | ATGCCACGCCCACTACCTCTCCTCACTCTCGCCATATCTCTAGTCCTCCTTGCCTCCACCACAACTGTAAATGCACACAACATCACACGCATACTAGCCAAACACCCTCAATTCTCCACCTTCAACCACTACCTCACAGTCACCCACCTCGCCGCAGAAATCAATCGCCGCCAGACCATTACTGTCCTGGCTCTCGACAATGCTGCCATGTCATCTTTAATATCCAAGCAGCTCTCTGTTTACACTCTAAGGAATGTTCTCTCTTTACACGTTCTTGTTGATTACTTTGGTACCAGGAAACTCCACCAGATTACTAATGGTACTGAATTGACTGCCACCATGTTCCAAGCCACTGGTTCAGCCCCTGGCGCCTCAGGTTATGTCAATATTACTGATCTTAACGGTGGTAAAGTGGCATTTGGCGCTGAAGATAACGATGGAAAGCTTAATGCTGTTTATGTCAAGTCTCTCGAAGAGATTCCATATAACATATCCATTTTACAAATCAGTCAGCCTCTGAATTCAGCAGAAGCAGAAGCACCGACGGCGGCGCCAACACTGAATGTAACAGCCATTTTGTCGAATCAAGGTTGTAAAGCGTTCTCCGACTTGTTGATAGCTTCTGGGGCACACACCACATTCGAGGAAAACGTTGACGGAGGATTAACTGTATTTTGCCCCACCGACCCCGTTATCAATGGCTTCATGCCCAAGTACAAGAACTTGACAGCTCCTCAAAAAGTGTCGTTACTGTTATATCACGGTATCCCAATTTACCAGTCACTCCAAATGCTAAAAACCAGCAACGGGATTATGAACACGTTGGCCACAAACGGTGCCAACAAGTACGATTTCACAGTCCAAAATGATGGGGAGGTGGTGACGTTGGAGACGAAAGTTACGACCGCGACGATAACGGGGACGGTAAAAGATGAGGAGCCGTTGGTGGTGTATAAGATTAATAAAGTCTTGTTGCCCAGGGAGTTGTTTAAGGCGGCTCCGGAGAAAAAAGCACCGGCGCCTAAGGGGGAGAAGGATGTGGCGGATGGACCTAATGCTGATGCGCCGTCAGATGAATCGGATGATCAAACGGCAGATAATGATAACGGGGTCAATAAAATGGGCGGTGGGAGATTGGCTGTTGTAGCCCCGAGTTTCTTTTTTGGGGTGGTGATGTTTTTTTTATTTGATTAA |
| *PtrFLA23* | ATGAAGCAACTAATTTCCTTCTCATTTTCTCTTGTCCTTCTCTTCCTCCACTGCACCCAAACACTATCCCAGCCACCAAATGCAGCACCAGCAAAAGCACCTGCTGCAGCCACTGTGCCGCCACCAGCTGCAACGTCTGCTCAAGCCTCGCCACCTGTTATGGTTCCTGTACAAGTTTCAAAAGGCCCTGTCAATGTCATTAAAATCCTCCAGAAGGCTGGCCACTTTGCGTTCTTCACCCGCCTTATAAAATCCACTCAAGAGGACATCCAATTATTCTCTCAGCTCAATGACTCGAGAGATGGGGTCACCGTCTTTGCCCCAACTGATGGTGCCTTTTCGGCCATTATCAAATCTGGTGTTCTTAACTCCTTGACTGATCATCAGAAGATCGAGTTGGTACAATTTCATATAATACCAAGAATTCTGACAACTGCCAATTTTCAAACTGTCAGCAACCCTATAACAACACTGGCAGGATCTGGTAATCGTTTCGCACTTAATGTGATAACTACAGAAAATATGGTAAATGTAACTACGGGACTTACCAATACAAGCGTATCGGCAATTGTATACACAGATAGCCAGCTTGCTATTTATCAGGTTGACAAGGTGCTACTTCCTTTAGATATTTTTGCCCCTAAACCTCTTGCTCCTGCACCAGCACCACCAAAGCCTAAAAAGGATGATGGCGCAGAGAGTCCTATGGTTCCTGAAGATACTTCTGGTTCAGTAATATGCATGGTGCATAATACTCTGCTCATGTTTGGAGTTGGCTTAGTTGCTGCTGCTATTCCTTTGTGA |
| *PtrFLA24* | ATGGTGCCACAATTTCTGTTCTCAGCTTCTTTTATTCTATTCTTCCTTCTTCATTGCCCTCCAACCCTAGCTCAGTCACCAGCTGCAGCCCCAGCACCACCCGGTCCAACCAATGTCACCAAAGTCCTAGAAAAAGGTGGTCAGTTCAGCGTTTTTATCAGGCTTTTGAAAGCCACTCAAGAGGATGTCACATTGAATGGCCAGCTCAACAACACAAACAATGCTATAACCATCTTTGCACCTAGCGATAACGCATTTTCAAGTCTCAAATCAGGCACCCTCAACTCCCTAAGCGATCAAGAAAAGGCTGAGCTAGTACAGTTTCACATTATACCACAATTTCTATCAAGTTCCCAGTTCCAGACTGTGAGCAACCCTCTGACCACACAAGCAGGATCAGGTGGCAGGTTAGAGCTTAATGTAACCACCACAGGAAACTCTGTGAATATAACCACAGGGCTTACAAATACAAGCGTATCCGGCACTATCTACACTGATAACCAGTTAGCTGTTTATCAGGTTGACAAGGTGCTTCTTCCTCTTGATATCTTTACACCTAAACCTCCTACTCCAGCACCAGCACCTGAAAAGCCAAAGAAGAGATCCAAAGCTGCAGCGAGTCCAGAATCTCCTGCGGATACTTCTGGGGCAGTAAGCTTTACTGTTCTGAACAATGTTGTGTTCTTTGGAGTTTGCATGGTTGCAGCAATATATTCTTTGTGA |
| *PtrFLA25* | ATGGATTCTCACATCTATGGTGTCTCCGAGAAAACCCTTTTTCTCTTTACCCTTCTTTGTTTTTCCGTCGCCTCCATTTCTGCATTGCCCCATCAGAATAGAACTGGCAATAGTACGGTTACGGGTCAGATGATAAACTCCAACTCGGTTCTTGTTGCCCTCCTGGACTCGCATTACACTGAGTTAGCTGAGCTCGTTGAGAAGGCTCTCCTTCTGCAAACCCTTGAAGAAGCTGTTGGCAAACACAACATCACCATCTTTGCACCAAAAAATGAAGCTTTAGAGCGTCAACTTGACCCCGAATTCAAACGGTTTTTACTTGAACCCGGTAATCTCAAATCTCTCCAAACCCTTTTGTTGTTCCACATTATCCCCCAACGGGTCGGATCCAATGACTGGCCGGGTCATAAATCAAACCCCACCAGGCACACCACTCTCTGCAACGACCATCTGCACTTAATCACCAAGAATTCAGGCAAAAAGCTTGTCGGAGCCGCCGTGTTGACCCGACCGGATGATGTGACCCGTCCCGATGGAGTAATCCACGGTATTGAACGCCTCCTAGTTCCACAATCAGTACAGGAAGACTTCAACAGGAGAAGAAATCTGAGATCCATATCAGCTGTTTTGCCAGAAGGAGCACCGGAAGTTGACCCCAGAACCCATAGATTGAAAAAACCCGAACCACCAGTTCGGGCCGGGTCACCACCGGTTTTGCCCGTTTATGATGCCATGTCTCCTGGACCATCACTGGCTCCCGCCCCAGCTCCAGGACCCGGCGGACCTCACCACCATTTCGATGGAGAAAGCCAAGTCAAAGACTTCATACAGACGTTAGTACACTATGGTGGCTACAATGAGATGGCTGATATTTTAGTGAACTTAACCTCATTAGCCACTGAAATGGGCAGGTTAGTATCTGAAGGTTACGTGCTTACAGTTTTGGCACCGAATGACGAAGCCATGGCTAAGCTAACAACAGACCAGTTGAGCGAGCCAGGGGCACCAGAGCAGATCATCTATTACCACATAATTCCCGAGTACCAAACTGAAGAGAGCATGTATAATGCTGTTAGGAGGTTTGGGAAAATAGGGTATGATACACTGAGGTTGCCGCATAAAGTTGTGGCTCAAGAAGCTGACGGGTCGGTTAAGTTCGGGTCGGGTGATGGGTCGGCCTATTTGTTTGACCCGGATATCTATACAGATGGGAGGATTTCAGTTCAAGGGATTGATGGGGTTTTGTTTCCTGAAGTTGAGAAAGAAAGTACTTCTGTTAAGAAATCCGTTAGCTCTGTTAAGGTTGCCACTACCACGCCAAGAAGAGGGAAGTTAATGGAAGTAGCTTGTAGAATGCTCGGAAGTCTTGGTCAGGAGTCGCATTTCACCACATGCCAATGA |
| *PtrFLA26* | ATGAGAAAGCAACTTCTCTCCCCATTCGTTCCTTTCTTGATGTTCTTCCTCTACAGCTCCACCACTTTTGCTCAAACCCCATCTCCAGCACCTTCAGGTCCAACCAACATAACGGCGATCCTTGCGAAAGCTGGTCAGTTCACAACCTTAATTCGGTTGTTGAAAAGCACCCAAGAGGCTGACCAAATCAACACACAACTCAACAATTCAAACCAAGGCCTAACAGTCTTTGCACCAACTGATAATTCCTTTGCTAACCTCAAAGCAGGTACACTGAATTCGCTCAGCGACCAACAAAAGGTCCAATTGGTGCAATTTCACATCCTTCCAAATTTCCTTTCCATGTCAAACTTCCAAACTGTCAGCAATCCCTTGCGCACTCAAGCCGGAAATAGTGCTGATGGCGAGTTCCCGCTCAATGTGACAACATCGGGGAATCAAGTGAACATAACAACAGGGGTTAATACTGCAACCGTGGCAAACACTATATACACCGATGGCCAGTTAGTTGTGTATCAGGTGGATCAGGTCCTTCTGCCGTTGGATCTTTTTGGTACAGCACCAGCACCAGCTCCTGCACCTTCAAAGCCCGAGAAAGATGTTCCAGCAAAAGCTCCTGCAGGGTCCAAGGAAGATGCATCTGTTGATAGTTCAGGTGCATCCATTGCAACAGTATCCTTCGGTGTTGTGCTGATCGCAGCAATTTCATTGAAGCTATGA |
| *PtrFLA27* | ATGTCAACCATGCTTCTCTTCCTTCTCATTCTCCTTTTAATCTCTTCCTCAGTTCTTGCAGCTTCTAACCCTTTCTCGAATGCCATGGAGATCCTCTCAACCTCTGGCTATCTCTCCATGGCATTAACCCTTGAAATCACTTCGAAAAGACTCCATCTTGAATCATCTGCTGCAACTATATTCGCTCCATTGGACATTGCATTTGCAAGATTAGGCCAACTTTCTGTTCTTGATCTCCAATATCACATCTCGCCAGTGAGACTTTCTGGGTATTACCTCGATAGTCTTCCTTTTGGTACAAGAATCCCCACGTTGTTGCCAAATCACTCATTGATTGTCACTACAAGTTTGAGTTATTTTGATGGAAAGTTGTCAATCAATGGAATCTCGATTGAAGAATCTGCCTTGGTTGATTTTGGGTCTCTGATCATATTTGGTATGAGTGAGTTCTTCAATTCTTCTCTTGAGATTTCTCCTAATTTGACTCCAGCACCAGCACCAAGTCCTAGTCCAGTTACTAGTCTTGGCAATACTTCTCAGAATGAATCAACAGGCTTGGATGTCGATTTTTTTGGCCAAGCTTCCCATTTACTAATGCCTAGAGGTTATTCAATAATGGGAACATTTCTTGATGCGCAACTGTTTGGGATCAAGAACCAGACAAGATTGACAATCTTTGCACCAGTTGATCAAGCAATGGATGCATATGCAAAGAATGTTAGTGATTATTCATCGATCTTTCGGAAACATGTGGTTCCTGGATTGTTTCCAAGGCAAGATTTGGAAGGGTTCAATGATGGAACAAGTTTGCCAACTTTCTCAGGAGGGTTCATGATTAATTTGACTAAGTCTGGTGATGTGCTTGTGCTTAATGGTGTTCCTGTTATCTTTCCAGACATGTATCAAAGTGACTGGCTGATAATTCATGGCCTGAACCAGCTGCTTACGCCACCATTAAAGGAGGAGGAGTTAGTGGGGGAATCATTCTCAGAACTCGATGGGGCAGAAGATAAGCCAGATGTGCTTGATTTTGATGATTATGTATATGGAGCACCATGA |
| *PtrFLA28* | ATGGGAACCCAGAACTTGATGATCAACAAATCAACGGCCAAGATTCTCCTCCATCTCCTCCTCCTCTCTCTCCTCCACCAAATCACCACCGCTACACTCACCGACCAAGAACTTGACTTTGCCCTCTTATCACTCCGATCATACGGCTACACGCTTTTCCCCAACGCCATCTCCACATCCGACCTCCGCCTCCAACTCCTCAACCAATCAAGTAATGCCACGTCAACTTCAACCTTCACTCTCTTCTGTCCGCCTGACTCCCTCCTCTTCTCTGTTGACCTCGCCTCCACTGCTCCTCACTATACGAAATCCCTCTTCCTCCACGTGTCTCCCTCTCGCCTCTCCACGTCAGACTTGAGAAACCTAACCGCCGCCTCTGGTGGTACCTACATTGATTCATTGGTGCCGAACCACCGTCTCTTGATTACTAACTCTCTGGCTCAGCTAAACGGTACCGTTGATGGGTCCATATTGGTCAATCGGGTTCGGGTTTCAGTTCCGGATCTTTTTCTTGGGTCTGACATTGCTGTTCATGGACTGGACGGGATTCTTGTTGCTGGATTCGATGAAAAAGTCGAAGACACGTCGTTTGAGGCTGCGACGTGGTCTCCTGCGAACGCGATTGGGTCTGCGGAGCAGAATTCTCCACTGGCTGGCAGGTTTCCAGCGAGGAGGAGGAAAGGAAGGAACCATAGGCATAACGGCAGGAACGGTGGCATTAGAAGGAATAATCACCGAGGGCGGAGAATTAACGGTGGCCATCGTCGTGGTGTTGGTAGAAATGTCAGCGGCGGTACTCGTGGTGGTGGTGTTACGCGTGGTGCATTTGCAATGTATAACCATCGACTTTAG |
| *PtrFLA29* | ATGGAAGCTTTCACGACCTTGTTAGTGCTTCTCATGATCAAAGTACTCGTCTGTGCAACTAGCCCAACGGACATTCCTTCAAGAAGCCAAGACCTTGTCGTTGCCTCTGACGAGATGGCAAGAGCCAACTACTTCAGTTTTGTCATGCTCATTAACATGGCCCCACTTGATCAGAAATTTCAGGGGAATGTCACTTTCTTGATGCCCAAGGATCGTTTATTGTCTAAAATCAGGATGCATCAAAACGCTGTTTCTGATTTTTTGCTTCACCATTCAATCCCATCACCCTTGCTCTTTGATCACCTGCGACATATTCCACCAGGCTCGCTGATTCCCAGTTCTGATCCCGATTATATGCTTAACATTTCCAACGAGGGAAGGAAGAGTTTCTTTCTCAACAATGTCAAGATCTCTAGCCCAGATCTATGCACTGCAGGGTCTTCAATTAGATGCCATGGCATCGATGGGGTGCTGCTAGTAGATACGGATAGGCATCCTTTACCTGCTTGCTCCAACAGCACAAGTCCTGCTATTGTGGCCACACCACCAAGCCCATCACTCCCATTACCAGATATTCCATCATTTCCATCATCAGCTCCTCCTCCTGGGGCTGCTGCGCCGACAGACCAAGAGCACATCCCAAAACATTCAGGCTCTTCCCAACTAGAAAGTCTCTCTCTTGGAGGTTTATTAAAATTCATGGCGACTTCTATTTTGGTGTTAAATGCTCGGGTTCTCTATACAGTGGGACAGAATTAA |
| *PtrFLA30* | ATGGCTACCTCTCCCCTCTCTCTCGTTCTCCTTTCACTCTTCCTCTCCCTCTCGCTCCATGCTCAAGCTCAAGCCCCAGCAGCACCTGCTCCGGCGCCCTCCGGCCCGGTCAACTTCACCGCAGTTCTTGTAAAGGGTGGTCAGTTCGTCACATTCATTAGCCTTTTGAACAAGACTCAAACATTTAACCAAATTGAAAATCAGATCAACAGCTCTTCTGAGGGCATGACTATTTTTGCCCCAACTGACAATGCCTTCAGCAATCTTAAATCTGGTGCTTTAAATGGCCTCAGCCAACAACAACAAGTTCAACTTCTTCAGTACCACATGTTGCCTAAATTCTATTCTTTGAGCAATCTCTTGTTGGTTAGTAACCCTGTTCCCACCCAGGCCTCTGGCCAAGAAGGTGTTTGGGGTCTTAACTTTACAGGTCAAAGCAACCAAGTCAATGTGTCAACAGGGCTTGTGGAGGTTCAAGTCAACAACGCTTTAAGGCAAGATTTTCCTCTGGCAGTTTATCCAGTCGACAAAGTGTTGTTGCCTGACGAGTTGTTTGGTGTTAAGCCACCATCTGCTTCACCTCCAGCACCGGCAACTAAGGGGTCATCATCAGGCAAGTCCAATTCATCAGATACGGCTGCAGAGCCATCACCTGGTAAGAACTCAGCTGGTGGAAGGAATGTAGCATTGGGGTTGATTTTTGGGCTTGGTTTCGTTTCCATGGGAATTCTTTCTTGA |
| *PtrFLA31* | ATGAAGCCACAGTACTTACTCTCTTCATTTTCAATTTTTCTTCTTTTCCTCCATTGTCCCAATACCTTTGCCCAGTCACCAGCTGCAGCCCCGGCTCAGGCACCAGCAGTGGTTGCATCACCTCCAGCTGCAACCCCAACACAGGCAGCTGCACCACATGGCATCACCAACGTCACCAAAATCCTTGAGAAGGCTGGACACTTCACGATCTTTATCCGCCTTTTGAGATCCACCCAAGACGAAAACCGCTTATTCTCCGCGCTAAATGACTCAAGCACTGGTTTAACCATCTTTGCACCAACGGATAGCGCATTTTCGGAACTTAAATCAGGAACTCTCAACACGCTAAGTGATGGAGACAAGTCCGAGTTAGTGAAGTTTCACGTAGTTCCAAATTCCTATCTACTTCCCAGTTCCAGACCTTTATCTGGCACCGTATACACAGACAACCAGCTAGCCATTTATAAAATTGAAAAGGTGCTCCTCCCTAAGGACATTTTTGCTTCTAATGCTCCAGCTCCAGCACCAGTGGCATCTGCACCAGAAAAGCCAACAAAGGCAGTTCCTGCAGTTACTGTAGAGAGTCCTGCAGCTTCTGTGGATATATCTAGTGCACTTATCTTTACTCATAATCTTGTGGTGGGATCAGTTGGGTTACTTGCTTCTGCAATGTTTTCTCTGTAA |
| *PtrFLA32* | ATGAAGCAACAGTCAATCTCATTTTTTATTTTCCTCCTTTTCCTCCAATGCACCTATACCTTTGCCCAGTCACCAGCTGCAGCCCCAGCGCAGGCACCAG  CAGTGGTTGTGGCACAACCTCCAGCAGCAACCCCAACACAGGCAGCTGCACCACATGGCATCACCAACGTAACCAAAATCCTTGAGAAGGCTGGCCACTTCACGATCTTTATCCGCCTTTTGAGATCCACCCAAGAGGAAAACCACTTGTTCTCCGCGTTAAATGATTCAAGCACTGGTTTAACCATCTTTGCACCAACGGATAGCGCATTTTCGGAACTCAAATCAGGAACTCTCAACACTCTAAGTGATGGAGACAAGTCCGAGTTAGTGAAGTTCCACGTAATTCCAACATTCCTCTCTACTTCCCAGTTTCAGACTGTAAGTAACCCTCTAGGCACATGGGCTGGAACAGGCAGTAGGTTACCACTTAATGTGACAAGTTATCCAAACTCAGTGAACATAACCACAGGACTTACCAATACAAGTTTATCTGGCACCGTATACACAGACAACCAGCTTGCCATTTATAAGATTGAAAAGGTTCTCCTCCCTAAGGACATTTTTGCTTCTAATGCTCCAGCTCCAGCACCAGTGGCACCTGCACCAGAAAAACCTGCAAAGGCGGTTCCTGCGGCTAATGTTGAGAGTCCTGTGGCTCCTGTGGATATATCCAGTGCAGTTTGGTTTATGCATAATAATGTGGCGGGGTCAGTTGGTATAGTTGCTGCTGCAGTGTTTGCTTTGTAA |
| *PtrFLA33* | ATGAAGCCACAGTACTTACTCTCTTCATTTTCAATTTTACTTCTTTTCCTCCATTGTACCAATACCTTTGCCCAGTCACCAGCTGCAGCCCCGGCTCAGGCGCCAGCAGTGGTTGCATCACCTCCAGCTGCAACCCCAACACAGGCAGCTGCACCGCATGGCATCACCAACGTCACCATAATCCTTGAGAAGGCTGGACACTTCACGATCTTTATCCGCCTTTTGAGATCCACCCAAGAGGAAAACCACTTATTCTCCGCGCTAAATGACTCAAGCACTGGTTTAACCATCTTTGCACCAACGGATAGCGCATTTTCGGAACTCAAATCAGGAACTCTCAACACGCTAAGTGATGGAGACAAGTCCGAGTTAGTGAAGTTTCACGTAGTTCCAACATTCCTATCTACTTCCCAGTTCCAGACCGTAAGCAATCCTCTCGGAACATGGGCCGGAACAGGTAGTAGGTTACCACTTAACGTGACAAGTTATCCAAACTCGGTGAACATAACCACAGGACTTACCAATACAAGTTTATCTGGCACCGTATACACAGACAACCAGCTAGCCATTTATAAGATTGAAAAGGTACTCCTCCCTAAGGACATTTTTGCTTCTAATGCTCCAGCTCCAGCACCAGTGGCAGCTGCACCAGAGAAGCCAACGAAGGCAGTTCCTGCAGTAACTGTAGAGAGTCCTGCAGCTTCTGTGGATATATCTAGTGCACTTATCTTTACTCATAATCTTTTGGTGGGATCAGTTGGGTTACTTGCTTCTGCAATGTTTTCTCTGTAA |
| *PtrFLA34* | ATGAAGCCACAGTACTTACTCTCTTCATTTTCAATTTTTCTTCTTTTCCTCCATTGTCCCAATACCTTTGCCCAGTCGCAAGCTGCAGCCCCGGCTCAGGCACCAGCAGTGGTTGCATCACCTCCAGCTGCAACCCTAACACAGGCAGCTGCACCGCATGGCATCACCAACGTGACCAAAATCCTCGAGAAGGCTGGCCACTTCACGATCTTTATCCGCCTTTTGAGATCCACTCAAGAGGAAAACCACTTATTCTCCGCGCTAAATGACTCAAGCCCTGGTTTAACCATCTTTGCACCAACGGATAGCGCATTTTCGGAACTTAAATCAGGAACTCTCAACACTCTAAGTGATGGAGACAAGTCCCAGTTAGTGAAGTTTCACGTAGTTCCAACATTCCTATCTACTTCCCAGTTCCAGACCGTAGTAGGTTACCACTTAACGTCACAAAGTTATACAAACTCGGTGAACATAACCACAGGACTTACCAATACAAGTTTATCTGGCACCGTATACACAGACAACCAACTAGCCATTTATAAAATTGAAAAGGTGCTCCTCCCTAAGGACATTTTTGCTTCTAATGCTCCAGCTCCAGCACCAGTGGCACCTGCACCAGAAAAGCCAACAAAGGCAGTTCCTGCAGTTACTGTAGAGAGTCCTGCAGCTTCTGTGGATATATCTAGTGCACTTATCTTTACTCATAATCTTGTGGTGGGATCAGTTGGGTTACTTGCTTCTGCAATGTTTTCTCTGTAA |
| *PtrFLA35* | ATGAAGCAGCAGTTAATCTCCTCATTTTCAATATTCCTTTTATTCCTCCATTGTGCCAGTACCTTTGCCCAGATACCAGCTGCAGCCCCAGCGCAGGCACCGGCAGTAGTCGTAGCACCACCTCCAGCTGCAACCCCAACTCAGGCAGCTGCACCGCATGGCATCACCAACGTCACGAAAATCCTCGAGAAGGCTGGCCACTTCACGATCTTTATCCGTCTTTTGAGATCCACTCAAGAGGAAAGCCACTTGTTCTCCGCACTAAATGATTCAAGCACTGGTTTAACCATCTTTGCACCAACGGATAGCGCATTTTCGGAACTCAAATCAGGAACTCTCAACACTCTAAGGGATGGAGACAAGTCTGAGTTAGTGAAGTTTCACGTAGTTCCAACATTCCTATCTACTTCCCAGTTCCAGACCGTAAGCAATCCTCTCGGAACATGGGCCGGAACAGGTAGTAGGTTACCGCTTAATGTCACAAGTTATCCAAACTCGGTGAACATAACCACAGGACTTACCAATACAAGTTTATCTGGAACCGTATACACAGACAACCAGCTAGCCATTTATAAGATTGAAAAGGTACTCCTCCCTAAGGACATTTTTACTTCTAATGCTCCAGCTCCAGCACCAGTGGCACCTGCACCAGAAAAGCCATCAAAGGCAGTTCCTGCAGTAACTGTAGAGAGTCCTGCAGCTTCTGTGGATATATCTAGTGCACTTATCTTTACTAATAATATTCTGGTGGGATCATTTGGTTTACTTGCTTCTGCAATGTTTTCTCTGTAA |
