# Supplementary material for: Genome-Wide Analysis of the Fasciclin-Like Arabinogalactan Protein Gene Family Reveals Differential Expression Patterns, Localization, and Salt Stress Response in Populus
Source: Front Plant Sci. 2015 Dec 23;6:1140. doi: 10.3389/fpls.2015.01140 (PMC4688393; doi:10.3389/fpls.2015.01140)
Supplement: Supplementary file 2 [file Table2.DOC]

**Supplemental table 2.** Precursor protein backbones of PtrFLAs in *Populus trichocarpa*

| Name | Precursor protein backbones |
| --- | --- |
| PtrFLA1 | MERLQHLLISLYLLILSINLTTTTAQSPAPAPAPPGPTNVIKILKKAGHFKTFIRLLKSTQLDSNLNSQLGNTNNGLTIFAPSDSAFSALKTGTLRTLTDQEKVELMQFHIVPMFISSSQFDTVSSPLKTHAGSGARFQLNVTASGNSLNISTGLTNTTISDTVYTDTHLAIYQVDKVLLPLDIFTPKPPPPAPAPAPKLKAESESPDDAVSKKDISSAVSFVMHHDTVFFTVGTVVAISFSL |
| PtrFLA2 | MQLTVLLSLLFLLSTATTITYGHNITSILGQHPSLSTFNHYLTLTHLAGEINRRTTITVCAVDNAAMSEILSKKPSISTIKNILSLHVLLDYFGTKKLHQIRDGTALAATMFQATGSAPGSTGFVNITDVKGGKVAFGPKDNGGNLDVFYVKSVEEIPYNISVIQISKLLPSDVAAAPTPEPSAMNITDIMSAHGCKVFADTLIANPDASKTYQDTIDGGLTVFCPLDDPFKAFLPKFKNLTASGKESLLQFFGVPVYQSLAMLKSNNGIMNTLATNGDKKFDFTVQNDGEDVTLKTRGTTAKIVGTLIDEQPLAIYSIDKVLLPKELFKAALTPAPAPAPEEAADAPKSSKHKKPSADDAPSDSPADSPDGDAADQTADNNASVRLDGGRLVAMVLSLCLGLLML |
| PtrFLA3 | MEFSMIIMFSSTLLFLCTPVAYAQTAASPPAPTPTPSSSPAPAPTPPYVSLTDLLSVAGPFHTFLSYLESTKVVDTFQNQANNTDEGITIFVPKDDAFKNLKKPSLSNLTQDQVKQLILFHALPHYYALADFKNLSQVSPVSTFAGAGGYALNFTDVSGTVHLDSGWSKTKVSSSVHSTDPVAVYQVDKVLLPEAIFGADIPPAPAPAPAPETSLAADSPSSDSTGDGSAPGTSPPNSSYRIFGVDIWSQLVLALIGVLVLFL |
| PtrFLA4 | MRQQSSSLLFSLILFFLHCTKTSGQSPAAAPVMPPPTTPVKAPPTAPSQAPSAQVATSPGPVDVIKILQKAGHFTVFVRLMQATTEDTELNKELNKTNNGITIFAPSDSAFSNLKAGFLNALSDEDKTELVKFHVLPALISSSQFQTVSNPVRTQAGTGPRVTLNVTTTGNFVNITTGLTNTSISGTVYTDSQFAIYQIDKVLFPLDIFTPKPPAPAPAPELGKPRKAAPGVESPTAPKDISGALTPLILHNNALLLAVSCMVAAIFS |
| PtrFLA5 | MESSPKLSILLILSLYIIISSTSIDGVETTTFSSNLSPQSPQPQISTSDHFHDHSFSSHTNLLAPILSHLGFTQLAMAVPSLPADSTTTAWSGPSTLFAPSDSSLRTCFSCSIPDLLHEHIVPGLFSIDYLRKLAFGTKIETLSPGRCITVTSTSLKNDSATPSTVKVFIGGVEITHPDLFNNGVLIIHGIQGYIAPLSPFSCDFERLSSLSFPFQEGVTPHVTSTTHQQGIGTLVQPAIMRLMLRDAMLRLRSNGFTILSLAMRVKYPELTNLVNMTVFALDDVSIFSGSHGYISSVRFHIVPNHYLSTADLERLPVGATLPTLERGQALVVTSAGGLTGFNTAVPMRINYVRVKVPDVMRNLKIVVHAVYLPFPRIHPTS AAAFDEMMGIGGEGQNIVAAEDGACSAVFEEDGSCGTVPPMPAQVKPSVVVRSDEDHHGL |
| PtrFLA6 | MRKQLLSPFVPFLMFFLYGSTTVAQTPSPAPSGPTNITAILEKAGQFTTLIRLMKSTQEADQINTQLNNSNQGLTVFAPPDNAFTNLKAGTLNSLSDQQKVQLVQFHIIPNFFSMSSFQTVSNPLRTQAGNSADGEFPLNVTTSGNQVNITTGVNTATVANTIFTDGQLVVYQVDQVLLPLDLFGTAAAPAPAPSKPDKDVPAKAPAGSKEDASVDASGATIATVSVSVVLIAAISLKL |
| PtrFLA7 | MDSHIYGVSKKTLLLFTLLCLSVSSISALPHQNKTGNSTGTGQMINSNSVLVALLDSHYTELAELVEKALLLQTLEEAVGKHNITIFAPRNEALERQLDPEFKRFLLEPGNLKSLQTLVLFHIIPQRVGSNDWPGHKSNPSRHTTLCNDHLHLITKNSGKKVVGSADVTRPDDVTRPDGVIHGIERLLVPQSVQEDFNRRRNLRSISAVLPEGAPEVDPRTHRLKKPEPPVRAGSPPVLPIYDAMAPGPSLAPAPAPGPGGPHHHFDGESQVKDFIQTLLLYGGYNEMADILVNLTSLATEMGRLVSEGYVLTVLAPNDEAMAKLTTDQLSEPGAPEQIIYYHIIPEYQTEESMYNAVRRFGKIGYDTLRLPHKVAAQEADGSVKFGSGDGSAYLFDPDIYTDGRISVQGIDGVLFPEVEKESTSVKKSVSSVKVATTKPRRGKLMEVACIMLGTLGQDSRFTTCQ |
| PtrFLA8 | MAMAPSPPCIHIFFASILLLSNFHLGFSSSSSTLQENHSNGSYSGQINSNSVLVALLDSHYTELAELVEKALLLQTLEDAVGKHNITIFAPRNEALERDLDPEFKRFLLEPGNLKSLQTLLLYHIVPNRINLSHNSSLHHHSTLCRDRIKLGSQSGEKLIDSAKIIQVNAVERPDGVIHGIERLLIPRSVQQDFNNRRSLQSISAVKPEGAPEVDPRTQRLKKPAPPAKPGSAPVLPIYDAMAPGPSLAPAPAPGPGGPHHHFNGERQVKDFIETLLLYGGYNEMADILVNLTSLATEMGRLVSEGYVLTVLAPNDEAMAKLTTDQLSEPGAPEQIIYYHVIPEYQTEESMYNAVRRFGKISYDTLRLPHKVLAQEADGSVKFGHAENSAYLFDPDIYTDGRISVQGIDGVLFPLEEKEKSDTKTEMKSVKVAAKPQRRGKLLEVACRMLGTFGQDSHFTTCQ |
| PtrFLA9 | MYFFYSVQYRPRLCPRMQPFILLFWLLFLHACSQTFCQSPAQSPAATQTKAPVPPPPPAGPTDTIQILLKAGRFLSFVRLMKATHVDTQLFSQLNSSTDGITMFAPNDNAFSSLVAGAVGSLNDREKLEFVQFHILPRFLSISDFQTLSNPVKTLAGSDRKFPLTITTSDNSVTVSSGLTKTSISNTIYTDKQVAIYEVDKVLVPKDLFPPAPPAPAPARPLAEPDPVAPRDASSALVIAWQHRVNVVLFGAGLYIAALVMDP |
| PtrFLA10 | MRPQSFILALSLIFFFLHCTKTLCQSPAAAPAMAPPKTPVKAPPADSSQAPSAQVATSPGPVDVNKILQKAGHFTVFARLMQATTEDTELNKELNTTNNGITILAPTDNAFSSLKAGFLNSLSDEDKTELVKFHVLPAFISTSQFQTVSNPVRTQAGTGPRVTLNVTTTGNFVNISSGLTNTSISGTVYTDSQLAIYQLDKVLFPLDIFTPKPPAPAPEPALGKPRKAAPDAESPTAPKDISGAPALLFLHNNALLLAVSCAFGAIIHS |
| PtrFLA11 | MAIALSSFNIFFTFLLVSTFHLGFSFSALQENHSNGTYSGQINSNSVLVALLDSHYTELAELVEKALLLQTLEDAVGKHNITIFAPKNEALERDLDPEFKRFLLEPGNLKSLQTLLLYHIVPNRINPSHNSSLQHHSTLCRDRVKLSSQESGEKLIDSAKIIQVNAVERPDGVIHGIERLLIPRSVQQDFNNRRSLQSISAVKPEGAPEVDPRTHRLKKPAPPAKPGSAPVLPIYDAMAPGPSLAPAPAPGPGGPHHHFNGEKQVKDFIETLLLYGGYNEMADILVNLTSLATEMGRLVSEGYVLTVLAPNDEAMAKLTTDQLSEPGAPEQIIYYHVIPEYQTEESMYNAVRRFGKISYDTLRLPHKVLAEEADGSVKFGHTENSAYLFDPDIYTDGRISVQGIDGVLFPLEEKEKSETKKEIKSVKVAVKPQRRGRLLEVACRMLGTFGQDSHFTTCQ |
| PtrFLA12 | MQRLTILLSLLFLLSTSTTFTRGHNITHILGKHPSFSTFNHYLTLTHLAGEINSRNTITVCAVDNAAMSELLSKHPSIATIKNILSLHVLLDYFGTKKLHQIREGTALAATMFQATGSAPGSTGFVNITDVKGGKVAFGPEDNEGNLDVFYVKSLEEIPYNISVIQISKVLPSDVAAAPTPEPSAMNITDIMSAHGCKVFADTLIANPEASKTYQDSVDGGLTVFCPLDDPFKAFFPKFKNLTASGKVSFLEFFGVPIYQSLAMLKSNNGIMNTLATDGEKKFDFTVQNDGEDVTLKTRSITAKIVGTLIDEQPLAIYTIDKVLLPKELFKAAPTPAPAPAPEKEVADAPKSSKHKKPSSDVVPSDSPADSPDGDLADQTADDNASVTLYGGRLVAMLLSLCSGLLLL |
| PtrFLA13 | MKHHFSVFLFPAILLLLHCTQTLSQTPTAAPAKAPAAASAPPPAATSSAQASPPVMVPVQVSKGPVNVIKILQKAGGFAVFIRLIKSTQEDIQVFSQLNDSRDGVTIFAPTDGAFSAIIKSGVLNSLSDHQKIELVQFHIIPKILTTANFQTVSNPITTLAGSGSRFALNVITTENMVNVTSGLTNTSVSAIVYTDSQLAVYQVDKVLLPLDIFAPKPLAPAPAPPKPKKDDGAESPLVPEDTSSAVSCIPLNSLIIFGAGMVAAVFTL |
| PtrFLA14 | MVPQFLLSFSLILSFLLHCPPTLAQSPAAAPGPPGPTNVTKILEKGGQFSVFIRLLKATQEDVTLNGQLNNTNNAITIFAPSDNAFSSLKSGTLNSLNDQEKAELVQFHIIPQYLSSSQFQTVSNPLTTQAGSGGRLELNVTTTGNSVNITTGLTNTSVSGTIYTDNQLAVYQVDKVLLPVDIFTPKPPTPAPAPEKPKKRSKAAESPDAPEDNSGAVSLTVLNDVVFFGVGIVAAIFSL |
| PtrFLA15 | MKQQYYSLFSFSFFLLFLHCTTTFAQTSPAATPAQAPAVVVAQPPAATPTQAAQPHGITNVTKILEKAGHFTIFIRLLRSTQEENHLFSALNDSSSGVTIFAPTDSAFSELKSGTLNTLSDGDKSELVKFHVVPTFLSTSQFQTVSNPLGTWAGTGNRLPLNVTSYPNSVNITTGLTNTSLSGTVYTDNQLAIYKIEKVLLPKDIFASKAPAPAPVAPAPEKPTKAVPAATVESPVAPVDTSSALMFTQNHVVGSVAIFAAAMFAL |
| PtrFLA16 | MATTPLSFFLLSLLSLSLNAQAQTPTAPAPTPSGPVNFTAVLVKGGQFATLIRLLNNTQTLNQIENQLNSSSEGMTIFAPTDNAFNNLKAGALNGLNQQEQVQLLQYHTLPKFYTMSNLLLVSNPVPTQASGQDGVWGLNFTGQSNQVNVSTGLVEVQINNALRQDSPLAVYPVDKVLLPEALFGVKPPTASPPAPSSKSNSTVAAAEPSTGKNSAGGRNVALGLVVGLGLVCMGILS |
| PtrFLA17 | MKQQYSIFSFSMLLLSLCYINTFAQSPTAAPAQAPAVVVAQPPVATPTQAAAPHGITNVTKILEKAGHFTIFIRLLRSTQEENHLFSALNDSNTGITIFAPTDSAFSELKSGTLNTLSDGDKSELVKFHVVPTFLSTSQFQTVSNPLGTWAGTGSRLPLNVTSYPNSVNITTGLTNTSLSGTVYTDNQLAIYKIEKVLLPKDIFGSNAPAPAPVQAPAEKPTKAVPSANVESPVAPVDISSAVTFMHNNVVGSLVIVAAAMFACNVEGF |
| PtrFLA18 | MKQQYSISSISVFLLFLHYTNTFAQSPAAAPAQAPAVVVAQPPAATPTQAAAPHGITNVTKILEKAGHFTIFIRLLRSTQEENHLFSALNDSSSGVTIFAPTDSAFSELKSGTLNTLSDGDKSELVKFHVVPTFLSTSQFQTVSNPLGTWAGTGSRLPLNVTSYPNSVNITTGLTNTSLSGTVYTDNQLAIYKIEKVLLPKDIFASKAPAPAPAPAREKPTKAVPAANVESPVAPVDISSAVTFMHNNVVVGSLVIVAAAMFACHVEGF |
| PtrFLA19 | MKQQHSLSSFSFFLLLLHCANTFAQSPAATPAQAPAAVVAQPPAATPTQAAQPHGITNVTKILEKAGHFTIFIRLLRSTQEENHLFSALNDSSSGVTIFAPTDSAFSELKSGTLNTLSDGDKSELVKFHVVPTFLSTSQFQTVSNPLGTWAGTGSRLPLNVTSYPNSVNITTGLTNTSLSGTVYTDNQLAIYKIEKVLLPKDIFASKAPAPAPVAPAPAKPTKAVPAATVESPVAPVDISSALMFAHNNVVGSVGIVAAAMFAL |
| PtrFLA20 | MATLQYSLLLSFTLSALVSTILAHNITDILSGFPEYSEFNKYLTQTKLADEINTRQTITVLALNNGAMTALAAKHPLSVIKNALSLLVVLDYYDPTKLHQISKGTTLSTTLYQTTGNAPGNLGFVNITDLQGGKVGFGSAAPGSKLDSSYTKSVKQVPYNISILEISQPIIAPGILTAPAPTPSSVNITALLEKAGCKTFASLLQTSGVIKTYQSAADKGLTIFAPNDEAFKAAGVPDLSKLTNAEIVSLLQYHATATYSPFGSLKTSKDPISTLASNGAGKFDLTVTSAGDSVTLHTGIGPSRVAETVLDSTPLVIFTVDNVLLPVELFGKAPSPAPAGEPVSAPSPSPVASSPAPASVEAPSPLAASPPAPPVETPGGAPAETPFGSENSTADGSAAVHVSVPVQVTVFATVICSILMS |
| PtrFLA21 | MKFSMIIVLSSTLLFSCTPLAYAQKVASPPAPTPTPSPAPAPSPPYVNLTDLLSVAGPFHNFLNYLESTKVIDTFQNQANNTDEGITIFVPKDDAFKNLKKASLSNLTQDQLKQLILFHALPHYYSLSDFKNLSQVSPVSTFAGAGGYALNFTDTSGTVHLDSGWSKTKVSSSVHSTDPVAIYQVDKVLLPEAIFGTNIPPTPAPAPAPDTSPTADSPTSDDSAGAGSAPGKSPPNSSYRINGVGIWSQLVLAIAGVLVLFL |
| PtrFLA22 | MPRPLPLLTLAISLVLLASTTTVNAHNITRILAKHPQFSTFNHYLTVTHLAAEINRRQTITVLALDNAAMSSLISKQLSVYTLRNVLSLHVLVDYFGTRKLHQITNGTELTATMFQATGSAPGASGYVNITDLNGGKVAFGAEDNDGKLNAVYVKSLEEIPYNISILQISQPLNSAEAEAPTAAPTLNVTAILSNQGCKAFSDLLIASGAHTTFEENVDGGLTVFCPTDPVINGFMPKYKNLTAPQKVSLLLYHGIPIYQSLQMLKTSNGIMNTLATNGANKYDFTVQNDGEVVTLETKVTTATITGTVKDEEPLVVYKINKVLLPRELFKAAPEKKAPAPKGEKDVADGPNADAPSDESDDQTADNDNGVNKMGGGRLAVVAPSFFFGVVMFFLFD |
| PtrFLA23 | MKQLISFSFSLVLLFLHCTQTLSQPPNAAPAKAPAAATVPPPAATSAQASPPVMVPVQVSKGPVNVIKILQKAGHFAFFTRLIKSTQEDIQLFSQLNDSRDGVTVFAPTDGAFSAIIKSGVLNSLTDHQKIELVQFHIIPRILTTANFQTVSNPITTLAGSGNRFALNVITTENMVNVTTGLTNTSVSAIVYTDSQLAIYQVDKVLLPLDIFAPKPLAPAPAPPKPKKDDGAESPMVPEDTSGSVICMVHNTLLMFGVGLVAAAIPL |
| PtrFLA24 | MVPQFLFSASFILFFLLHCPPTLAQSPAAAPAPPGPTNVTKVLEKGGQFSVFIRLLKATQEDVTLNGQLNNTNNAITIFAPSDNAFSSLKSGTLNSLSDQEKAELVQFHIIPQFLSSSQFQTVSNPLTTQAGSGGRLELNVTTTGNSVNITTGLTNTSVSGTIYTDNQLAVYQVDKVLLPLDIFTPKPPTPAPAPEKPKKRSKAAASPESPADTSGAVSFTVLNNVVFFGVCMVAAIYSL |
| PtrFLA25 | MDSHIYGVSEKTLFLFTLLCFSVASISALPHQNRTGNSTVTGQMINSNSVLVALLDSHYTELAELVEKALLLQTLEEAVGKHNITIFAPKNEALERQLDPEFKRFLLEPGNLKSLQTLLLFHIIPQRVGSNDWPGHKSNPTRHTTLCNDHLHLITKNSGKKLVGAAVLTRPDDVTRPDGVIHGIERLLVPQSVQEDFNRRRNLRSISAVLPEGAPEVDPRTHRLKKPEPPVRAGSPPVLPVYDAMSPGPSLAPAPAPGPGGPHHHFDGESQVKDFIQTLVHYGGYNEMADILVNLTSLATEMGRLVSEGYVLTVLAPNDEAMAKLTTDQLSEPGAPEQIIYYHIIPEYQTEESMYNAVRRFGKIGYDTLRLPHKVVAQEADGSVKFGSGDGSAYLFDPDIYTDGRISVQGIDGVLFPEVEKESTSVKKSVSSVKVATTTPRRGKLMEVACRMLGSLGQESHFTTCQ |
| PtrFLA26 | MRKQLLSPFVPFLMFFLYSSTTFAQTPSPAPSGPTNITAILAKAGQFTTLIRLLKSTQEADQINTQLNNSNQGLTVFAPTDNSFANLKAGTLNSLSDQQKVQLVQFHILPNFLSMSNFQTVSNPLRTQAGNSADGEFPLNVTTSGNQVNITTGVNTATVANTIYTDGQLVVYQVDQVLLPLDLFGTAPAPAPAPSKPEKDVPAKAPAGSKEDASVDSSGASIATVSFGVVLIAAISLKL |
| PtrFLA27 | MSTMLLFLLILLLISSSVLAASNPFSNAMEILSTSGYLSMALTLEITSKRLHLESSAATIFAPLDIAFARLGQLSVLDLQYHISPVRLSGYYLDSLPFGTRIPTLLPNHSLIVTTSLSYFDGKLSINGISIEESALVDFGSLIIFGMSEFFNSSLEISPNLTPAPAPSPSPVTSLGNTSQNESTGLDVDFFGQASHLLMPRGYSIMGTFLDAQLFGIKNQTRLTIFAPVDQAMDAYAKNVSDYSSIFRKHVVPGLFPRQDLEGFNDGTSLPTFSGGFMINLTKSGDVLVLNGVPVIFPDMYQSDWLIIHGLNQLLTPPLKEEELVGESFSELDGAEDKPDVLDFDDYVYGAP |
| PtrFLA28 | MGTQNLMINKSTAKILLHLLLLSLLHQITTATLTDQELDFALLSLRSYGYTLFPNAISTSDLRLQLLNQSSNATSTSTFTLFCPPDSLLFSVDLASTAPHYTKSLFLHVSPSRLSTSDLRNLTAASGGTYIDSLVPNHRLLITNSLAQLNGTVDGSILVNRVRVSVPDLFLGSDIAVHGLDGILVAGFDEKVEDTSFEAATWSPANAIGSAEQNSPLAGRFPARRRKGRNHRHNGRNGGIRRNNHRGRRINGGHRRGVGRNVSGGTRGGGVTRGAFAMYNHRL |
| PtrFLA29 | MEAFTTLLVLLMIKVLVCATSPTDIPSRSQDLVVASDEMARANYFSFVMLINMAPLDQKFQGNVTFLMPKDRLLSKIRMHQNAVSDFLLHHSIPSPLLFDHLRHIPPGSLIPSSDPDYMLNISNEGRKSFFLNNVKISSPDLCTAGSSIRCHGIDGVLLVDTDRHPLPACSNSTSPAIVATPPSPSLPLPDIPSFPSSAPPPGAAAPTDQEHIPKHSGSSQLESLSLGGLLKFMATSILVLNARVLYTVGQN |
| PtrFLA30 | MATSPLSLVLLSLFLSLSLHAQAQAPAAPAPAPSGPVNFTAVLVKGGQFVTFISLLNKTQTFNQIENQINSSSEGMTIFAPTDNAFSNLKSGALNGLSQQQQVQLLQYHMLPKFYSLSNLLLVSNPVPTQASGQEGVWGLNFTGQSNQVNVSTGLVEVQVNNALRQDFPLAVYPVDKVLLPDELFGVKPPSASPPAPATKGSSSGKSNSSDTAAEPSPGKNSAGGRNVALGLIFGLGFVSMGILS |
| PtrFLA31 | MKPQYLLSSFSIFLLFLHCPNTFAQSPAAAPAQAPAVVASPPAATPTQAAAPHGITNVTKILEKAGHFTIFIRLLRSTQDENRLFSALNDSSTGLTIFAPTDSAFSELKSGTLNTLSDGDKSELVKFHVVPNSYLLPSSRPLSGTVYTDNQLAIYKIEKVLLPKDIFASNAPAPAPVASAPEKPTKAVPAVTVESPAASVDISSALIFTHNLVVGSVGLLASAMFSL |
| PtrFLA32 | MKQQSISFFIFLLFLQCTYTFAQSPAAAPAQAPAVVVAQPPAATPTQAAAPHGITNVTKILEKAGHFTIFIRLLRSTQEENHLFSALNDSSTGLTIFAPTDSAFSELKSGTLNTLSDGDKSELVKFHVIPTFLSTSQFQTVSNPLGTWAGTGSRLPLNVTSYPNSVNITTGLTNTSLSGTVYTDNQLAIYKIEKVLLPKDIFASNAPAPAPVAPAPEKPAKAVPAANVESPVAPVDISSAVWFMHNNVAGSVGIVAAAVFAL |
| PtrFLA33 | MKPQYLLSSFSILLLFLHCTNTFAQSPAAAPAQAPAVVASPPAATPTQAAAPHGITNVTIILEKAGHFTIFIRLLRSTQEENHLFSALNDSSTGLTIFAPTDSAFSELKSGTLNTLSDGDKSELVKFHVVPTFLSTSQFQTVSNPLGTWAGTGSRLPLNVTSYPNSVNITTGLTNTSLSGTVYTDNQLAIYKIEKVLLPKDIFASNAPAPAPVAAAPEKPTKAVPAVTVESPAASVDISSALIFTHNLLVGSVGLLASAMFSL |
| PtrFLA34 | MKPQYLLSSFSIFLLFLHCPNTFAQSQAAAPAQAPAVVASPPAATLTQAAAPHGITNVTKILEKAGHFTIFIRLLRSTQEENHLFSALNDSSPGLTIFAPTDSAFSELKSGTLNTLSDGDKSQLVKFHVVPTFLSTSQFQTVVGYHLTSQSYTNSVNITTGLTNTSLSGTVYTDNQLAIYKIEKVLLPKDIFASNAPAPAPVAPAPEKPTKAVPAVTVESPAASVDISSALIFTHNLVVGSVGLLASAMFSL |
| PtrFLA35 | MKQQLISSFSIFLLFLHCASTFAQIPAAAPAQAPAVVVAPPPAATPTQAAAPHGITNVTKILEKAGHFTIFIRLLRSTQEESHLFSALNDSSTGLTIFAPTDSAFSELKSGTLNTLRDGDKSELVKFHVVPTFLSTSQFQTVSNPLGTWAGTGSRLPLNVTSYPNSVNITTGLTNTSLSGTVYTDNQLAIYKIEKVLLPKDIFTSNAPAPAPVAPAPEKPSKAVPAVTVESPAASVDISSALIFTNNILVGSFGLLASAMFSL |

Green: Signal peptide; Red:AGP-like glycosylated [region](app:ds:region)s;

Blue: Fasciclin domain; Purple: GPI-anchor protein;
