# Supplementary material for: Genome-Wide Analysis of the Fasciclin-Like Arabinogalactan Protein Gene Family Reveals Differential Expression Patterns, Localization, and Salt Stress Response in Populus
Source: Front Plant Sci. 2015 Dec 23;6:1140. doi: 10.3389/fpls.2015.01140 (PMC4688393; doi:10.3389/fpls.2015.01140)
Supplement: Supplementary file 3 [file Table3.DOC]

**Supplemental table 3.** Proline, Alanine, Serine, and Threonine (PAST) proportions in PtrFLAs(%)

| Protein | Proline | Alanine | Serine | Threonine | PAST |
| --- | --- | --- | --- | --- | --- |
| PtrFLA1 | 7.82 | 7.82 | 8.64 | 10.70 | 34.98 |
| PtrFLA2 | 6.16 | 10.10 | 7.88 | 8.87 | 33.02 |
| PtrFLA3 | 10.65 | 10.27 | 12.55 | 7.98 | 41.45 |
| PtrFLA4 | 10.45 | 10.07 | 8.96 | 10.82 | 40.30 |
| PtrFLA5 | 7.01 | 6.11 | 10.18 | 7.70 | 31.00 |
| PtrFLA6 | 7.53 | 10.88 | 8.79 | 10.46 | 37.66 |
| PtrFLA7 | 7.30 | 6.22 | 7.73 | 6.44 | 27.69 |
| PtrFLA8 | 7.13 | 7.56 | 8.42 | 4.54 | 27.65 |
| PtrFLA9 | 10.27 | 9.89 | 8.75 | 7.22 | 36.13 |
| PtrFLA10 | 10.04 | 12.27 | 8.55 | 10.04 | 40.90 |
| PtrFLA11 | 6.75 | 6.97 | 7.84 | 5.01 | 26.57 |
| PtrFLA12 | 6.07 | 9.07 | 8.58 | 8.58 | 32.30 |
| PtrFLA13 | 9.29 | 11.90 | 9.67 | 7.81 | 38.67 |
| PtrFLA14 | 8.75 | 7.50 | 9.58 | 9.58 | 35.41 |
| PtrFLA15 | 9.73 | 14.60 | 11.50 | 13.27 | 41.71 |
| PtrFLA16 | 7.98 | 9.24 | 9.24 | 7.98 | 34.44 |
| PtrFLA17 | 7.81 | 11.15 | 10.04 | 10.04 | 39.04 |
| PtrFLA18 | 7.81 | 12.64 | 10.41 | 9.67 | 40.53 |
| PtrFLA19 | 8.33 | 13.64 | 10.61 | 9.85 | 42.43 |
| PtrFLA20 | 8.79 | 11.64 | 11.40 | 10.21 | 42.04 |
| PtrFLA21 | 10.69 | 9.54 | 12.21 | 8.02 | 40.46 |
| PtrFLA22 | 5.85 | 10.37 | 6.12 | 10.11 | 30.72 |
| PtrFLA23 | 9.36 | 10.86 | 7.87 | 8.61 | 36.70 |
| PtrFLA24 | 8.75 | 9.17 | 10.00 | 10.00 | 37.92 |
| PtrFLA25 | 7.30 | 6.22 | 7.51 | 6.65 | 27.68 |
| PtrFLA26 | 7.53 | 10.46 | 9.62 | 10.04 | 37.65 |
| PtrFLA27 | 7.62 | 6.62 | 14.24 | 6.62 | 30.10 |
| PtrFLA28 | 3.53 | 7.42 | 10.25 | 8.48 | 29.68 |
| PtrFLA29 | 10.32 | 5.95 | 12.70 | 3.97 | 32.94 |
| PtrFLA30 | 8.16 | 9.00 | 12.65 | 4.90 | 34.71 |
| PtrFLA31 | 9.25 | 13.22 | 11.89 | 7.49 | 41.85 |
| PtrFLA32 | 8.40 | 13.74 | 9.54 | 9.54 | 41.22 |
| PtrFLA33 | 7.98 | 12.55 | 11.41 | 10.65 | 42.59 |
| PtrFLA34 | 7.54 | 12.30 | 11.51 | 9.92 | 41.27 |
| PtrFLA35 | 8.37 | 12.17 | 11.41 | 10.27 | 42.22 |
