# Supplementary material for: Genome-Wide Analysis of the Fasciclin-Like Arabinogalactan Protein Gene Family Reveals Differential Expression Patterns, Localization, and Salt Stress Response in Populus
Source: Front Plant Sci. 2015 Dec 23;6:1140. doi: 10.3389/fpls.2015.01140 (PMC4688393; doi:10.3389/fpls.2015.01140)
Supplement: Supplementary file 4 [file Table4.DOC]

**Supplemental table 4.** Conserved motifs predicted by MEME program in PtrFLA

| Motif | Width | Best possible match |
| --- | --- | --- |
| 1 | 70 | TKILEKAGHFTIFIRLLRSTQEENHLMSQLNDSNYGITIFAPTDNAFSELKSGTLNTLSDQEKSEYVKFH |
| 2 | 50 | GRWPLNVTTYGNSVNITTGWTNTSISGTVYTDNQLAIYQIDKVLLPKDIF |
| 3 | 42 | QQYMIISFSWFLLFLHCTNTFCQSPAAAPAQAPAVVVAPPP |
| 4 | 42 | PAPAPVAPAPEKPTKRVPAATVESPVAPVDISSALWFMHNNV |
| 5 | 70 | EYQTEESMYNAVRRFGKIGYDTLRLPHKVLAQEADGSVKFGHGEGSAYLFDPDIYTDGRISVQGIDGVLF |
| 6 | 70 | HYTELAELVEKALLLQTLEEAVGKHNITIFAPRNEALERDLDPEFKRFLLEPGNLKSLQTLLLYHIIPQR |
| 7 | 70 | RPDGVIHGIERLLIPRSVQEDFNRRRNLRSISAVKPEGAPEVDPRTHRLKKPEPPVRPGSPPVLPIYDAM |
| 8 | 70 | DYFGTKKLHQIRDGTALAATMFQATGSAPGSTGFVNITDVKGGKVAFGPEDNGGKLDVFYVKSVEEIPYN |
| 9 | 40 | KKEIKSVKVATKPQRRGKLMEVACRMLGTFGQDSHFTTCQ |
| 10 | 53 | GHNITHILGKHPEFSTFNHYLTLTHLAGEINRRQTITVCAVDNAAMSELLSKH |
| 11 | 70 | AHKTYQDSVDGGLTVFCPLDDPFKAFMPKFKNLTASGKVSLLQFFGVPIYQSLAMLKSNNGIMNTLATNG |
| 12 | 30 | APGPSLAPAPAPGPGGPHHHFDGERQVKDF |
| 13 | 30 | DWPGHKSNPHRHSTLCRDRIHLISKNSGKK |
| 14 | 30 | IYVLFLMLHCYSQTAQAPAPAPAPMGPTNI |
| 15 | 42 | APAPAPPKPEKDDGAEAPMGPKDDSSAVSCMMHNATVMFGVG |
| 16 | 40 | ADSPDDDSADQTAEPNSGYRIYGGRIWAMLLIACLGLVCM |
| 17 | 30 | ARPHQQRNHNNGGYGGMINSNSVLVALLDS |
| 18 | 32 | LQYHIIPGRLSFDYLRHLPFGTRIPTLDPDHC |
| 19 | 30 | PPPPEEEVADAPKSSKHKKPSADDVPSDSP |
| 20 | 57 | NRVRVKVPDVMRGWDIVVHGVDGIFPRGHDEKVEDFDEMMGIWGEGQNIGAAEDGAC |
