# Supplementary material for: Genome-Wide Analysis of the Fasciclin-Like Arabinogalactan Protein Gene Family Reveals Differential Expression Patterns, Localization, and Salt Stress Response in Populus
Source: Front Plant Sci. 2015 Dec 23;6:1140. doi: 10.3389/fpls.2015.01140 (PMC4688393; doi:10.3389/fpls.2015.01140)
Supplement: Supplementary file 5 [file Table5.DOC]

***Supplemental table 5.*** *Probes of PtrFLA genes in Populus* *trichocarpa*

| Gene symbol | Gene Locus | Probe set |
| --- | --- | --- |
| *PtrFLA1* | Potri.001G320800 | PtpAffx.249.461.S1_s_at |
| *PtrFLA2* | Potri.001G367900 | [Ptp.4554.1.S1_s_at](https://www.affymetrix.com/LinkServlet?probeset=Ptp.4554.1.S1_s_at&array=Poplar) |
| *PtrFLA3* | Potri.002G223300 | [PtpAffx.9094.2.S1_at](https://www.affymetrix.com/LinkServlet?probeset=PtpAffx.9094.2.S1_at&array=Poplar) |
| *PtrFLA4* | Potri.004G210600 | [PtpAffx.33081.1.S1_at](https://www.affymetrix.com/LinkServlet?probeset=PtpAffx.33081.1.S1_at&array=Poplar) |
| *PtrFLA5* | Potri.005G079500 | [PtpAffx.205197.1.S1_at](https://www.affymetrix.com/LinkServlet?probeset=PtpAffx.205197.1.S1_at&array=Poplar) |
| *PtrFLA6* | Potri.006G129200 | [PtpAffx.25139.1.A1_s_at](https://www.affymetrix.com/LinkServlet?probeset=PtpAffx.25139.1.A1_s_at&array=Poplar) |
| *PtrFLA7* | Potri.006G200300 | NONE |
| *PtrFLA8* | Potri.008G012400 | [Ptp.2510.2.S1_s_at](https://www.affymetrix.com/LinkServlet?probeset=Ptp.2510.2.S1_s_at&array=Poplar) |
| *PtrFLA9* | Potri.009G012100 | [Ptp.3083.1.S1_s_at](https://www.affymetrix.com/LinkServlet?probeset=Ptp.3083.1.S1_s_at&array=Poplar) |
| *PtrFLA10* | Potri.009G012200 | [PtpAffx.162047.1.S1_s_at](https://www.affymetrix.com/LinkServlet?probeset=PtpAffx.162047.1.S1_s_at&array=Poplar) |
| *PtrFLA11* | Potri.010G244900 | PtpAffx.210358.1.S1_s_at |
| *PtrFLA12* | Potri.011G093500 | NONE |
| *PtrFLA13* | Potri.012G015000 | [PtpAffx.142854.1.S1_s_at](https://www.affymetrix.com/LinkServlet?probeset=PtpAffx.142854.1.S1_s_at&array=Poplar) |
| *PtrFLA14* | Potri.012G127900 | Ptp.5517.1.S1_a_at |
| *PtrFLA15* | Potri.013G014200 | [PtpAffx.141260.1.S1_x_at](https://www.affymetrix.com/LinkServlet?probeset=PtpAffx.141260.1.S1_x_at&array=Poplar) |
| *PtrFLA16* | Potri.013G120600 | [Ptp.963.1.A1_s_at](https://www.affymetrix.com/LinkServlet?probeset=Ptp.963.1.A1_s_at&array=Poplar) |
| *PtrFLA17* | Potri.013G151300 | [Ptp.371.1.S1_at](https://www.affymetrix.com/LinkServlet?probeset=Ptp.371.1.S1_at&array=Poplar) |
| *PtrFLA18* | Potri.013G151400 | [PtpAffx.211342.1.S1_at](https://www.affymetrix.com/LinkServlet?probeset=PtpAffx.211342.1.S1_at&array=Poplar) |
| *PtrFLA19* | Potri.013G151500 | [PtpAffx.141260.1.S1_x_at](https://www.affymetrix.com/LinkServlet?probeset=PtpAffx.141260.1.S1_x_at&array=Poplar) |
| *PtrFLA20* | Potri.014G071700 | [Ptp.4674.1.S1_x_at](https://www.affymetrix.com/LinkServlet?probeset=Ptp.4674.1.S1_x_at&array=Poplar) |
| *PtrFLA21* | Potri.014G162900 | PtpAffx.9094.1.S1_at |
| *PtrFLA22* | Potri.014G168100 | PtpAffx.211930.1.S1_at |
| *PtrFLA23* | Potri.015G013300 | [Ptp.3058.1.S1_s_at](https://www.affymetrix.com/LinkServlet?probeset=Ptp.3058.1.S1_s_at&array=Poplar) |
| *PtrFLA24* | Potri.015G129400 | [PtpAffx.5437.1.A1_s_at](https://www.affymetrix.com/LinkServlet?probeset=PtpAffx.5437.1.A1_s_at&array=Poplar) |
| *PtrFLA25* | Potri.016G066500 | PtpAffx.358.1.S1_at |
| *PtrFLA26* | Potri.016G088700 | [Ptp.792.1.S1_at](https://www.affymetrix.com/LinkServlet?probeset=Ptp.792.1.S1_s_at&array=Poplar) |
| *PtrFLA27* | Potri.017G111600 | PtpAffx.215438.1.S1_at |
| *PtrFLA28* | Potri.019G002300 | PtpAffx.215073.1.S1_at |
| *PtrFLA29* | Potri.019G049600 | [PtpAffx.212193.1.S1_at](https://www.affymetrix.com/LinkServlet?probeset=PtpAffx.212193.1.S1_at&array=Poplar) |
| *PtrFLA30* | Potri.019G093300 | PtpAffx.36865.1.A1_at |
| *PtrFLA31* | Potri.019G120900 | Ptp.785.2.S1_x_at |
| *PtrFLA32* | Potri.019G121100 | NONE |
| *PtrFLA33* | Potri.019G121200 | Ptp.785.2.S1_x_at |
| *PtrFLA34* | Potri.019G122800 | Ptp.785.2.S1_x_at |
| *PtrFLA35* | Potri.019G123200 | [Ptp.785.2.S1_x_at](https://www.affymetrix.com/LinkServlet?probeset=Ptp.785.2.S1_x_at&array=Poplar) |
